# Effects of NaCl Concentrations on Growth Patterns, Phenotypes Associated With Virulence, and Energy Metabolism in *Escherichia coli* BW25113

**DOI:** 10.3389/fmicb.2021.705326

**Published:** 2021-08-16

**Authors:** Fen Li, Xue-Song Xiong, Ying-Ying Yang, Jun-Jiao Wang, Meng-Meng Wang, Jia-Wei Tang, Qing-Hua Liu, Liang Wang, Bing Gu

**Affiliations:** ^1^Medical Technology School of Xuzhou Medical University, Xuzhou, China; ^2^School of Life Sciences, Xuzhou Medical University, Xuzhou, China; ^3^Department of Bioinformatics, School of Medical Informatics and Engineering, Xuzhou Medical University, Xuzhou, China; ^4^Jiangsu Key Laboratory of New Drug Research and Clinical Pharmacy, School of Pharmacy, Xuzhou Medical University, Xuzhou, China; ^5^Department of Pharmaceutical Analysis, School of Pharmacy, Xuzhou Medical University, Xuzhou, China; ^6^State Key Laboratory of Quality Research in Chinese Medicines, Macau University of Science and Technology, Taipa, China; ^7^Institut Pasteur of Shanghai, Chinese Academy of Sciences, Shanghai, China; ^8^Laboratory Medicine, Guangdong Provincial People’s Hospital, Guangdong Academy of Medical Sciences, Guangzhou, China

**Keywords:** *Escherichia coli*, bacterial survival, biofilm formation, glycogen, trehalose, transcriptome

## Abstract

According to the sit-and-wait hypothesis, long-term environmental survival is positively correlated with increased bacterial pathogenicity because high durability reduces the dependence of transmission on host mobility. Many indirectly transmitted bacterial pathogens, such as *Mycobacterium tuberculosis* and *Burkhoderia pseudomallei*, have high durability in the external environment and are highly virulent. It is possible that abiotic stresses may activate certain pathways or the expressions of certain genes, which might contribute to bacterial durability and virulence, synergistically. Therefore, exploring how bacterial phenotypes change in response to environmental stresses is important for understanding their potentials in host infections. In this study, we investigated the effects of different concentrations of salt (sodium chloride, NaCl), on survival ability, phenotypes associated with virulence, and energy metabolism of the lab strain *Escherichia coli* BW25113. In particular, we investigated how NaCl concentrations influenced growth patterns, biofilm formation, oxidative stress resistance, and motile ability. In terms of energy metabolism that is central to bacterial survival, glucose consumption, glycogen accumulation, and trehalose content were measured in order to understand their roles in dealing with the fluctuation of osmolarity. According to the results, trehalose is preferred than glycogen at high NaCl concentration. In order to dissect the molecular mechanisms of NaCl effects on trehalose metabolism, we further checked how the impairment of trehalose synthesis pathway (*otsBA* operon) via single-gene mutants influenced *E. coli* durability and virulence under salt stress. After that, we compared the transcriptomes of *E. coli* cultured at different NaCl concentrations, through which differentially expressed genes (DEGs) and differential pathways with statistical significance were identified, which provided molecular insights into *E. coli* responses to NaCl concentrations. In sum, this study explored the in vitro effects of NaCl concentrations on *E. coli* from a variety of aspects and aimed to facilitate our understanding of bacterial physiological changes under salt stress, which might help clarify the linkages between bacterial durability and virulence outside hosts under environmental stresses.

## Highlights

- Elevated NaCl concentration generally inhibits *E. coli* growth and phenotypes associated with virulence, such as biofilm formation, oxidative resistance and motile ability.
- Elevated NaCl concentration reduces glucose consumption and glycogen accumulation while improving trehalose production.
- Disruption of trehalose synthesis pathway OtsAB completely abolishes trehalose accumulation and greatly suppresses glycogen accumulation.
- Disruption of trehalose synthesis pathway OtsAB facilitates biofilm formation at low NaCl concentrations.
- Transcriptomic study provides clues into the molecular mechanisms of *E. coli* responses to the alteration of NaCl concentrations.

## Introduction

Bacterial pathogens must have certain routes to be transmitted from one host to another, either directly through physical contact or indirectly via an intermediate agent. Although bacterial transmission is central to host infection, the transmission modes are complex and diverse, which involves direct host-to-host transmission, vector-borne transmission, and environmental transmission, and are often under genetic control of the host or the pathogen (Antonovics et al., 2017). According to the conventional wisdom of virulence evolution, all pathogens should be in a relationship of commensalism or mutualism with their hosts, that is, being avirulent to their hosts, because no pathogen wants to “kill the chicken for the eggs” (Wang et al., 2017). However, this traditional idea started to be challenged in mid-20th century and it was argued that avirulence should not be so inevitable for pathogens (Alizon et al., 2009). Later, more and more studies indicated that linkages existed between bacterial transmission modes and the evolution of bacterial virulence, one of the reasonings behind which is that increasing bacterial abundance within-host promotes the potential of pathogen transmission, but also increases host mortality (Cressler et al., 2015).

Through a series of cross-species studies, it was observed that vector-borne pathogens, such as Yellow Fever virus and *Rickettsia prowazekii* etc., tended to be more virulent than directly transmitted diseases like Coronavirus and *Haemophilus influenzae* since vector-borne pathogens did not rely on the well beings of hosts for transmission (Ewald, 1983). However, further studies showed that some non-vector-borne pathogens such as *Mycobacterium tuberculosis* and *Corynebacterium diphtheriae* could also cause high mortality in hosts (Ewald, 1983; Walther and Ewald, 2004). Epidemiological studies revealed that the high virulence of non-vector-borne pathogens were positively correlated with the long-term survival time in the environment because high durability increased transmission opportunities with less reliance on host mobility, which was later termed as the sit-and-wait hypothesis (Walther and Ewald, 2004). Thus, long-term environmental survival could play important roles in bacterial pathogenicity (Wang et al., 2017). In particular, when being outside the hosts, bacterial pathogens constantly encounter abiotic stresses such as nutrient deficiency, temperature fluctuation, and osmotic imbalance, etc. (Guan et al., 2017). It is possible that abiotic stresses may activate certain pathways or the expressions of certain genes, which also contributions to bacterial virulence such as self-replication, cellular adhesion and host invasion, etc. and is worthy of further investigation since it may provide molecular insights into the changing patterns of bacterial pathogenicity (Boor, 2006).

**FIGURE 1:**
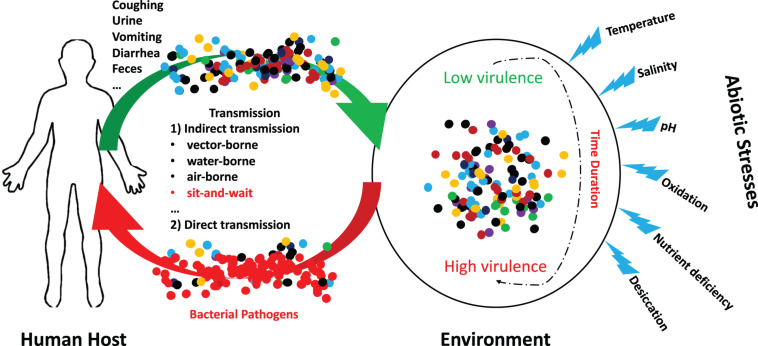
Schematic illustration of the potential interactions between abiotic stress and bacterial pathogens. A group of indirectly transmitted bacterial pathogens shed from human hosts were spread to environment via a variety of routes, where they encounter many abiotic stresses for a certain duration. During the period, it is possible that bacterial pathogenic phenotypes could be altered from low virulence to high virulence due to the influences of abiotic stresses. These bacteria may cause severe consequences after being transmitted to new hosts.

In fact, a variety of studies have revealed that abiotic stresses could improve virulent phenotypes of bacterial pathogens. For example, Sundberg et al. explore how long-term starvation influences virulence of the environmentally transmitting fish pathogen *Flavobacterium columnare*, which found that starved rhizoid isolates evolved toward significantly higher virulence after 5 months when compared with the ancestral rhizoid (Sundberg et al., 2014). Mihaljevic et al. studied the effects of environmental stress factors on the survival and virulence of *Campylobacter jejuni*, according to which oxygen exposure increased invasion capability and intraepithelial survival of the clinical isolate (Mihaljevic et al., 2007). Thus, it concludes that aeration is associated with the pathogenic potential of *C. jejuni* (Mihaljevic et al., 2007). In addition, Pinto et al. used RNA sequencing to identify the differentially expressed genes of *Corynebacterium pseudotuberculosis* in response to abiotic stresses, which uncovered that, under thermal, osmotic or acidic stress conditions, biological processes such as cellular adhesion and/or oxidoreduction become the most evident in terms of induced genes (Pinto et al., 2014). Recently, Vanaporn and Titball (2020) systematically reviewed the functions of trehalose in bacterial virulence, according to which trehalose biosynthesis is typically induced when exposed to abiotic stress while threhalose is associated with host colonization, within-host growth, and the modulation of host defense mechanisms. For a schematic illustration of the potential interactions between abiotic stress and bacterial pathogens, please refer to Figure 1.

Among these abiotic stresses, fluctuating osmotic pressure is commonly encountered by bacterial pathogens. Dinnbier et al. (1988) first revealed that *E. coli* K-12 transiently accumulated potassium glutamate for elevated NaCl concentration and then replaced it with trehalose as an osmoprotectant during adaptation. Later, a variety of studies investigated how bacteria responded to the stress and showed the corresponding effects on bacterial physiology like virulence (Strom and Kaasen, 1993; Sleator and Hill, 2002; Macintyre et al., 2020). In this study, we used *Escherichia coli* K-12 BW25113 as a model organism to comprehensively explore how NaCl concentrations influenced growth pattern, biofilm formation ability, oxidative stress resistance, and motile capacity, all of which were associated with bacterial environmental survival and within-host pathogenicity. In addition, since energy metabolism also contributed to osmotic pressure response, we investigated how glycogen and trehalose metabolisms were influenced by different NaCl concentrations and their potential interconnections, which revealed that glycogen level was significantly reduced while trehalose level was greatly increased in response to high NaCl concentrations. In order to further understand the osmoprotection roles of trehalose, we investigated the physiological changes of two single-gene knockout strains, *E. coli* BW25113 Δ*otsA* and Δ*otsB*, under salt stresses, respectively. Moreover, for elucidating the molecular mechanisms of *E. coli* response to osmotic stress, we compared transcriptomes of *E. coli* cultured under different NaCl concentrations. Differentially expressed genes (DEGs) with statistical significance, together with differential metabolic pathways, were identified, which might provide insights into the relationship between environmental durability and bacterial virulence. In sum, our study showed that salt stress could greatly and significantly alter bacterial phenotypes, which not only facilitated our understanding of physiological changes under NaCl concentrations in bacteria, but also provided clues for the evolution of bacterial virulence outside hosts from the perspective of the sit-and-wait hypothesis.

## Materials and Methods

### Bacterial Strains and Growth Conditions

The commonly used lab strain *Escherichia coli* K-12 BW25113 (Horizon Discovery Ltd., United States) and two single-gene knockout mutants, *E. coli* K-12 BW25113 Δ*otsA* and K-12 BW25113 Δ*otsB*, as part of the KEIO collection (Horizon Discovery Ltd., United States) were studied in this project. Inactivation of the two genes at expression level was double-checked via quantitative reverse-transcription PCR (qRT-PCR). The strains were initially stored in ultra-low temperature freezer (Thermo Fisher, United States) at −80°C. When doing experiment, the strains were thawed on ice, streaked on Luria–Bertani (LB) agar (VICMED, China) plates, and incubated at 37^*o*^C overnight for recovery. Single colonies were picked up from the agar plate, which were then used to inoculate LB liquid medium culture and 1 × M9 minimal medium culture (Sigma-Aldrich, United States) consisting of 6.78 g/L Na_2_HPO_4_, 3 g/L KH_2_PO_4_, 1 g/L NH_4_Cl, and 0.5 g/L NaCl for subsequent experiments as designated.

### Measurement of Bacterial Growth Curves

**FIGURE 2:**
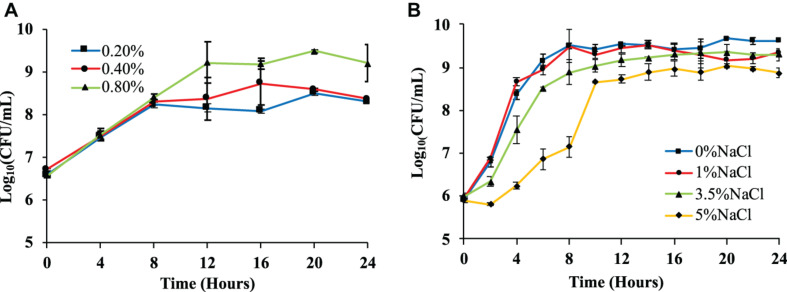
Effects of glucose and NaCl concentrations on the growth rates of *Escherichia coli* BW25113. **(A)** *E. coli* growth curves in 1 × M9 minimal medium with three different glucose concentrations (0.2, 0.4, and 0.8%) measured via logarithmic value of CFU with base 10. **(B)** *E. coli* growth curves in LB broth supplemented with four different NaCl concentrations (0, 1, 3.5, and 5%) measured via logarithmic value of CFU with base 10. Three independent replicates were performed for the number of viable cells, the average values and standard error means were used to draw the curves.

A single colony (wild-type, Δ*otsA*, Δ*otsB*) was picked up from the LB agar plate and inoculated into 10 mL of LB broth. After vortexing, the culture was incubated overnight at 37°C and 220 rpm. The overnight culture was then used to inoculate fresh LB broth at the ratio of 1:100, which was cultured for 4 h at 37°C and 220 rpm. For the effects of glucose concentrations on bacterial growth, the culture was used to inoculate 1 × M9 minimal medium supplemented with 0.2, 0.4, and 0.8% glucose at a ratio of 1:20, respectively, which was then cultured at 37°C and 220 rpm for 24 h. For the effects of NaCl, the culture was used to inoculate sterile LB broth containing 0%, 1%, 3.5%, and 5% NaCl at the ratio of 1:1000. At 0, 2, 4, 6, 8, 10, 12, 14, 16, 18, 20, 24 h, the optical density value OD_600_ was measured via microplate reader while the number of viable cells (colony forming units per milliliter, CFU/mL) was counted via Miles and Misra method for the two tests, respectively (Hedges, 2002). Three independent replicates were performed for the number of viable cells, the average of which, together with standard error means, was used to draw growth curve (Figure 2). As for the OD_600_-based growth curves for the two tests, please refer to Supplementary Figure 1.

### Quantification of Biofilm Formation

The quantification of bacterial biofilm formation of *E. coli* strains (wild-type, Δ*otsA*, Δ*otsB)* was performed by following the procedures as previously described by Wang et al. (2015) with modifications. In particular, a single *E. coli* colony was picked up from LB agar plate and inoculated into 10 mL LB broth, which was then cultured overnight at 37°C and 220 rpm. Fresh LB broth with different salt concentrations (0, 1, 3.5, 5%) were mixed with the overnight bacterial culture at the ratio of 100:1. Take 200 μL solution from each of the above-prepared LB broths as experimental group, together with 200 μL solution of sterile LB broth with corresponding salt concentration as control group to a 96-well plate. For each sample, three technical repeats and three biological repeats were performed. The 96-well plates were incubated statically at 30°C for 20 h. After that, bacterial solutions were discarded and the plates were thoroughly but gently washed with deionized water for three times. Then, 200 μL of 0.1% crystal violet solution (Dalian Meilun Biotechnology Co. Ltd., China) was added to each well for a 10-min incubation at room temperature in order to stain the biofilm. After staining, all the solutions were discarded and the plates were washed with deionized water for three times. In order to quantify biofilm formation capacity, 200 μL of the mixed solution with 20% ethanol and 80% acetone (Sinopharm Chemical Reagent Co. Ltd., China) was added to each well for 10 min for decolorization. 150 μL of the solution from each well was then transferred to a new 96-well plate. A microplate reader (Thermo Fisher, United States) was used to measure the absorbance of the solution at 590 nm, the values of which represented bacterial biofilm formation abilities.

### Measurement of Oxidative Stress Resistance

A single colony (wild-type, Δ*otsA*, Δ*otsB*) was picked up from the LB agar plate and inoculated into 10 mL LB broth for overnight culture at 37°C and 220 rpm. The bacterial solution was then mixed with fresh LB broth at a ratio of 1:100, which was cultured for 4 h at 37°C and 220 rpm. After that, 20 μL bacterial solutions were well mixed with LB broths containing 0.8% agar and different concentrations of NaCl (0, 1, 3.5, 5%), which were then poured into 90 mm petri dishes and solidified. 10 μL H_2_O_2_ solution (6.6 mol/L) was then dropped onto 6 mm round filter paper sheet that was placed in the center of each petri dish. The plates were then incubated statically overnight at 37°C. The diameter of the inhibition zone from three different directions was recorded. The experiment for each sample was repeated for three times independently. The average value and standard error were present for each sample.

### Motility Test

Single colonies (wild-type, Δ*otsA*, Δ*otsB*) picked up from LB agar plate were used to inoculate 10 mL LB broth that was further cultured at 37°C and 220 rpm overnight. 100 mL fresh LB broth was inoculated with the overnight culture at a ratio of 100:1, which was then cultured for 4 h at 37°C and 220 rpm (starting culture). Centrifuge the culture at 6,000 *g* for 10 min, discard the supernatant, resuspend bacterial pellet in LB broth to reach a final OD_600_ of 2. Transfer 2 μL of the resuspended culture to the center of LB agar plates (0.3% agar) with different NaCl concentrations (0, 1, 3.5, 5%), which were then incubated statically at 37°C for 24 h. Diameters of the near-circle growth zones were measured from three different directions, which reflected bacterial motile abilities. The experiment was repeated independently for three times. Averaged diameter and standard errors were visualized and present.

### Quantification of Glucose Consumption Rate

Starting culture was prepared as described above, which was then inoculated (ratio = 20:1) into 1 × M9 minimal medium containing 0.8% glucose and different concentrations of NaCl (0, 1, 3.5, 5%). The mixed culture was incubated at 37°C with shaking rate of 220 rpm. At 16 h, 20 h, and 24 h, 1 ml of culture was taken out for centrifugation at 6,000 *g* for 10 min, respectively. It is noteworthy that for the two *E. coli* mutants Δ*otsA* and Δ*otsB*, the experiment was only performed at 20 h. 10 μL of the supernatant was transferred into an Eppendorf (EP) tube with 990 μL GOPOD solution (Megazyme, Ireland). The tube was vortexed and incubated at 50°C for 20 min. After reaction, the absorbance value was measured at 510 nm, which was then transformed into glucose content in the culture (mg/mL) by using glucose standard curve produced by following the manufacturer’s instructions. Since the initial glucose content (0.8%) was known, the percentage of left-over glucose was easily calculated. The experiment was repeated for three times independently. Averaged values and standard errors were visualized and present.

### Glycogen/Protein Ratio Assay

Wild-type *E. coli* BW25113 was incubated in 1 × M9 minimum medium containing 0.8% glucose and different concentrations of NaCl (0, 1, 3.5, 5%) for 16, 20, and 24 h. It is noteworthy that for the two *E. coli* mutants Δ*otsA* and Δ*otsB*, the experiment was only performed at 20 h. 10 mL of the culture was then centrifuged at 6,000 *g* for 10 min. The bacterial pellet was resuspended in 1 mL glycogen extraction buffer (50 mM Tris, pH 8, 150 mM NaCl, 2 mM EDTA, 50 mM NaF, 5 mM sodium pyrophosphate, and Roche protease inhibiting cocktail) and lysed by sonication (25% amplitude, 10 s on, 30 s off, 3 repeats). Two groups were set-up for further analysis, which were experimental group and blank control group. In the experimental group, 20 μL of lysed bacterial culture was mixed with 100 μL of pH 4.5 acetic acid-sodium acetate buffer solution, and then mixed with 5 μL of 3,260 U/mL amyloglucosidase (MegaZyme, Ireland), which was made up to 500 μL with deionized water. Composition of the blank control group was exactly the same as that of the experimental group except that amyloglucosidase was not added. After vortexing, samples were incubated at 50°C for 30 min. After the reaction, centrifuge the samples at 6,000 *g* for 10 min at room temperature. After centrifugation, take 300 μL of the supernatant to mix with 700 μL of GOPOD reagent, which was then incubated at 50°C for 20 min. When the reaction complete, take 150 μL of the sample in a 96-well plate (three technical repeats for each sample), and use a microplate reader to determine the absorbance values of the experimental group and the blank control sample at 510 nm. The absorbance value of the experimental group is subtracted from the absorbance value of the blank control group, which was then brought into the standard curve to obtain the glycogen content. As for protein content determination, we used commercial Bradford method kit (Shanghai Biyuntian Biotechnology Co., Ltd., China). All the procedures were done by following the manufacturer’s instructions. Briefly, bacterial homogenate was diluted 20 times with deionized water, 5 μL of which was then added to a 96-well plate. 250 μL of G250 staining solution was added to each well. Measure the absorbance with a microplate reader at of 595 nm. Protein content was obtained by using standard protein curve. Glycogen/protein ratio was calculated by simple division. All the procedures were repeated for three times, independently.

### Trehalose Content Assay

Commercial trehalose assay kit (Megazyme, Ireland) was used for its content determination. Manufacturer’s instructions were followed for content quantification. Briefly, 200 μL of lysed bacterial homogenate as described above in section 2.7 was mixed with 200 μL of alkaline sodium borohydride, which was stirred and reacted in a metal bath at 40°C for 30 min. 500 μL of 200 mM glacial acetic acid was added to the mixed and vortexed to remove excess sodium borohydride. After 5 min, add 200 μL of 2 M imidazole buffer, and adjust the solution to pH 7. Take 20 μL of the neutral solution to a 96-well plate, add 20 μL kit buffer, 10 μL NADPH + /ATP, 2 μL HK/G-6-PDH, and 200 μL ddH_2_O to each well. Mix and react at 25°C for 5 min. After that, absorbance value (A1) was measured at 340 nm. Add 2 μL trehalase to each well. Mix and react for 5 min at 25°C. Measure the absorbance value (A2) of the sample at 340 nm. Obtain the final value by subtracting A2 with A1 (A2-A1). The trehalose content of the sample was calculated based on the standard trehalose curve. The tests were repeated independently for three times.

### Comparative Transcriptome Analysis

#### Bacterial Culture and RNA Extraction

A single colony of wild-type *E. coli* BW25113 was picked up from LB agar plate, which was used to inoculate 10 mL of fresh LB broth. The inoculated medium was well mixed and incubated overnight at 37°C and 220 rpm, which was then added to fresh LB broth at the ratio of 1:100 for 4-h incubation at 37°C and 220 rpm. Fresh 1 × M9 minimal media (0.8% glucose) containing 0%, 1%, 3.5%, 5% NaCl were then mixed with the 4-h bacterial culture at a ratio of 20:1. The four groups of mixed culture were incubated for 20 h at 37°C and 220 rpm. After culture, transfer 1 mL of the bacterial solution into a 2 mL sterile tube, centrifuge the solution at 6,000*g*, 4°C for 10 min, discard the supernatant and retain the pellet for RNA extraction. Total RNA was extracted via Total RNA Extractor Kit (Sangon, Shanghai) by following the manufacturer’s instructions, which was then treated with RNase-free DNase I in order to get rid of genomic DNA contaminant. RNA completeness was assessed with 1.0% agarose gel. The quality and quantity of RNA were then evaluated by using Qubit Flurometer (Life Technologies, CA, United States). High quality RNA samples were sent submitted to Sangon Biotech Co., Ltd. (Shanghai, China) for library preparation. Paired-end sequencing of the library was performed at Sangon Biotech Co., Ltd. on the HiSeq XTen sequencers (Illumina, San Diego, CA, United States).

#### Transcriptome Analysis

##### Data assessment and reference genome alignment

FastQC (version 0.11.2) was used for evaluating the quality of sequenced data. Raw reads were filtered by Trimmomatic (version 0.36), which involved deletions of adaptor sequence, low quality bases (Q < 20), and short reads (< 35 nt). Clean reads were then used for further analysis through mapping to the reference genome *Escherichia coli* K-12 substr. MG1655 (NCBI Genome ID NC_000913.3) by HISAT2 (version 2.0) with default parameters. RSeQC (version 2.6.1) was used to statistically check the alignment results. The homogeneity distribution and the genome structure were checked by Qualimap (version 2.2.1). BEDTools (version 2.26.0) was used to analyze gene coverage ratio. Quality of the RNA sequencing data was summarized in Table 1.

**TABLE 1:** RNA sequencing data for the eight samples in the four *E. coli* groups cultured in different NaCl concentrations.

Sample	Repeats	Clean reads	Total bases	Mapped reads	Q10*	Q20*	Q30*	GC content
0% NaCl	R1	45850638	6349937612	71.92%	100%	98.83%	95.64%	52.76%
	R2	37508466	5101337699	99.65%	100%	98.74%	95.41%	52.31%
1% NaCl	R1	42539228	5767997978	99.7%	100%	98.81%	95.57%	52.33%
	R2	38463266	5232506247	99.7%	100%	98.78%	95.51%	52.41%
3.5% NaCl	R1	31624808	4431748030	99.53%	100%	98.79%	95.53%	52.82%
	R2	23246030	3208991183	99.31%	100%	98.74%	95.35%	52.64%
5% NaCl	R1	35416518	4824466216	99.36%	100%	98.87%	95.74%	52.75%
	R2	33649560	4647777496	99.01%	100%	98.76%	95.44%	52.82%

*Q10 means the sequencing error rate of the base was less than 10%; Q20 means the sequencing error rate of the base was less than 1%; Q30 means the sequencing error rate of the base was less than 0.1%.

##### Differentially expressed genes

Transcripts were computed by StringTie (version 1.3.3b) in terms of expression levels. Sample distance and difference were analyzed via Principal Component Analysis (PCA) and Principal co-ordinates analysis (PCoA). Transcripts Per Million (TPM) was used for eliminating the influence of gene lengths and sequencing discrepancies, which enabled direct comparison of gene expression levels between samples. Differentially expressed genes (DEGs) between two samples were determined by DESeq2 (version 1.12.4). DEGs were considered as statistically significant if *P*-value was less than 0.05 and log_2_(FoldChange) was greater than 1. For those genes with normalized expression difference equal to zero between two samples, the expression value was adjusted to 0.01 because 0 cannot be plotted on a log plot. If the normalized expression of a certain gene in two libraries was all lower than 1, further differential expression analysis was conducted without this gene. Gene expression differences were visualized by volcano plot and heatmap while sample clusters were generated via principal component analysis (PCA).

##### Network analysis

As for the identification of hub genes, up- and down-regulated genes were analyzed, separately. In specificity, up- or down-regulated genes were input into the String database https://string-db.org/ for building interaction networks (Szklarczyk et al., 2021), respectively. The results were shown in Supplementary Figure 2. These data were then input into CytoScape and visualized for highly-connected proteins via the number of degrees and hub proteins calculated by using the cytoHubba algorithm (Shannon, 2003; Chin et al., 2014).

##### Functional analysis of DEGs

Functional analyses including Gene Ontology (GO) annotation, Kyoto Encyclopedia of Genes and Genomes (KEGG) pathways, and hub genes were performed to identify which DEGs were significantly enriched in GO terms, metabolic pathways, and clusters of genes. Gene Ontology (GO) is a standard classification system for gene functions (Consortium, 2019). For GO annotation, DEGs were mapped to the GO terms (biological process, cellular component, molecular function) in the database. The number of genes in every term was then calculated, and a hypergeometric test is performed to identify significantly enriched GO terms in the gene list out of the background of the reference gene list. KEGG is a public database of pathway data (Kanehisa et al., 2017). KEGG pathway analysis identifies DEGs that are significantly enriched in metabolic pathways or signal transduction pathways compared to a reference gene background via the hypergeometric test. GO terms and KEGG pathway with false discovery rate (*Q*-value < 0.05) were considered as significantly altered.

### Statistical Analysis

A two-tailed unequal variance Student’s *t*-test was calculated for pairwise comparison wherever applicable, unless otherwise instructed. Significant difference is defined as *P*-value less than 0.05. The single-step multiple comparison procedure and statistical test, Tukey’s Honestly Significant Difference (HSD) test, was performed via the R software TukeyC package wherever applicable, which compared the means of every group to the means of every other group simultaneously. Means denoted by a different letter indicated significant differences among groups (*P*-value <0.05).

## Results

### Bacterial Growth

We first tested *E. coli* BW25113 growth pattern on different concentration of glucose and survival ability under different concentrations of NaCl. Both OD_600_ values and the number of viable cells (CFU/mL) were measured, where only the number of viable cells was present while OD_600_ values were present in Supplementary Figure 1. For glucose concentration, 1 × M9 minimal medium supplemented with higher level of glucose (0.8%) showed the highest number of viable cells in the culture, which was significantly different from those with 0.2% and 0.4% glucose (*P*-value <0.05) after culturing for 20 h (Figure 2A). This result is similar with previous studies performed on *E. coli* BL21(DE3) and *E. coli* DH5α, which confirms that high level of carbon source facilitates the replication of bacterial cells (Wang et al., 2018, 2020b). As for osmotic stress, four sodium chloride (NaCl) concentrations (0, 1, 3.5, and 5%) were explored and statistical analysis showed that the number of viable cells in LB broth supplemented with 0% NaCl at 24 h is significantly higher than other tested groups, that is, LB broth supplemented with 1%, 3.5%, and 5% NaCl (Figure 2B). The result is consistent with previous reports that elevated concentration of NaCl could inhibit the growth patterns of bacterial species (Rath et al., 2016; Pang et al., 2020).

### Biofilm Formation, Oxidative Resistance and Motility

**FIGURE 3:**
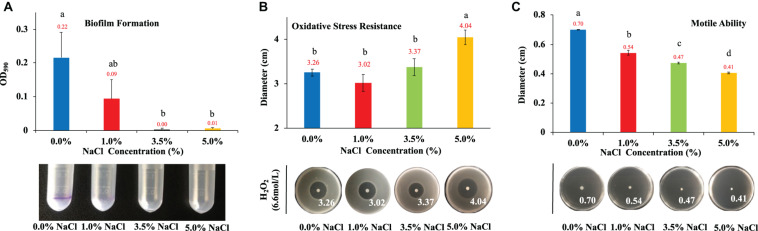
Effects of NaCl on *E. coli* biofilm formation, oxidative stress resistance, and motile ability. **(A)** Effects of different NaCl concentrations on *E. coli* biofilm formation. **(B)** Influences of different NaCl concentrations on *E. coli* oxidative stress resistance. **(C)** Effects of different NaCl concentrations on *E. coli* motile abilities. Tukey’s Honestly Significant Difference (HSD) test was performed, which compared the means of every group to the means of every other group simultaneously. Means denoted by a different letter indicated significant differences among groups (*P*-value <0.05).

Biofilm normally consists of bacterial cells and extracellular polymeric substances (EPS) that helps bacteria to survive in harsh environmental conditions and facilitate host infection (Sharma et al., 2016). Thus, biofilm formation is a survival strategy of bacteria. In this study, we investigated how NaCl concentrations impacted on *E. coli* biofilm formation. According to the results in Figure 3A, NaCl showed a positively dose-dependent inhibition of biofilm growth in *E. coli*, though statistical significance was only detected when comparing *E. coli* in culture of 0% NaCl with that in the culture of 3.5% and 5% NaCl, respectively. In fact, *E. coli* growing in culture with 3.5% and 5% NaCl had negligible amount of biofilm. However, the relationship between hyperosmotic stress and biofilm formation in bacteria is actually controversial. For example, Kawarai et al. (2009) reported that *E. coli* K-12 did not form biofilm in LB broth supplemented with 1 M NaCl, which is consistent with the result in this study, while another study revealed that biofilm formation of *Staphylococcus aureus* was significantly enhanced with the increase of NaCl concentrations (Lee et al., 2014). Thus, how NaCl concentration influences the formation of bacterial biofilm should probably be investigated and explained on a case-by-case basis.

Aerobic bacteria use oxygen to oxidize nutrients for energy or to respire, which leads to the generation of reactive oxygen species (oxidants) for the necessity of various physiological functions. However, if there is an imbalance between the production of oxidants and antioxidants, oxidative stress emerges, which has harmful effects on protein structure and functions with the possibility of causing cell death (Ezraty et al., 2017). Thus, in this study, we tested the roles that NaCl played in combating oxidative stress resistance in *E. coli*. According to the results in Figure 3B, hyperosmolarity induced by 5% NaCl could significantly increase the effects of oxidative damages when compared with other NaCl concentrations (0, 1, and 3.5%). Although we can see that 3.5% NaCl also increases the oxidative stress of H_2_O_2_ on *E. coli* colonies, there is no statistical significance detected when compared with lower NaCl concentrations.

As for motility, it is a common behavior for most bacterial species, through which bacteria are able to reach new environments, looking for niches with high nutrient and low toxins (Mitchell and Kogure, 2006). Some studies suggest that bacterial virulence and motile ability are closely associated via complex regulatory networks (Josenhans and Suerbaum, 2002), while others hold different opinions (Kao et al., 2014). In this study, we explored the effects of NaCl concentrations on the motility of *E. coli* on soft agar plates, the results of which indicated that there was dose-dependent inhibition of bacterial movement with statistical significance among the four NaCl concentrations (Figure 3C).

### Energy Metabolism

In order to grow and replicate in a fast pace, bacteria need to get access to abundant nutrients and absorb sufficient energy from surrounding compounds (Rohmer et al., 2011). In addition, bacterial viability and virulence are also tightly correlated with energy metabolism (Passalacqua et al., 2016). Thus, it is important to understand that how abiotic stress influences bacterial energy metabolism. Glucose is an optimal carbon source for *E. coli* that supports faster growth when compared with other sugars, the dynamic change of which have been explored by a variety of studies (Fuhrer et al., 2005; Yamamotoya et al., 2012). As a widely present carbohydrate reserve in bacteria, glycogen contributes to bacterial survival in the environment under a variety of stresses, such as temperature fluctuation, nutrient deprivation, and osmolarity instability (Wang et al., 2020a). In addition, glycogen also plays important roles in physiological activities such as osmotic regulation and pH maintenance, etc. (Liu et al., 2021). As for trehalose, it is a disaccharide of two glucose molecules linked by α 1,1-glycosidic bond. It is well known that trehalose protects microbes like *Saccharomyces cerevisiae* and *E. coli* from desiccation (Welsh and Herbert, 1999; Tapia et al., 2015). Studies also show that trehalose and trehalose derivatives play important roles in bacterial virulence in terms of host colonization and within-host adaptation (Vanaporn and Titball, 2020) while the deletion of trehalose-synthesizing gene leads to the virulence abolishment of bacterial pathogens (Puttikamonkul et al., 2010; Gebhardt et al., 2015). In this study, we explored the impacts of salt concentrations on the inter-connections of these three important energy compounds.

**FIGURE 4:**
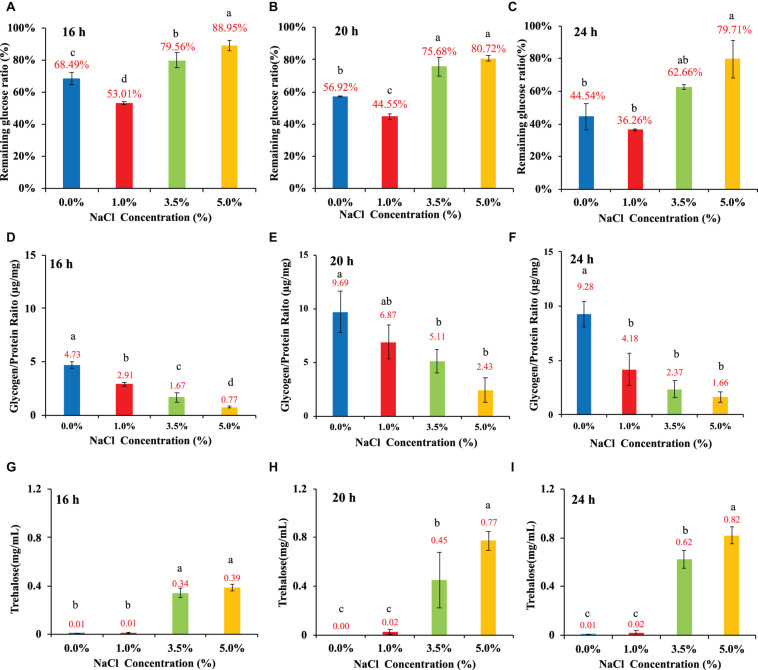
Influences of NaCl concentrations (0, 1, 3.5, and 5%) on *E. coli* glucose consumption rate, glycogen accumulation, and trehalose content at three time points, that is, 16, 20, and 24 h. **(A–C)** Glucose consumption rate. **(D–F**) Glycogen accumulation capacity. **(G–I)** trehalose content. Tukey’s Honestly Significant Difference (HSD) test was performed. Means denoted by a different letter indicated significant differences among groups (*P*-value <0.05).

*E. coli* was cultured in 1 × M9 minimal medium containing 0.8% glucose and different concentrations of NaCl (0, 1, 3.5, and 5%). The general changing patterns of glucose consumption rates at the three time points (16, 20, and 24 h) were the same, which indicated that the effects of NaCl concentrations on *E. coli* glucose uptake from the culture were consistent. In specificity, *E. coli* growing in 1% NaCl had the highest glucose uptake value while 3.5% and 5% NaCl inhibited the absorption of external glucose with statistical significance at both 16-h and 20-h (Figures 4A–C). At 24-h, 5% NaCl had the lowest level of glucose absorption than cultures containing other concentrations of NaCl (Figure 4C). As for glycogen metabolism, it was obvious that NaCl had a dose-dependent inhibitory effect on glycogen accumulation in *E. coli* (Figures 4D–F). In addition, glycogen accumulation also showed a time-dependent pattern when the culture was the same, that is, higher at 20-h and lower at 16- and 24-h, which was also consistent with previous studies (Wang et al., 2020b). Moreover, trehalose accumulation in cultures with different NaCl concentrations also showed dose-dependent changing patterns (Figures 4G–I). However, in contrast to glycogen accumulation, NaCl facilitated the accumulation of trehalose in *E. coli* BW25113 in response to hyperosmotic stress. According to the statistical analysis, *E. coli* cultured at 3.5% and 5% NaCl conditions accumulated significantly more trehalose than bacteria cultured at 0% and 1% NaCl, which was independent of time points. It was also obvious that *E. coli* accumulated the highest amount of trehalose at 24-h (Figure 4I). Thus, trehalose accumulation could play important roles in *E. coli* response to hyperosmotic stress as previously confirmed (Dinnbier et al., 1988; Purvis et al., 2005).

### Effects of NaCl Concentrations on *Escherichia coli* Δ*otsA* and Δ*otsB*

Previous study showed that trehalose content was significantly enhanced in wild-type *E. coli* under high salinity concentration (Figures 4G–I). In bacteria, three trehalose synthesis pathways have been reported, which are OtsAB, TreYZ and TreS (Avonce et al., 2006), among which the pathway consisting of OtsA (trehalose-6-phosphate synthase) and OtsB (trehalose-6-phosphate phosphatase) uses UTP-glucose as a substrate, which is essential for trehalose synthesis in some bacteria such as *Mycobacterium tuberculosis* (Murphy et al., 2005). As for TreYZ pathway, TreY (maltooligosyl-trehalose synthase) and TreZ (maltooligosyltrehalose trehalohydrolase) work together to convert maltodextrin to maltooligosyltrehalose and then hydrolyze this product to form free trehalose (Elbein, 2003). TreS is trehalose synthase that inter-convert trehalose (α-D-1,1-glucose) to maltose (α-D-1,4-glucose) (De Smet et al., 2000). Since OtsAB pathway is directly linked with glucose for trehalose synthesis while the other two pathways used maltodextrin and maltose, we focused on single-gene inactivation of the OtsAB pathway in terms of their responses to the fluctuations of NaCl concentrations in this study.

A variety of studies have explored how the inactivation of *otsA* and/or *otsB* influenced bacterial physiology in different bacterial specie under different conditions. For example, a recent study by Hubloher et al. showed that deletion of *otsB* in *Acinetobacter baumannii* led to growth impairments at high salt conditions (Hubloher et al., 2020). Another study of *E. coli ΔotsA* showed that trehalose played a protective role in stabilizing outer membrane when *E. coli* was under abiotic stress (Chang et al., 2019). In addition, Algara et al. revealed that trehalose protected *E. coli* against carbon stress because *E. coli ΔotsA*, a trehalose-abolished strain, showed increased Nε-lysine acetylation and protein aggregation (Moruno Algara et al., 2019). Although this study showed that high salt condition facilitated trehalose accumulation and inhibited glucose uptake and glycogen accumulation in wild-type *E. coli*, whether the disruption of trehalose synthesis could lead to any different physiological changes when compared with the wide-type strain in response to hyperosmotic stress is unknown.

**FIGURE 5:**
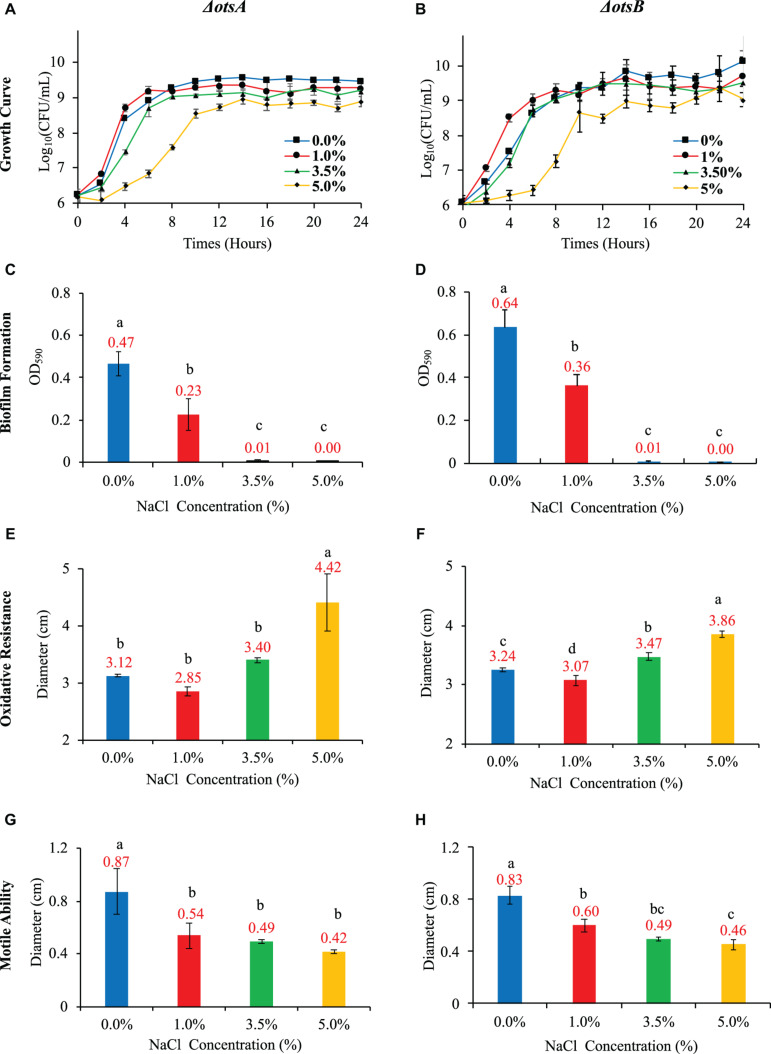
Effects of NaCl on growth rate, biofilm formation, oxidative resistance, and motile ability in *E. coli ΔotsA* and Δ*otsB*. **(A,B)** Effects of different NaCl concentrations on the growth rates of *E. coli* mutants. **(C,D)** Effects of different NaCl concentrations on *E. coli* mutant biofilm formation. **(E,F)** Influences of different NaCl concentrations on the oxidative stress resistance of *E. coli* mutants. **(G,H)** Effects of different NaCl concentrations on *E. coli* mutant motile abilities. Tukey’s Honestly Significant Difference (HSD) test was performed, which compared the means of every group to the means of every other group simultaneously. Means denoted by a different letter indicated significant differences among groups (*P*-value <0.05).

In this study, we compared the two single-gene inactivated *E. coli* BW25113 mutants Δ*otsA* and Δ*otsB* in terms of their growth pattern, biofilm formation, oxidative stress resistance, and motile abilities with the wild-type strain. Both *E. coli* mutants showed abolishment of trehalose accumulation at 20 h (Supplementary Figure 3), which indicated that inactivation of Δ*otsA* and Δ*otsB* led to the disruption of trehalose synthesis. In addition, both mutants showed analogous growth patterns as wild-type *E. coli* strain when no NaCl was supplemented in the LB broth while increased NaCl concentration inhibited the general growth trends of the two mutants in a dose-dependent manner that is similar to wild-type strain, though *E. coli ΔotsB* seemed to have extended lag phase (Figures 5A,B). As for biofilm formation, the capacity of *E. coli ΔotsB* was significantly higher than *E. coli ΔotsA* at both 0% and 1% NaCl while both mutants showed significantly increased biofilm formation than wild-type strain at 0% and 1% NaCl. When NaCl concentrations increased to 3.5 and 5%, no biofilm could be detected in both mutant and wild-type strains (Figures 5C,D). As for oxidative resistance and mobile ability, both mutants showed similar patterns as the wild-type strain with no statistical significance (Figures 5E–H). In sum, physiological analysis showed that inactivation of trehalose synthesis pathway in *E. coli* BW25113 mainly impacted on bacterial biofilm formation while other features were not significatnly influenced.

**FIGURE 6:**
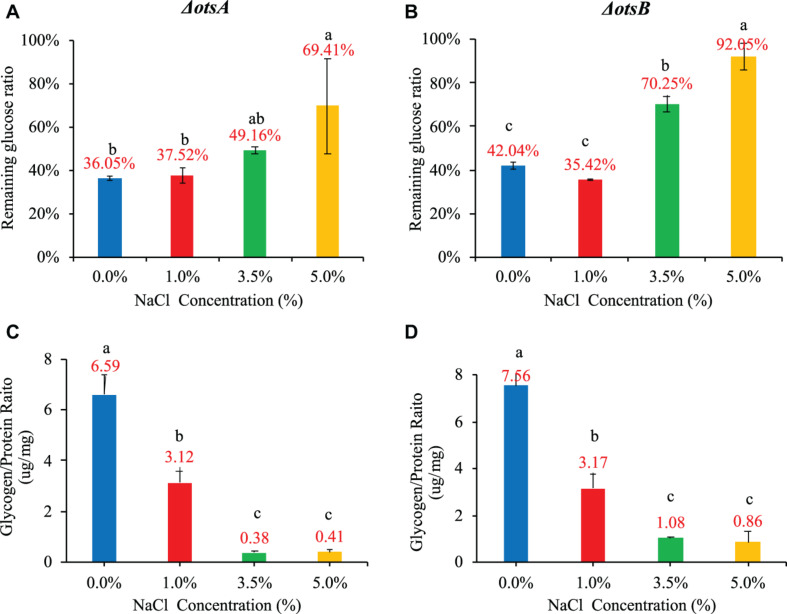
Influences of NaCl concentrations (0, 1, 3.5, and 5%) on *E. coli ΔotsA* and Δ*otsB* in terms of glucose consumption rate and glycogen accumulation at 20 h. **(A,B)** Glucose consumption rates. **(C,D)** Glycogen accumulation capacity. Tukey’s Honestly Significant Difference (HSD) test was performed. Means denoted by a different letter indicated significant differences among groups (*P*-value <0.05).

As for glucose consumption in the 20-h culture, the two mutants showed very different abilities with each other and also when compared with wild-type strain. In particular, glucose uptake of strain Δ*otsA* decreased proportionally with the increase of NaCl concentration; in contrast, strain Δ*otsB* showed the highest glucose uptake at 1% NaCl, which was similar as the wild-type strain. In addition, glucose consumption ability of strain Δ*otsA* was significantly higher than both wild-type and Δ*otsB* strains at the concentrations of 3.5% and 5% NaCl while wild-type and Δ*otsB* strains showed a similar changing glucose uptake pattern (Figures 6A,B). As for glycogen accumulation, the two trehalose-deficient mutants showed similar NaCl-concentration-dependent trends as the wild-type strain, though strain Δ*otsB* seemed to have higher amount of accumulated glycogen than strain Δ*otsA* at each NaCl concentration (Figures 6C,D). However, the accumulated glycogen contents in the two mutated strains were significantly reduced when compared with wild-type strain, especially at higher NaCl concentrations.

### Transcriptome Analysis

#### Basic Info of RNA Sequencing

In order to understand bacterial physiological changes in response to salt stress at gene transcriptional level, we performed transcriptomic study on *E. coli* culture at four NaCl concentrations (0, 1, 3.5, and 5%). cDNA libraries prepared from 8 samples of *E. coli* were sequenced on the HiSeq XTen sequencers platform. A total of 297,780,562 raw reads were generated. After the sequencing adapters and low-complexity reads were removed, 288,298,514 clean reads were generated with an average of 41679552, 40501247, 27435419, and 34533039 reads in the cDNA libraries of *E. coli* cultured in 0%, 1%, 3.5%, and 5% NaCl concentrations, respectively. The clean reads which were perfectly mapped to the reference genomes without mismatch were all greater than 70% while reads with sequencing errors less than 1% in all samples are more than 98%. A detailed summary of the sequencing results is shown in Table 1.

#### Differentially Expressed Genes

In order to better elucidate the phenotype changes of the wild-type *E. coli* under salt stress, we first divided *E. coli* cultured in the four concentrations of NaCl into two groups, high salinity group (3.5% and 5% NaCl) and low salinity group (0 and 1%). We then investigated the differentially expressed genes (DEGs) of *E. coli* cultured in high and low salinity conditions by Illumina high-throughput RNA sequencing, respectively. Using genome-wide transcriptional analysis, a total of 1,117 genes (653 upregulated and 464 downregulated genes) were found to be differentially expressed by the criteria of log_2_(Fold_Change) > 1 and *P*-value <0.05. All the up- and down-regulated genes, together with basic information such as gene names, UniProt IDs, fold changes and P-values, could be found in Supplementary Table 1. The top 20 genes in terms of fold changes in the up- and down-regulated gene sets in this study were present in Supplementary Table 2, respectively. In particular, for the up-regulated genes, *stpA* (log2|Fold_Change| = 8.09, *P*-value = 2.23E-57) has the highest differential expression level with significant difference, which encode a DNA-binding protein. In contrast, among the down-regulated genes, *glrK* (log2|Fold_Change| = −4.50, *P*-value = 5.98E-134) shows the highest differential expression level. It encodes a sensor protein that plays important roles in sensing histidine kinase.

#### Network Analysis

Considering the large number of up- and down-regulated genes caused by hyper-saline pressure in the *E. coli* transcriptome, we performed two types of network analyses in order to identify the hub genes (proteins). The first analysis relies on the functional protein association network automatically constructed by STRING database, which was then imported into CytoScape for visualization. In a network, some nodes (proteins) are connected with one or more nodes while other nodes are independent; if a node is highly connected within a network., then the node is considered as a potential hub with important functions (Berlingerio et al., 2011). Based on the up- and down-regulated gene networks constructed by STRING (Supplementary Figure 2), we ranked the genes according to their degrees, that is, the number of connected nodes in descending order. Top 20 genes in each network were collected and annotated via UniProt database (Consortium, 2015) (Supplementary Table 3). According to the result, *ppsA* (log2|Fold_Change| = 1.28, *P*-value = 2.85E-8) has the highest degree (*n* = 58) among the up-regulated genes, which encodes phosphoenolpyruvate synthase (PEP synthase) in *E. coli*. Meanwhile, *metG* (log2|Fold_Change| = −1.68, *P*-value = 1.98E-29) is the most connected hub (*n* = 56) among all the down-regulated genes, which encodes methionine-tRNA ligase in *E. coli*.

**FIGURE 7:**
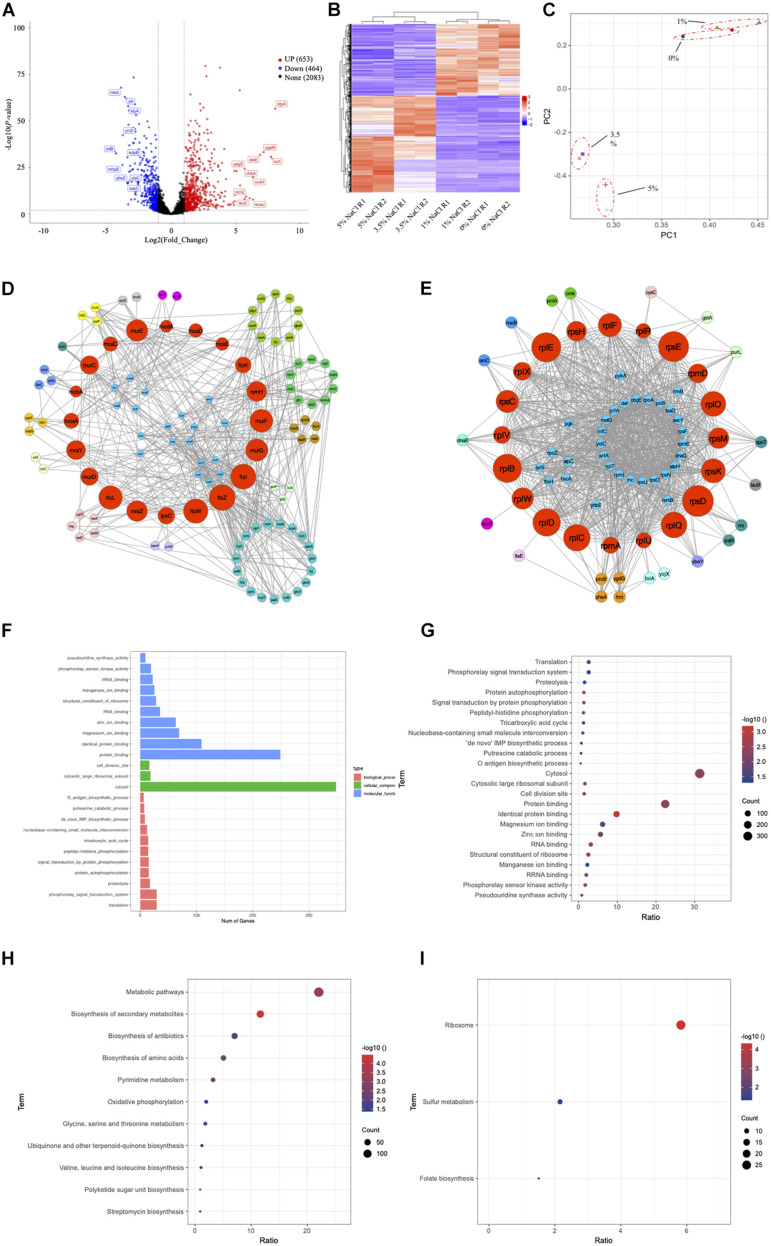
Transcriptomic study of *E. coli* BW25113 under four NaCl concentrations of 0, 1, 3.5, and 5%. Since the phenotypes of *E. coli* under 0% and 1% NaCl are more consistent, while the phenotypes of *E. coli* under 3.5% and 5% NaCl are more similar, we combine 0% and 1% NaCl samples together as Group 1 and 3.5% and 5% NaCl samples together as Group 2 for the analysis. **(A)** Volcano plot for DEGs. **(B)** Heatmap plot for DEGs. **(C)** PCA analysis for group clusters. **(D)** Hub nodes among up-regulated genes. **(E)** Hub nodes among down-regulated genes. **(F)** Bar plot of GO term enrichment analysis. **(G)** Bubble plot of GO term enrichment analysis. **(H)** KEGG pathway enrichment for up-regulated genes. **(I)** KEGG pathway enrichment for down-regulated genes.

However, topology of PPI network is rather complex and node degree is just one of the features. Thus, in order to make sure that our study predicts essential proteins with better performance and high precision, we used the cytoHubba plug-in in the CytoScape software by choosing the recommended algorithm Maximal Clique Centrality (MMC) (Chin et al., 2014). According to the up-regulated gene network in Supplementary Table 4, the top 20 hub genes are mainly responsible for cell division and cell wall formation (*ftsZ*, *ftsI*, *ftsW*, *ftsL*, *murE*, *murG*, *murF*, *mraZ*, *mraY*, *murC*, *murD*, *lpxC*), which belong to the so-called “division and cell wall (dcw) cluster” in *E. coli* (Figure 7D). In addition, ribosomal RNA small subunit methyltransferase H (*rsmH*), DNA translocase (*ftsK*), and molybdenum/molybdate related proteins (*moaA*, *moaE*, *moaD*, *moaC*, *modA*, *mobA*) are also recognized. As for the down-regulated gene network, the top 20 hub genes all encode ribosomal protein subunits (Figure 7E), which include *rpsD*, *rpsE*, *rplE*,*rplB*, *rplD*, *rplC*, *rplQ*, *rplO*, *rpsK*, *rplF*, *rpsC*, *rplW*, *rpsM*, *rpsH*, *rpmD*, *rplX*, *rplV*, *rpmA*, *rplU* and *rplR* (Supplementary Table 4).

#### GO Enrichment Analysis of DEGs

GO annotation system is an international standard for gene function classification, which includes biological process (BP), cellular component (CC), and molecular function (MF). In order to understand the biological roles of the most significantly up- or down-regulated genes, GO enrichment analysis was performed by using the Fisher’s Exact Test with *P*-value <0.05 as the threshold. In specificity, the up-regulated DEGs were enriched to 24 GO terms and classified into categories of BP with 11 GO terms, CC with 3 GO terms, and MF with 10 GO terms. As shown in Figures 7F,G, the top three enriched terms in BP were “translation (GO:0006412),” “phosphorelay signal transduction system (GO:0000160),” and “proteolysis (GO:0006508),” with the number of DEGs being 29, 29, and 17, respectively. In the CC category, all three enriched terms were “cytosol (GO:0005829),” “cytosolic large ribosomal subunit (GO:0022625),” and “cell division site (GO:0032153)” with the number of DEGs being 348, 18, and 16. In the category of MF, genes were significantly enriched into the top three terms of “protein binding (GO:0005515),” “identical protein binding (GO:0042802),” and “magnesium ion binding (GO:0000287),” and the number of DEGs being 249, 109, and 69, respectively. More information about the involved genes of GO enrichment is shown in Supplementary Table 5.

#### KEGG Pathway Analysis of DEGs

KEGG pathway was used for biological pathway analysis, in which up- and down-regulated genes were mapped to the reference pathways of KEGG. For the up-regulated genes, the significantly enriched top six pathways (*P*-value < 0.05) were “metabolic pathways (eco01100, *n* = 144),” “biosynthesis of secondary metabolites (eco01110, *n* = 76)” and “biosynthesis of antibiotics (eco01130, *n* = 46),” “biosynthesis of amino acids (eco01230, *n* = 33),” “pyrimidine metabolism (eco00240, *n* = 21)” and “oxidative phosphorylation (eco00190, *n* = 13)” (Figure 7H). As for the down-regulated genes, all three significantly enriched pathways were “ribosome (eco03010, *n* = 27),” “sulfur metabolism (eco00920, *n* = 10),” and “folate biosynthesis (eco00790, *n* = 7)” (Figure 7I). More information about the results of KEGG enrichment is presented in Supplementary Table 6.

## Discussion

Environmentally-transmitted pathogenic bacteria frequently encounter a variety of abiotic stresses, such as temperature, oxidation, pH and osmosis, et al. (Wang et al., 2017). These stresses impose selection pressure on bacterial environmental durability and virulence with immediate effects at shorter time scale and evolutionary effects at longer time scale (Brouwer et al., 2019). So far, many studies have explored the effects of abiotic stresses on bacterial phenotypes (Boor, 2006; Mihaljevic et al., 2007; Pinto et al., 2014; Ezraty et al., 2017; Guan et al., 2017). However, few studies tried to explore stress responses by linking bacterial environmental durability and virulence. For example, it was noted that high level of salinity led to the expression of genes related with potential virulence factors, phage capsid proteins, and drug resistance proteins in *Burkholderia pseudomallei*, which indicated the potential positive linkages between bacterial stress adaptation and virulence factors (Dai et al., 2015). Dayma et al. also noticed that low salinity stress facilitated the virulence potential and biofilm formation of marine bacterium *Vibrio alginolyticus* (Dayma et al., 2016). In this study, we investigated the potential influences of salt stress on wild-type *E. coli* and two trehalose-deficient mutants in terms of growth pattern, phenotypes associated with pathogenic virulence, and energy metabolism, from which we tried to understand the short-term effects of this particular environmental stress on bacterial durability and virulence. After that, in order to understand the potential molecular mechanisms of *E. coli* adaptation to different concentrations of NaCl, we performed a comparative transcriptome study. DEGs and significantly enriched pathways were identified for a better understanding of the molecular mechanisms of bacterial responses to high salinity stress. Among the DEGs, potential virulence genes that were up-regulated in high salinity conditions were discussed, which could contribute to the elucidation of the potential linkages between bacterial environmental adaptation and the evolution of virulence.

Nutrient fluctuation is a commonly encountered abiotic stress for bacteria during their transmission between hosts and external environment (Wang et al., 2020b). Its driving force will change bacteria at both physiological and genetic level. For example, Ratib et al. studied *E. coli* in long-term stationary phase (LTSP) for over 1,200 days, which revealed that two physically distinct lineages evolved from the culture (Ratib et al., 2021). Tenaillon et al. studied the evolution of *E. coli* for 2000 generations at high temperature, finding two distinct adaptive pathways (Tenaillon et al., 2012). As for short-term adaptation, Biselli et al. discovered that *E. coli* tended to decrease growth and maintenance rate in exchange of longer survival during carbon deficiency (Biselli et al., 2020). In this study, we examined the response of *E. coli* BW25113 to nutrient fluctuation, according to which, higher glucose concentration (0.8%) significantly improved the number of viable cells during 24-h culture (Figure 1A), which is consistent with previous studies (Wang et al., 2018, 2020b). Bacterial replication is a key factor for host colonization, resource exploitation, and between-host transmission (Rohmer et al., 2011), high rate of which could contribute to bacterial virulence. A study using the environmentally transmitted bacterial fish pathogen *Flavobacterium columnare* as a model organisms revealed that abundant nutrients in environment could act as a selective pressure for higher virulence and faster evolutionary rate (Kinnula et al., 2017). On the other hand, nutrient deficiency has been reported to diversify population structure and virulence strategies in opportunistic pathogens (Sundberg et al., 2014). Further studies showed that osmotic stress had negative influences on *E. coli* growth. Although *E. coli* requires NaCl to grow, high concentrations of NaCl significantly inhibited the growth rate of *E. coli* (Figure 1B). In fact, *E. coli* has the optimal concentration of NaCl at 0.5% (w/v) for growth (Abdulkarim et al., 2009) while it could gradually adapt to 11% NaCl, though it is not a halophilic bacterium (How et al., 2013).

As for biofilm, it is a bio-matrix of extracellular polymeric substances (EPSs) that consist of exopolysaccharides, fibril proteins, and DNA (Bremer and Krämer, 2019). In this study, NaCl concentrations showed a dose-dependent inhibitory effect on *E. coli* (Figure 3A). In particular, *E. coli* growing in the culture media supplemented with 0% and 1% NaCl had detectable amount of biofilm while 3.5% and 5% NaCl completely inhibited biofilm formation. Previously, studies had already showed that osmotic stress could induce biofilm production in a variety of microbes such as *Staphylococcus epidermidis*, *Clostridium ljungdahlii* and *Candida albicans* (Pemmaraju et al., 2016; Yang et al., 2017; Ferreira et al., 2019). Lee et al. (2014) used quantitative real-time PCR to analyze the expression levels of biofilm-related genes, which included *icaA*, *atl*, *clfA*, *fnbA*, *sarA* and *rbf*, which revealed that elevated NaCl concentration increased the formation of biofilm in *S. aureus* via the up-regulation of *icaA*. Another study by Lim et al. (2004) showed that biofilm formation in *S. aureus* was multi-phasic; although increased NaCl concentration facilitated the formation of biofilm, the process was actually controlled by different genes at different stages, while no biofilm formation could be detected when NaCl concentration was higher than 5.6%. Thus, the results are not consistent in terms of effects of NaCl concentrations on biofilm formation among bacterial species. In sum, it is worthy of further investigation into the inhibitory effects of NaCl on *E. coli* biofilm formation at molecular level via transcriptomic analysis.

In terms of oxidative stress, previous studies showed that increased NaCl concentration could enhance bacterial oxidative resistance via chloride and sodium ion channels rather than degradation of hydrogen peroxide in the bacterial pathogen *Burkhoderia pseudomallei* (Pumirat et al., 2017). In a recent study conducted by Hassan et al., it was revealed that salinity stress was able to enhance the antioxidant capacity of *Bacillus* and *Planococcus* species isolated from saline lake environment; however, the two species showed strain-specific responses in terms of oxidative resistance (Hassan et al., 2020). For *Planococcus*, high-salinity-induced resistant strains increased glutathione cycle, phenols, flavonoids, antioxidant capacity, catalase, and/or superoxide dismutase while *Bacillus* species used phenols, flavonoids, peroxidase, glutaredoxin, and/or SOD to combat oxidative stress (Hassan et al., 2020). In addition, studies also inter-linked oxidative stress with bacterial virulence, which showed that the deletion of YqhG gene encoding periplasmic protein in uropathogenic *E. coli* not only decreased bacterial virulence but also increased sensitivity to oxidative stress (Bessaiah et al., 2019). In this study, as for the effects of NaCl concentrations on *E. coli* adaptive response to oxidation, it was found that high salt condition actually reduced oxidative resistance (Figure 3B). In *E. coli*, some enzymes related with oxidative stress resistance were previously reported, which included but not limited to OxyR, SoxRS, and RpoS (Chiang and Schellhorn, 2012). However, how alteration of NaCl concentrations in the culture medium influenced the expression of these genes have not been clearly elucidated yet, which requires further investigation.

Our study also identified that enhanced NaCl level could significantly reduce bacterial motile abilities in a dose-dependent manner (Figure 3C). It was previously reported that the fish pathogen *Edwardsiella tarda* moved much faster in low salt conditions than in high salt conditions (Yu et al., 2011) while Ikeda et al. (2020) showed that the hypoosmotic stress induces flagellar biosynthesis and swimming motility in the food pathogen *Escherichia albertii*. In a genome-wide screening analysis, Inoue et al. used Keio collection, a set of single-gene knockout mutants of all the nonessential genes in *E. col*i K-12, to find all the motility-related genes in *E. coli*, according to which 78 genes were strongly related with *E. coli* motile ability (Inoue et al., 2007). However, how these genes were influenced by differential NaCl concentrations is worthy of further investigations.

Glycogen and trehalose have been widely investigated due to their roles in osmoprotection (Seibold et al., 2007; Wang et al., 2020a). In addition, the metabolic pathways of the two compounds, together with glucose and maltose, were also inter-connected (Pan et al., 2008). However, how salt stress influences the inter-conversion process of these carbohydrates is not clear. Thus, in this study, we investigated the potential correlations among glucose consumption rate, glycogen accumulation, and trehalose content under different NaCl concentrations (Figure 4). For glucose uptake rate, the results showed that high salinity significantly inhibited the uptake of glucose in the culture medium (Figures 4A–C). However, by checking the expression levels of the five regulatory genes (EI:*pstI*, HPr:*pstH*, EIIA^*Glucose*^:*crr*, EIICB^*Glucos*^e:*ptsG*, and AC:*cyaA*) for glucose uptake via the phosphoenolpyruvate phosphotransferase system, no significant up- or down-regulations were identified (Steinsiek and Bettenbrock, 2012). Thus, the molecular mechanisms behind the glucose uptake inhibition should be further investigated. Meanwhile, both glycogen and trehalose contents showed dose-dependent relationship with NaCl concentrations. The difference is that increased NaCl concentration inhibits glycogen accumulation but facilitates the build-up of trehalose. It is well documented that storing around 55,000 glucose molecules as a single glycogen particle as an energy reserve has little effect on bacterial internal osmotic pressure (Wilson et al., 2010). In fact, halophilic archaea, when compared with thermophilic archaea, do not even have complete glycogen metabolism pathways based on a large number of genomic analysis (Wang et al., 2019). In contrast, many studies have reported that trehalose accumulation provides strong protection for bacteria under osmotic stress via a variety of pathways, such as glycogen degradation and trehalose synthesis, etc. (Welsh and Herbert, 1999; Pan et al., 2008; Tapia et al., 2015). However, in this study, we neither observed significantly enhanced gene levels for glycogen degradation nor noticed significantly improved levels of trehalose synthesis genes, which suggested that there might be missing links between glycogen and trehalose metabolism under high salt stress.

Previous studies have already reported the protection mechanism of trehalose in *E. coli* under low temperature stress (Kandror et al., 2002) while enhanced trehalose production improves growth of *E. coli* under osmotic stress (Purvis et al., 2005). In order to understand the roles of trehalose in *E. coli* growing in different NaCl concentrations, we explored two *E. coli* strains with trehalose synthesis deficiency, that is, *E. coli ΔotsA* and Δ*otsB*. Our study confirmed that growth rate of the two mutants decreased smoothly with increasing NaCl (Figures 5A,B). However, no significant difference was identified between mutated strains and the wild-type strain in terms of growth rate at the same NaCl concentration. Thus, there should be other metabolites facilitating the survival of *E. coli* at high salt level. The impacts of high salinity on biofilm formation (Figures 5C,D), oxidative resistance (Figure 5E,F), and motile ability (Figures 5G,H) of the two mutants were also similar to wild-type *E. coli* in terms of the general trend. However, trehalose deficiency seems to facilitate biofilm formation under low salinity condition (0 and 1%) for both *E. coli ΔotsA* and Δ*otsB* when compared with wide-type strain, which is worthy of further investigation. As for glucose consumption and glycogen accumulation, *E. coli ΔotsA* showed better glucose metabolism ability than *E. coli ΔotsB*, especially at high NaCl concentrations (3.5 and 5%) while glycogen accumulation abilities of both mutated strains were significantly inhibited with no clear explanation (Figure 6). Thus, how the trehalose and glycogen metabolism pathways are inter-twined under salt stress should be further explored at the levels of transcriptome, proteome, and metabolome.

Further transcriptomic analysis provided an overview of how *E. coli* BW25113 responded to the stimulation of high salinity stress (Figure 7). Volcano plot, heatmap, and PCA clusters confirmed that *E. coli* responded to high salinity stimulus systematically at molecular level. The up- and down-regulated gene sets provide highly valuable info for further molecular studies of *E. coli* adaptation to high salinity condition. In terms of bacterial growth rate, we first checked the expression level of *dnaA*, the gene encoding the initiator protein of chromosome replication (Sekimizu et al., 1987); however, no significant change was identified in low and high salt concentrations. As for biofilm, a group of genes were previously reported to affect biofilm formation and architecture upon over-expression (Tenorio et al., 2003), among which ten genes was found to reduce biofilm formation when over-expressed while only gene *secY* (secretion pathway component) was present in the list of DEGs in this study. However, our study showed that the expression level of *secY* was actually suppressed when biofilm formation was abolished at high NaCl concentration (Supplementary Table 1). Thus, the two results showed inconsistency, which requires further exploration. As for the abnormality of biofilm architecture, we noticed that 4 out of 36 genes were present in our DEGs list (Tenorio et al., 2003), among which *evgS* (FC = 5.91), *wcaJ* (FC = 6.37) and *yfcS* (FC = 1.18) were significantly over-expressed, indicating the formation of abnormal biofilm at high NaCl concentrations. Studies also revealed that three transcription factors, OxyR (hydrogen peroxide-inducible genes activator), SoxR (Redox-sensitive transcriptional activator SoxR), and SoxS (Regulatory protein SoxS), play critical roles in the regulation of oxidative stress resistance in bacteria (Seo et al., 2015). Both OxyR and SoxR were not identified as differential expressed genes in this study while SoxS was significantly up-regulated to 2.02 folds, which links SoxS with the salt-induced oxidative stress susceptibility and requires further exploration. Genome-wide screening of motility related genes via Keio collection in *E. coli* found 78 genes, the mutation of which could cause strongly repressed swarming and swimming (Inoue et al., 2007). Through searching the 78 genes in the list of DEGs in this study, we identified 23 genes that were significantly expressed due to high salt level, among which 13 genes were significantly down-regulated (Supplementary Table 7). It should be highlighted that the gene *tolB* encoding the Tol-Pal system protein TolB was significantly down-regulated by 4.29 folds, which ranked the second of all down-regulated genes by salt stress (Supplementary Table 1). In addition to its close association with *E. coli* motility (Inoue et al., 2007), it was also reported that Tol-Pal system contributed to the enteric pathogenesis of enterohemorrhagic *E. coli* (EHEC) associated with the Type III secretion system (T3SS) (Hirakawa et al., 2020). Thus, high NaCl concentration potentially had synergistic effects on inhibiting both motility and virulence in *E. coli*.

Moreover, the highest up-regulated gene *stpA* encodes a DNA-bind protein, which condenses supercoiled DNA in preference to linear DNA and protects bacterial DNA from digestion under stress conditions (Keatch, 2005). The specific molecular mechanism of the process has not been studied under osmotic stress, which could provide novel insights into bacterial survival strategies. As for other differentially expressed genes and functionally-important hub genes, similar analyses should be performed in order to understand *E. coli* responses to osmotic stress in details. It was also worthy of mentioning that both STRING and cytoHubba analyses revealed that most of the down-regulated hub genes belonged to ribosomal proteins, which indicated that the translation initiation was generally inhibited during osmotic stress (Uesono and Toh-E, 2002). As for the GO term enrichment, it is obvious to see that most of the DEGs fall into the cellular component term cytosol (GO:0005829), which reflects that *E. coli* responds to osmotic stress mainly via physiological activities internally. As for the KEGG pathways, up-regulated genes are mainly enriched in the “Metabolic pathway” and “Biosynthesis of secondary metabolites” while down-regulated genes in “Ribosome” pathway, which indicates that *E. coli* responds to osmotic stress via metabolic change and also a variety of secondary metabolites, rather than synthesis of novel proteins. Further metabolomic and proteomic studies could provide more details in terms of the molecular mechanisms. As for potential virulence factors in *E. coli*, such as lipopolysaccharides and fimbria, the results showed inconsistent up- and down-regulation patterns in response to osmotic stress. For example, the expression levels of fimbria-relavant genes such as *ecpB*, *yfcR*, *yfcS* and *yehA* were significantly up-regulated while *ycfP*, *yadV*, *sfmH*, *yehC*, *yehD*, *yadK* and *sfmF* were significantly down-regulated. Such inconsistency might explain the diversification of virulent phenotypes of the fish pathogens during long-term starvation survival in the environment (Sundberg et al., 2014) since the evolution of virulence is a sophisticated but delicated process that requires the balance of many molecular processes.

## Conclusion

In this study, we investigated the influences of NaCl concentrations on growth patterns, phenotypes associated with virulence, and energy metabolism in wild-type *Escherichia coli* BW25113 and its two single-gene mutants Δ*otsA* and Δ*otsB*. The results indicated that elevated NaCl concentrations in the culture medium generally inhibited bacterial growth, biofilm formation, oxidative resistance, and motile ability in both wild-type strain and trehalose-deficient mutants. As for energy metabolism, it was confirmed that trehalose was preferred under hyper-saline conditions than glycogen in *E. coli* BW25113, while glucose uptake was significantly inhibited at higher NaCl levels. As for the *E. coli ΔotsA* and Δ*otsB*, trehalose was completely abolished in the two mutated strains. However, when compared *E. coli* wild-type strain with its two mutants, it was shown that inactivation of trehalose synthesis pathway facilitated biofilm formation at low salinity level. Further transcriptomic analysis revealed the significantly up- and down-regulated genes that were responsible for *E. coli* responses in high salinity condition. Hub genes and enriched pathways were also identified for general understanding of *E. coli* survival strategy under osmotic pressure. Although representative phenotypes associated with pathogenic characteristics in *E. coli* were negatively impacted by hyperosmotic pressure in this study, transcriptomic analysis revealed that gene expressions related with salt response and virulence were actually inter-connected. Thus, this study does not necessarily disapprove the sit-and-wait hypothesis since evolution involves long-term interactions with and adaptations to environment. Further studies may need to examine the phenotype changes of *E. coli* after long-term adaptation to hyperosmotic conditions, investigating specific pathways and gene clusters by experimental methodologies at molecular level for a better understanding of *E. coli* adaptations to abiotic stresses.

## Data Availability Statement

The datasets presented in this study can be found in online repositories. The names of the repository/repositories and accession number(s) can be found in the article/Supplementary Material.

## Author Contributions

BG and LW conceived and designed the experiments. LW, BG, and Q-HL contributed to project administration. FL, X-SX, Y-YY, J-JW, M-MW, and Q-HL carried out the experimental work. LW and J-WT performed the transcriptome analysis. LW, QH-L, FL, X-SX, M-MW, and J-WT wrote and revised the manuscript. LW and Q-HL provided platform and resources. All authors read and approved the final manuscript.

## Conflict of Interest

The authors declare that the research was conducted in the absence of any commercial or financial relationships that could be construed as a potential conflict of interest.

## Publisher’s Note

All claims expressed in this article are solely those of the authors and do not necessarily represent those of their affiliated organizations, or those of the publisher, the editors and the reviewers. Any product that may be evaluated in this article, or claim that may be made by its manufacturer, is not guaranteed or endorsed by the publisher.
