# Supplementary material for: Effects of NaCl Concentrations on Growth Patterns, Phenotypes Associated With Virulence, and Energy Metabolism in Escherichia coli BW25113
Source: Front Microbiol. 2021 Aug 16;12:705326. doi: 10.3389/fmicb.2021.705326 (PMC8415458; doi:10.3389/fmicb.2021.705326)
Supplement: Supplementary file 1 [file Image_1.pdf]

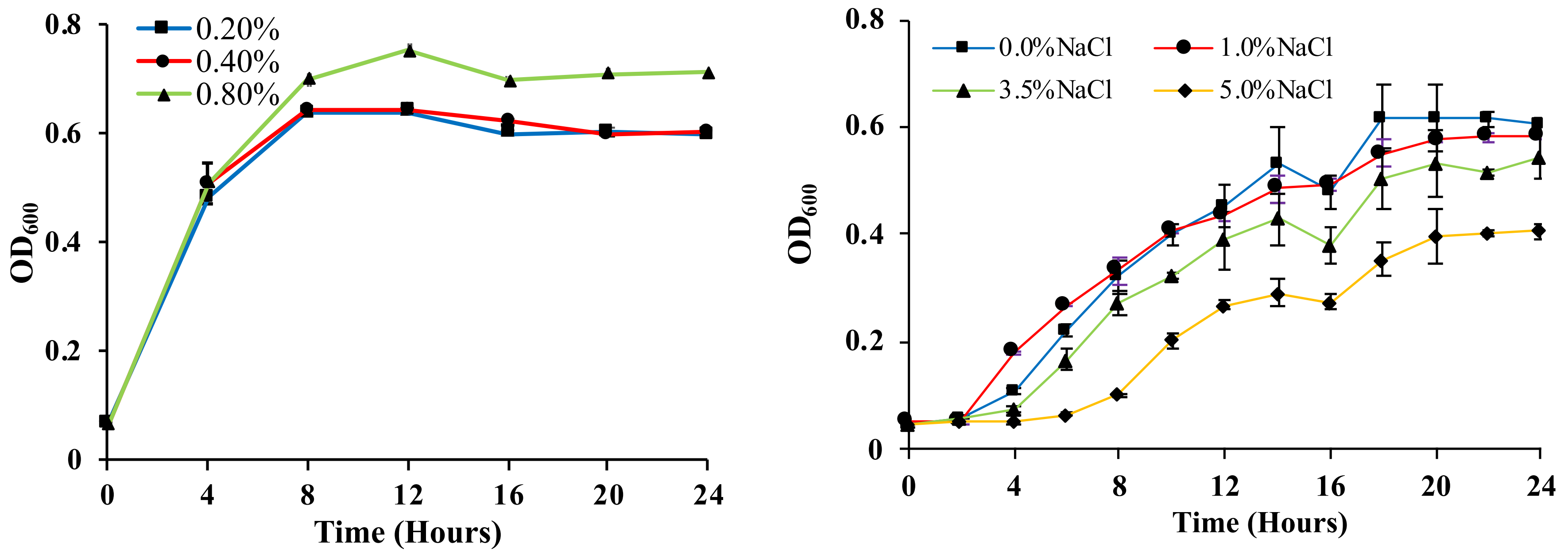

Supplementary Figure 1 Effects of glucose and NaCl concentrations on the growth rates of *Escherichia coli* BW25113. (A) *E. coli* growth curves in 1×M9 minimal medium with three different glucose concentrations (0.2%, 0.4%, and 0.8%) measured via OD_{600}. (B) *E. coli* growth curves in LB broth supplemented with four different NaCl concentrations (0%, 1%, 3.5%, and 5%) measured via OD_{600}. Three independent replicates were performed for the number of viable cells, the average values and standard error means were used to draw the curves.
