# Supplementary material for: Effects of NaCl Concentrations on Growth Patterns, Phenotypes Associated With Virulence, and Energy Metabolism in Escherichia coli BW25113
Source: Front Microbiol. 2021 Aug 16;12:705326. doi: 10.3389/fmicb.2021.705326 (PMC8415458; doi:10.3389/fmicb.2021.705326)
Supplement: Supplementary file 2 [file Image_2.pdf]

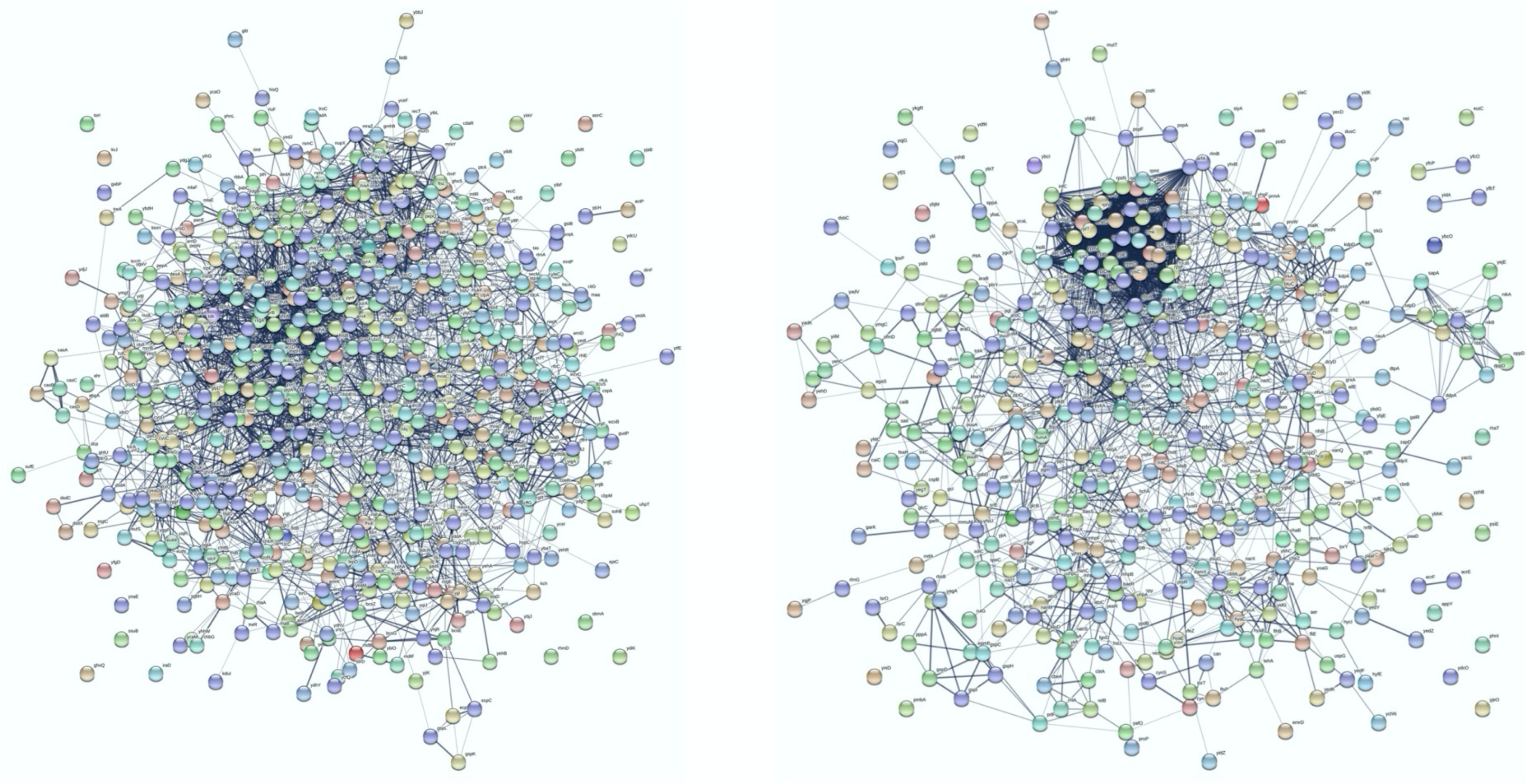

(A) (B)

Supplementary Figure 2 Protein-protein interaction (PPI) networks for (A) 653 up-regulated genes (proteins) and (B) 464 down-regulated genes (proteins) in *E. coli* sampels that were cultured in high salinity conditions (3.5% and 5% NaCl) and low salinity conditions (0% and 1% NaCl). Unconnected dots were reserved in the networks just to make sure that the data were complete.
