# Supplementary material for: Effects of NaCl Concentrations on Growth Patterns, Phenotypes Associated With Virulence, and Energy Metabolism in Escherichia coli BW25113
Source: Front Microbiol. 2021 Aug 16;12:705326. doi: 10.3389/fmicb.2021.705326 (PMC8415458; doi:10.3389/fmicb.2021.705326)
Supplement: Supplementary file 3 [file Image_3.pdf]

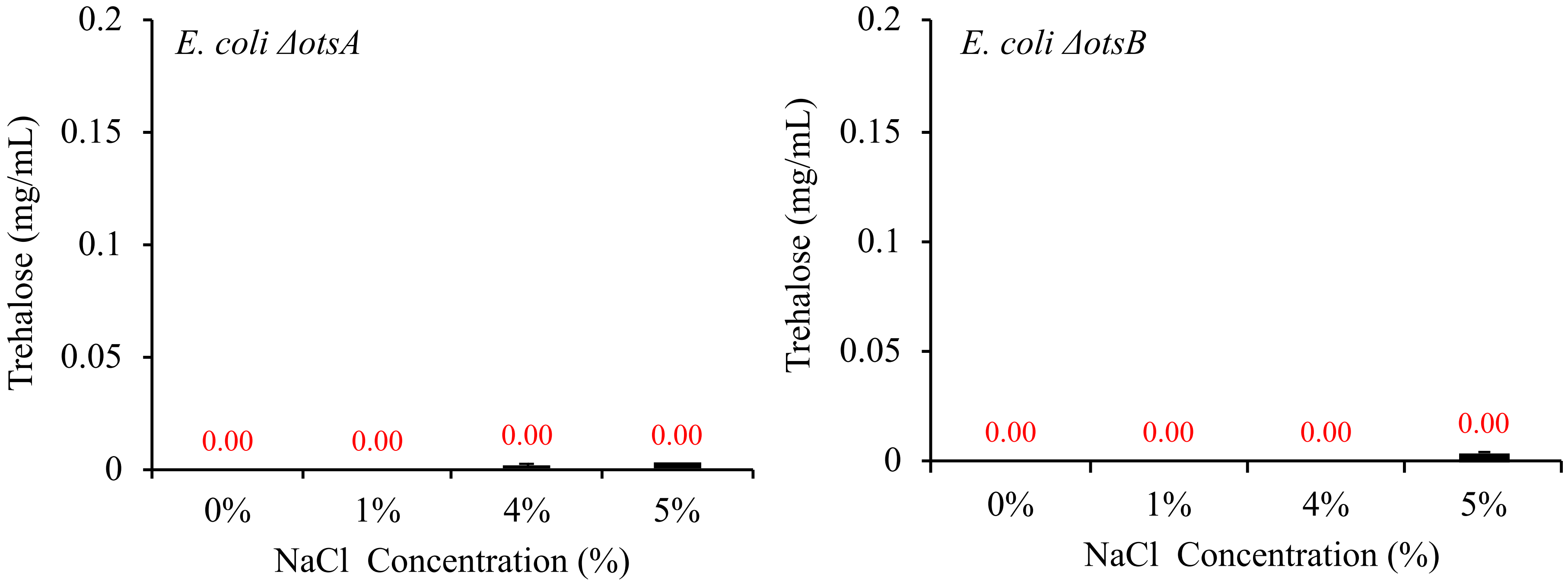

Supplementary Figure 3 Quantification of trehalose accumulation in *E. coli* BW25113 *ΔotsA* and *ΔotsB*. (A) *E. coli* BW25113 *ΔotsA*. (B) *E. coli* BW25113 *ΔotsB*. Three independent replicates were performed while the average values and standard error means were present.
