# Supplementary material for: Effects of NaCl Concentrations on Growth Patterns, Phenotypes Associated With Virulence, and Energy Metabolism in Escherichia coli BW25113
Source: Front Microbiol. 2021 Aug 16;12:705326. doi: 10.3389/fmicb.2021.705326 (PMC8415458; doi:10.3389/fmicb.2021.705326)
Supplement: Supplementary file 4 [file Table_1.docx]

**Supplementary Table 1** All of the up-regulated and down-regulated genes calculated via transcriptome analysis of the *E. coli* samples stimulated with high salinity and low salinity conditions.

| **Gene Groups** | **Gene Name** | **UniProt ID** | ***P*-value** | **Log2(Fold_Change)** | **Functions** |
| --- | --- | --- | --- | --- | --- |
| Up-regulated Genes | *stpA* | P0ACG1 | 2.23E-57 | 8.09 | DNA-binding protein StpA (H-NS homolog StpA) |
|  | *kch* | P31069 | 1.89E-31 | 7.76 | Voltage-gated potassium channel Kch |
|  | *ygaM* | P0ADQ7 | 6.19E-34 | 7.19 | Uncharacterized protein YgaM |
|  | *alaE* | P64550 | 2.00E-32 | 6.90 | L-alanine exporter AlaE |
|  | *wcaJ* | P71241 | 2.12E-08 | 6.37 | UDP-glucose:undecaprenyl-phosphate glucose-1-phosphate transferase (UDP-Glc:Und-P Glc-1-P transferase) (EC 2.7.8.31) (Colanic acid biosynthesis UDP-glucose lipid carrier transferase) (Glucosyl-P-P-undecaprenol synthase) |
|  | *nrdH* | P0AC65 | 2.24E-20 | 6.36 | Glutaredoxin-like protein NrdH |
|  | *leuC* | P0A6A6 | 4.03E-09 | 6.10 | 3-isopropylmalate dehydratase large subunit (EC 4.2.1.33) (Alpha-IPM isomerase) (IPMI) (Isopropylmalate isomerase) |
|  | *chbA* | P69791 | 1.78E-23 | 6.00 | PTS system N,N'-diacetylchitobiose-specific EIIA component (EIIA-Chb) (EIII-Chb) (IIIcel) (N,N'-diacetylchitobiose-specific phosphotransferase enzyme IIA component) |
|  | *evgS* | P30855 | 8.86E-120 | 5.91 | Sensor protein EvgS (EC 2.7.13.3) |
|  | *recQ* | P15043 | 4.86E-10 | 5.82 | ATP-dependent DNA helicase RecQ (EC 3.6.4.12) |
|  | *yegS* | P76407 | 4.47E-25 | 5.71 | Lipid kinase YegS (EC 2.7.1.-) |
|  | *leuD* | P30126 | 7.81E-09 | 5.71 | 3-isopropylmalate dehydratase small subunit (EC 4.2.1.33) (Alpha-IPM isomerase) (IPMI) (Isopropylmalate isomerase) |
|  | *fcl* | P32055 | 9.63E-07 | 5.42 | GDP-L-fucose synthase (EC 1.1.1.271) (GDP-4-keto-6-deoxy-D-mannose-3,5-epimerase-4-reductase) |
|  | *nrdI* | P0A772 | 4.91E-16 | 5.35 | Protein NrdI |
|  | *yceO* | P64442 | 3.31E-67 | 5.33 | Uncharacterized protein YceO |
|  | *gmm* | P32056 | 4.48E-06 | 5.23 | GDP-mannose mannosyl hydrolase (GDPMH) (EC 3.6.1.-) (Colanic acid biosynthesis protein WcaH) |
|  | *ygaV* | P77295 | 1.05E-23 | 5.09 | Probable HTH-type transcriptional regulator YgaV |
|  | *thrA* | P00561 | 1.58E-07 | 4.95 | Bifunctional aspartokinase/homoserine dehydrogenase 1 (Aspartokinase I/homoserine dehydrogenase I) (AKI-HDI) [Includes: Aspartokinase (EC 2.7.2.4); Homoserine dehydrogenase (EC 1.1.1.3)] |
|  | *ygaP* | P55734 | 6.68E-11 | 4.95 | Inner membrane protein YgaP |
|  | *kbp* | P0ADE6 | 7.28E-24 | 4.92 | Potassium binding protein Kbp (K(+) binding protein Kbp) |
|  | *wzxC* | P77377 | 1.92E-05 | 4.88 | Lipopolysaccharide biosynthesis protein WzxC |
|  | *ilvM* | P0ADG1 | 9.67E-07 | 4.81 | Acetolactate synthase isozyme 2 small subunit (EC 2.2.1.6) (ALS-II) (Acetohydroxy-acid synthase II small subunit) (AHAS-II) |
|  | *rfbB* | P37759 | 7.27E-06 | 4.62 | dTDP-glucose 4,6-dehydratase 1 (EC 4.2.1.46) |
|  | *rfbD* | P37760 | 1.72E-05 | 4.54 | dTDP-4-dehydrorhamnose reductase (EC 1.1.1.133) (dTDP-4-keto-L-rhamnose reductase) (dTDP-6-deoxy-L-lyxo-4-hexulose reductase) (dTDP-6-deoxy-L-mannose dehydrogenase) (dTDP-L-rhamnose synthase) |
|  | *rfbA* | P37744 | 1.05E-05 | 4.42 | Glucose-1-phosphate thymidylyltransferase 1 (G1P-TT 1) (EC 2.7.7.24) (dTDP-glucose pyrophosphorylase 1) (dTDP-glucose synthase 1) |
|  | *dsdX* | P08555 | 2.06E-19 | 4.25 | D-serine transporter DsdX |
|  | *rluE* | P75966 | 7.15E-13 | 4.19 | Ribosomal large subunit pseudouridine synthase E (EC 5.4.99.20) (rRNA pseudouridylate synthase E) (rRNA-uridine isomerase E) |
|  | *hxpB* | P77247 | 4.17E-10 | 4.15 | Hexitol phosphatase B (2-deoxyglucose-6-phosphate phosphatase) (EC 3.1.3.68) (Mannitol-1-phosphatase) (EC 3.1.3.22) (Sorbitol-6-phosphatase) (EC 3.1.3.50) (Sugar-phosphatase) (EC 3.1.3.23) |
|  | *gabP* | P25527 | 1.30E-35 | 4.12 | GABA permease (4-amino butyrate transport carrier) (Gamma-aminobutyrate permease) |
|  | *metH* | P13009 | 1.61E-07 | 4.08 | Methionine synthase (EC 2.1.1.13) (5-methyltetrahydrofolate--homocysteine methyltransferase) (Methionine synthase, vitamin-B12-dependent) (MS) |
|  | *gmd* | P0AC88 | 1.18E-04 | 4.01 | GDP-mannose 4,6-dehydratase (EC 4.2.1.47) (GDP-D-mannose dehydratase) |
|  | *mdtE* | P37636 | 3.63E-28 | 3.94 | Multidrug resistance protein MdtE |
|  | *ubiG* | P17993 | 6.38E-216 | 3.94 | Ubiquinone biosynthesis O-methyltransferase (2-octaprenyl-6-hydroxyphenol methylase) (EC 2.1.1.222) (3-demethylubiquinone-8 3-O-methyltransferase) (EC 2.1.1.64) |
|  | *dsdA* | P00926 | 5.30E-12 | 3.93 | D-serine dehydratase (EC 4.3.1.18) (D-serine deaminase) (DSD) |
|  | *ecpC* | P77802 | 2.92E-12 | 3.88 | Probable outer membrane usher protein EcpC |
|  | *galF* | P0AAB6 | 8.41E-05 | 3.86 | UTP--glucose-1-phosphate uridylyltransferase (EC 2.7.7.9) (Alpha-D-glucosyl-1-phosphate uridylyltransferase) (UDP-glucose pyrophosphorylase) (UDPGP) (Uridine diphosphoglucose pyrophosphorylase) |
|  | *carB* | P00968 | 3.02E-10 | 3.80 | Carbamoyl-phosphate synthase large chain (EC 6.3.5.5) (Carbamoyl-phosphate synthetase ammonia chain) |
|  | *emrK* | P52599 | 2.34E-79 | 3.80 | Probable multidrug resistance protein EmrK |
|  | *thrB* | P00547 | 2.88E-09 | 3.78 | Homoserine kinase (HK) (HSK) (EC 2.7.1.39) |
|  | *yjaB* | P09163 | 3.78E-24 | 3.78 | Peptidyl-lysine N-acetyltransferase YjaB (EC 2.3.1.-) (KAT) |
|  | *rsmI* | P67087 | 7.50E-27 | 3.66 | Ribosomal RNA small subunit methyltransferase I (EC 2.1.1.198) (16S rRNA 2'-O-ribose C1402 methyltransferase) (rRNA (cytidine-2'-O-)-methyltransferase RsmI) |
|  | *setA* | P31675 | 2.90E-05 | 3.65 | Sugar efflux transporter A |
|  | *nudJ* | P0AEI6 | 4.98E-11 | 3.65 | Phosphatase NudJ (EC 3.6.1.-) |
|  | *purH* | P15639 | 4.68E-26 | 3.64 | Bifunctional purine biosynthesis protein PurH [Includes: Phosphoribosylaminoimidazolecarboxamide formyltransferase (EC 2.1.2.3) (AICAR transformylase); IMP cyclohydrolase (EC 3.5.4.10) (ATIC) (IMP synthase) (Inosinicase)] |
|  | *bcsE* | P37657 | 4.95E-18 | 3.64 | Cyclic di-GMP binding protein BcsE (Cellulose biosynthesis protein BcsE) |
|  | *carA* | P0A6F1 | 9.94E-06 | 3.55 | Carbamoyl-phosphate synthase small chain (EC 6.3.5.5) (Carbamoyl-phosphate synthetase glutamine chain) |
|  | *psuT* | P33024 | 7.05E-04 | 3.50 | Putative pseudouridine transporter |
|  | *rfbX* | P37746 | 8.69E-05 | 3.47 | Putative O-antigen transporter |
|  | *rpoE* | P0AGB6 | 2.14E-08 | 3.46 | ECF RNA polymerase sigma-E factor (RNA polymerase sigma-E factor) (Sigma-24) |
|  | *thrC* | P00934 | 2.58E-08 | 3.46 | Threonine synthase (TS) (EC 4.2.3.1) |
|  | *rfbC* | P37745 | 3.85E-05 | 3.45 | dTDP-4-dehydrorhamnose 3,5-epimerase (EC 5.1.3.13) (Thymidine diphospho-4-keto-rhamnose 3,5-epimerase) (dTDP-4-keto-6-deoxyglucose 3,5-epimerase) (dTDP-6-deoxy-D-xylo-4-hexulose 3,5-epimerase) (dTDP-L-rhamnose synthase) |
|  | *yeiL* | P0A9E9 | 2.48E-04 | 3.43 | Regulatory protein YeiL |
|  | *fliI* | P52612 | 9.92E-15 | 3.41 | Flagellum-specific ATP synthase (EC 7.1.2.2) |
|  | *dsdC* | P46068 | 8.06E-28 | 3.37 | HTH-type transcriptional regulator DsdC (D-serine deaminase activator) |
|  | *dnaT* | P0A8J2 | 3.78E-10 | 3.35 | Primosomal protein 1 (Primosomal protein I) |
|  | *pxpB* | P0AAV4 | 1.88E-35 | 3.34 | 5-oxoprolinase subunit B (5-OPase subunit B) (EC 3.5.2.9) (5-oxoprolinase (ATP-hydrolyzing) subunit B) |
|  | *yhjR* | P0ADJ3 | 1.41E-12 | 3.33 | Protein YhjR |
|  | *rhtB* | P0AG34 | 8.98E-16 | 3.28 | Homoserine/homoserine lactone efflux protein |
|  | *sgrT* | C1P5Z7 | 9.83E-06 | 3.27 | Putative inhibitor of glucose uptake transporter SgrT |
|  | *nrdA* | P00452 | 9.01E-05 | 3.25 | Ribonucleoside-diphosphate reductase 1 subunit alpha (EC 1.17.4.1) (Protein B1) (Ribonucleoside-diphosphate reductase 1 R1 subunit) (Ribonucleotide reductase 1) |
|  | *gyrA* | P0AES4 | 5.20E-132 | 3.23 | DNA gyrase subunit A (EC 5.6.2.2) |
|  | *purD* | P15640 | 4.20E-24 | 3.19 | Phosphoribosylamine--glycine ligase (EC 6.3.4.13) (GARS) (Glycinamide ribonucleotide synthetase) (Phosphoribosylglycinamide synthetase) |
|  | *dsbG* | P77202 | 6.77E-11 | 3.18 | Thiol:disulfide interchange protein DsbG |
|  | *sucA* | P0AFG3 | 2.90E-33 | 3.15 | 2-oxoglutarate dehydrogenase E1 component (EC 1.2.4.2) (Alpha-ketoglutarate dehydrogenase) |
|  | *mutT* | P08337 | 9.30E-31 | 3.15 | 8-oxo-dGTP diphosphatase (8-oxo-dGTPase) (EC 3.6.1.55) (7,8-dihydro-8-oxoguanine-triphosphatase) (Mutator protein MutT) (dGTP pyrophosphohydrolase) |
|  | *nrdE* | P39452 | 4.88E-06 | 3.11 | Ribonucleoside-diphosphate reductase 2 subunit alpha (EC 1.17.4.1) (R1E protein) (Ribonucleotide reductase 2) |
|  | *hisC* | P06986 | 1.00E-07 | 3.10 | Histidinol-phosphate aminotransferase (EC 2.6.1.9) (Imidazole acetol-phosphate transaminase) (HPAT) (HspAT) |
|  | *ygfZ* | P0ADE8 | 2.04E-44 | 3.10 | tRNA-modifying protein YgfZ |
|  | *ldrD* | Q6BF25 | 1.26E-25 | 3.07 | Small toxic polypeptide LdrD |
|  | *mnmA* | P25745 | 4.04E-08 | 3.06 | tRNA-specific 2-thiouridylase MnmA (EC 2.8.1.13) |
|  | *hybB* | P37180 | 3.02E-07 | 3.05 | Probable Ni/Fe-hydrogenase 2 b-type cytochrome subunit |
|  | *yfbM* | P76483 | 6.07E-29 | 3.05 | Protein YfbM |
|  | *yddA* | P31826 | 5.06E-11 | 3.02 | Inner membrane ABC transporter ATP-binding protein YddA (CDS102) |
|  | *idnD* | P39346 | 2.08E-12 | 3.02 | L-idonate 5-dehydrogenase (NAD(P)(+)) (EC 1.1.1.264) |
|  | *nuoM* | P0AFE8 | 2.72E-27 | 3.02 | NADH-quinone oxidoreductase subunit M (EC 7.1.1.-) (NADH dehydrogenase I subunit M) (NDH-1 subunit M) (NUO13) |
|  | *ydjM* | P64481 | 4.51E-05 | 3.00 | Inner membrane protein YdjM |
|  | *yhjV* | P37660 | 2.45E-16 | 2.96 | Inner membrane transport protein YhjV |
|  | *gadW* | P63201 | 6.00E-16 | 2.93 | HTH-type transcriptional regulator GadW |
|  | *pepN* | P04825 | 2.80E-06 | 2.91 | Aminopeptidase N (EC 3.4.11.2) (Alpha-aminoacylpeptide hydrolase) |
|  | *nuoN* | P0AFF0 | 4.20E-29 | 2.91 | NADH-quinone oxidoreductase subunit N (EC 7.1.1.-) (NADH dehydrogenase I subunit N) (NDH-1 subunit N) (NUO14) |
|  | *nuoK* | P0AFE4 | 2.50E-17 | 2.90 | NADH-quinone oxidoreductase subunit K (EC 7.1.1.-) (NADH dehydrogenase I subunit K) (NDH-1 subunit K) (NUO11) |
|  | *fes* | P13039 | 9.13E-05 | 2.90 | Iron(III) enterobactin esterase (EC 3.1.1.108) (Enterochelin esterase) (Ferric enterobactin esterase) |
|  | *ftsN* | P29131 | 1.48E-07 | 2.90 | Cell division protein FtsN |
|  | *lysU* | P0A8N5 | 1.60E-40 | 2.88 | Lysine--tRNA ligase, heat inducible (EC 6.1.1.6) (Lysyl-tRNA synthetase) (LysRS) |
|  | *nrdB* | P69924 | 3.23E-03 | 2.87 | Ribonucleoside-diphosphate reductase 1 subunit beta (EC 1.17.4.1) (Protein B2) (Protein R2) (Ribonucleotide reductase 1) |
|  | *bcsG* | P37659 | 2.46E-17 | 2.86 | Cellulose biosynthesis protein BcsG |
|  | *gcl* | P0AEP7 | 6.34E-24 | 2.85 | Glyoxylate carboligase (EC 4.1.1.47) (Tartronate-semialdehyde synthase) |
|  | *mntP* | P76264 | 1.99E-106 | 2.84 | Probable manganese efflux pump MntP |
|  | *fumD* | P0ACX5 | 2.64E-06 | 2.84 | Fumarase D (EC 4.2.1.2) |
|  | *evgA* | P0ACZ4 | 5.09E-44 | 2.83 | DNA-binding transcriptional activator EvgA |
|  | *lpp* | P69776 | 4.24E-07 | 2.82 | Major outer membrane lipoprotein Lpp (Braun lipoprotein) (BLP) (Murein-lipoprotein) |
|  | *dpiB* | P77510 | 4.46E-20 | 2.81 | Sensor histidine kinase DpiB (EC 2.7.13.3) (Sensor histidine kinase CitA) |
|  | *hemC* | P06983 | 1.03E-51 | 2.80 | Porphobilinogen deaminase (PBG) (EC 2.5.1.61) (Hydroxymethylbilane synthase) (HMBS) (Pre-uroporphyrinogen synthase) |
|  | *pykF* | P0AD61 | 1.80E-06 | 2.80 | Pyruvate kinase I (EC 2.7.1.40) (PK-1) |
|  | *melB* | P02921 | 5.85E-46 | 2.79 | Melibiose carrier protein (Melibiose permease) (Melibiose transporter) (Na+ (Li+)/melibiose symporter) (Thiomethylgalactoside permease II) |
|  | *acrZ* | P0AAW9 | 2.54E-35 | 2.79 | Multidrug efflux pump accessory protein AcrZ (AcrAB-TolC multidrug efflux pump accessory protein AcrZ) (Acridine resistance protein Z) |
|  | *sdhE* | P64559 | 1.84E-12 | 2.76 | FAD assembly factor SdhE (Antitoxin CptB) |
|  | *bglB* | P11988 | 1.06E-74 | 2.74 | 6-phospho-beta-glucosidase BglB (EC 3.2.1.86) (Phospho-beta-glucosidase B) |
|  | *pncB* | P18133 | 3.05E-05 | 2.74 | Nicotinate phosphoribosyltransferase (NAPRTase) (EC 6.3.4.21) |
|  | *fruK* | P0AEW9 | 3.98E-15 | 2.73 | 1-phosphofructokinase (EC 2.7.1.56) (Fructose 1-phosphate kinase) |
|  | *murE* | P22188 | 5.48E-07 | 2.72 | UDP-N-acetylmuramoyl-L-alanyl-D-glutamate--2,6-diaminopimelate ligase (EC 6.3.2.13) (Meso-A2pm-adding enzyme) (Meso-diaminopimelate-adding enzyme) (UDP-MurNAc-L-Ala-D-Glu:meso-diaminopimelate ligase) (UDP-MurNAc-tripeptide synthetase) (UDP-N-acetylmuramyl-tripeptide synthetase) |
|  | *mdtF* | P37637 | 2.50E-15 | 2.72 | Multidrug resistance protein MdtF |
|  | *ccmA* | P33931 | 3.20E-16 | 2.72 | Cytochrome c biogenesis ATP-binding export protein CcmA (EC 7.6.2.5) (Heme exporter protein A) |
|  | *gpsA* | P0A6S7 | 1.70E-08 | 2.70 | Glycerol-3-phosphate dehydrogenase [NAD(P)+] (EC 1.1.1.94) (NAD(P)H-dependent glycerol-3-phosphate dehydrogenase) |
|  | *rbsK* | P0A9J6 | 4.57E-15 | 2.70 | Ribokinase (RK) (EC 2.7.1.15) |
|  | *ygfX* | Q46824 | 7.81E-16 | 2.69 | Inner membrane protein YgfX (Toxin CptA) |
|  | *ftsI* | P0AD68 | 1.79E-10 | 2.68 | Peptidoglycan D,D-transpeptidase FtsI (EC 3.4.16.4) (Essential cell division protein FtsI) (Murein transpeptidase) (Penicillin-binding protein 3) (PBP-3) (Peptidoglycan synthase FtsI) |
|  | *ybiO* | P75783 | 2.54E-14 | 2.67 | Moderate conductance mechanosensitive channel YbiO |
|  | *sufE* | P76194 | 8.41E-17 | 2.66 | Cysteine desulfuration protein SufE |
|  | *yiaV* | P37683 | 2.53E-80 | 2.66 | Inner membrane protein YiaV |
|  | *uhpT* | P0AGC0 | 2.93E-11 | 2.65 | Hexose-6-phosphate:phosphate antiporter |
|  | *modE* | P0A9G8 | 3.99E-37 | 2.64 | DNA-binding transcriptional dual regulator ModE |
|  | *pepQ* | P21165 | 1.22E-15 | 2.64 | Xaa-Pro dipeptidase (X-Pro dipeptidase) (EC 3.4.13.9) (Imidodipeptidase) (Proline dipeptidase) (Prolidase) |
|  | *ydhY* | P0AAL6 | 1.28E-05 | 2.63 | Uncharacterized ferredoxin-like protein YdhY |
|  | *clpB* | P63284 | 3.38E-08 | 2.62 | Chaperone protein ClpB (Heat shock protein F84.1) |
|  | *eutQ* | P76555 | 5.81E-07 | 2.62 | Ethanolamine utilization protein EutQ |
|  | *ftsL* | P0AEN4 | 6.05E-13 | 2.62 | Cell division protein FtsL |
|  | *rsmH* | P60390 | 2.15E-12 | 2.61 | Ribosomal RNA small subunit methyltransferase H (EC 2.1.1.199) (16S rRNA m(4)C1402 methyltransferase) (rRNA (cytosine-N(4)-)-methyltransferase RsmH) |
|  | *wzyE* | P27835 | 1.75E-08 | 2.61 | Probable ECA polymerase |
|  | *yiaD* | P37665 | 2.43E-24 | 2.59 | Probable lipoprotein YiaD |
|  | *glgA* | P0A6U8 | 3.14E-25 | 2.59 | Glycogen synthase (EC 2.4.1.21) (Starch [bacterial glycogen] synthase) |
|  | *cbpM* | P63264 | 2.19E-24 | 2.57 | Chaperone modulatory protein CbpM |
|  | *melA* | P06720 | 2.61E-44 | 2.56 | Alpha-galactosidase (EC 3.2.1.22) (Melibiase) |
|  | *ilvE* | P0AB80 | 2.47E-03 | 2.56 | Branched-chain-amino-acid aminotransferase (BCAT) (EC 2.6.1.42) (Transaminase B) |
|  | *rihB* | P33022 | 1.76E-05 | 2.56 | Pyrimidine-specific ribonucleoside hydrolase RihB (EC 3.2.2.8) (Cytidine/uridine-specific hydrolase) |
|  | *gpmA* | P62707 | 6.70E-07 | 2.54 | 2,3-bisphosphoglycerate-dependent phosphoglycerate mutase (BPG-dependent PGAM) (PGAM) (Phosphoglyceromutase) (dPGM) (EC 5.4.2.11) |
|  | *chbG* | P37794 | 1.52E-14 | 2.52 | Chitooligosaccharide deacetylase ChbG (COD) (EC 3.5.1.105) (Chitin disaccharide deacetylase) (Chitobiose deacetylase) (Chitobiose-6P deacetylase) (Chitotriose deacetylase) (Chitotriose-6P deacetylase) |
|  | *aceB* | P08997 | 1.49E-11 | 2.52 | Malate synthase A (MSA) (EC 2.3.3.9) |
|  | *nuoJ* | P0AFE0 | 1.87E-13 | 2.51 | NADH-quinone oxidoreductase subunit J (EC 7.1.1.-) (NADH dehydrogenase I subunit J) (NDH-1 subunit J) (NUO10) |
|  | *rlmA* | P36999 | 8.33E-58 | 2.51 | 23S rRNA (guanine(745)-N(1))-methyltransferase (EC 2.1.1.187) (23S rRNA m1G745 methyltransferase) (Ribosomal RNA large subunit methyltransferase A) |
|  | *azoR* | P41407 | 1.28E-14 | 2.48 | FMN-dependent NADH:quinone oxidoreductase (EC 1.6.5.-) (Azo-dye reductase) (FMN-dependent NADH-azo compound oxidoreductase) (FMN-dependent NADH-azoreductase) (EC 1.7.1.17) |
|  | *gltA* | P0ABH7 | 8.93E-28 | 2.48 | Citrate synthase (EC 2.3.3.16) |
|  | *fepA* | P05825 | 1.74E-05 | 2.47 | Ferrienterobactin receptor (Enterobactin outer-membrane receptor) |
|  | *lamB* | P02943 | 3.46E-15 | 2.47 | Maltoporin (Maltose outer membrane channel) (Maltose-inducible porin) (Phage lambda receptor protein) |
|  | *ydiV* | P76204 | 1.00E-14 | 2.45 | Putative anti-FlhC(2)FlhD(4) factor YdiV (c-di-GMP regulator CdgR) |
|  | *emrY* | P52600 | 6.32E-17 | 2.45 | Probable multidrug resistance protein EmrY |
|  | *eutD* | P77218 | 1.11E-06 | 2.43 | Ethanolamine utilization protein EutD |
|  | *metI* | P31547 | 2.18E-13 | 2.41 | D-methionine transport system permease protein MetI |
|  | *argR* | P0A6D0 | 4.29E-18 | 2.40 | Arginine repressor |
|  | *scpA* | P27253 | 3.21E-09 | 2.36 | Methylmalonyl-CoA mutase (MCM) (EC 5.4.99.2) |
|  | *paoD* | P77183 | 1.28E-09 | 2.36 | Molybdenum cofactor insertion chaperone PaoD |
|  | *yibF* | P0ACA1 | 2.22E-09 | 2.34 | Uncharacterized GST-like protein YibF |
|  | *gor* | P06715 | 1.12E-09 | 2.33 | Glutathione reductase (GR) (GRase) (EC 1.8.1.7) |
|  | *murF* | P11880 | 4.57E-05 | 2.33 | UDP-N-acetylmuramoyl-tripeptide--D-alanyl-D-alanine ligase (EC 6.3.2.10) (D-alanyl-D-alanine-adding enzyme) (UDP-MurNAc-pentapeptide synthetase) |
|  | *yrbL* | P64610 | 7.20E-09 | 2.32 | Uncharacterized protein YrbL |
|  | *recC* | P07648 | 1.26E-37 | 2.31 | RecBCD enzyme subunit RecC (EC 3.1.11.5) (Exodeoxyribonuclease V 125 kDa polypeptide) (Exodeoxyribonuclease V gamma chain) (Exonuclease V subunit RecC) (ExoV subunit RecC) |
|  | *moaB* | P0AEZ9 | 3.27E-18 | 2.31 | Molybdenum cofactor biosynthesis protein B |
|  | *mlaF* | P63386 | 2.36E-56 | 2.30 | Intermembrane phospholipid transport system ATP-binding protein MlaF (EC 7.6.2.-) |
|  | *murD* | P14900 | 1.89E-07 | 2.30 | UDP-N-acetylmuramoylalanine--D-glutamate ligase (EC 6.3.2.9) (D-glutamic acid-adding enzyme) (UDP-N-acetylmuramoyl-L-alanyl-D-glutamate synthetase) |
|  | *mraZ* | P22186 | 1.95E-10 | 2.29 | Transcriptional regulator MraZ |
|  | *nuoH* | P0AFD4 | 3.15E-08 | 2.29 | NADH-quinone oxidoreductase subunit H (EC 7.1.1.-) (NADH dehydrogenase I subunit H) (NDH-1 subunit H) (NUO8) |
|  | *aldB* | P37685 | 2.42E-15 | 2.29 | Aldehyde dehydrogenase B (EC 1.2.1.4) (Acetaldehyde dehydrogenase) |
|  | *glf* | P37747 | 1.88E-35 | 2.28 | UDP-galactopyranose mutase (UGM) (EC 5.4.99.9) (UDP-GALP mutase) (Uridine 5-diphosphate galactopyranose mutase) |
|  | *eutM* | P0ABF4 | 6.04E-07 | 2.28 | Ethanolamine utilization protein EutM |
|  | *talB* | P0A870 | 9.18E-29 | 2.26 | Transaldolase B (EC 2.2.1.2) |
|  | *dinB* | Q47155 | 2.92E-22 | 2.26 | DNA polymerase IV (Pol IV) (EC 2.7.7.7) (Translesion synthesis polymerase IV) (TSL polymerase IV) |
|  | *rhlB* | P0A8J8 | 1.17E-11 | 2.26 | ATP-dependent RNA helicase RhlB (EC 3.6.4.13) |
|  | *emtA* | P0C960 | 6.05E-60 | 2.25 | Endo-type membrane-bound lytic murein transglycosylase A (EC 4.2.2.n2) (Peptidoglycan lytic endotransglycosylase) |
|  | *rpiB* | P37351 | 7.40E-08 | 2.24 | Ribose-5-phosphate isomerase B (EC 5.3.1.6) (Phosphoriboisomerase B) |
|  | *rstB* | P18392 | 3.30E-12 | 2.24 | Sensor protein RstB (EC 2.7.13.3) |
|  | *menH* | P37355 | 9.67E-03 | 2.23 | 2-succinyl-6-hydroxy-2,4-cyclohexadiene-1-carboxylate synthase (SHCHC synthase) (EC 4.2.99.20) |
|  | *yojI* | P33941 | 2.00E-26 | 2.22 | ABC transporter ATP-binding/permease protein YojI |
|  | *ycaM* | P75835 | 1.09E-06 | 2.21 | Inner membrane transporter YcaM |
|  | *ftsK* | P46889 | 9.21E-14 | 2.21 | DNA translocase FtsK |
|  | *yafN* | Q47156 | 2.68E-30 | 2.20 | Antitoxin YafN |
|  | *comR* | P75952 | 6.09E-48 | 2.19 | HTH-type transcriptional repressor ComR (Copper outer membrane regulator) |
|  | *yciN* | P0AB61 | 2.87E-25 | 2.19 | Protein YciN |
|  | *hemD* | P09126 | 3.40E-43 | 2.19 | Uroporphyrinogen-III synthase (UROS) (EC 4.2.1.75) (Hydroxymethylbilane hydrolyase [cyclizing]) (Uroporphyrinogen-III cosynthase) |
|  | *yrbG* | P45394 | 6.55E-44 | 2.18 | Inner membrane protein YrbG |
|  | *xseB* | P0A8G9 | 6.73E-05 | 2.18 | Exodeoxyribonuclease 7 small subunit (EC 3.1.11.6) (Exodeoxyribonuclease VII small subunit) (Exonuclease VII small subunit) |
|  | *tdcC* | P0AAD8 | 1.38E-30 | 2.18 | Threonine/serine transporter TdcC (H(+)/threonine-serine symporter) |
|  | *hns* | P0ACF8 | 7.63E-04 | 2.17 | DNA-binding protein H-NS (Heat-stable nucleoid-structuring protein) (Histone-like protein HLP-II) (Protein B1) (Protein H1) |
|  | *purN* | P08179 | 4.19E-10 | 2.16 | Phosphoribosylglycinamide formyltransferase (EC 2.1.2.2) (5'-phosphoribosylglycinamide transformylase) (GAR transformylase) (GART) |
|  | *purB* | P0AB89 | 1.90E-08 | 2.16 | Adenylosuccinate lyase (ASL) (EC 4.3.2.2) (Adenylosuccinase) (ASase) |
|  | *elbB* | P0ABU5 | 3.40E-08 | 2.15 | Glyoxalase ElbB (EC 4.2.1.-) (Sigma cross-reacting protein 27A) (SCRP-27A) |
|  | *pldB* | P07000 | 1.66E-30 | 2.15 | Lysophospholipase L2 (EC 3.1.1.5) (Lecithinase B) |
|  | *pdxA* | P19624 | 4.77E-10 | 2.15 | 4-hydroxythreonine-4-phosphate dehydrogenase (EC 1.1.1.262) (4-(phosphohydroxy)-L-threonine dehydrogenase) |
|  | *sdhD* | P0AC44 | 2.33E-14 | 2.14 | Succinate dehydrogenase hydrophobic membrane anchor subunit |
|  | *arcB* | P0AEC3 | 2.88E-09 | 2.14 | Aerobic respiration control sensor protein ArcB (EC 2.7.13.3) |
|  | *rutB* | P75897 | 9.74E-08 | 2.14 | Ureidoacrylate amidohydrolase RutB (EC 3.5.1.110) |
|  | *mfd* | P30958 | 2.30E-24 | 2.14 | Transcription-repair-coupling factor (TRCF) (EC 3.6.4.-) |
|  | *tdcE* | P42632 | 6.23E-10 | 2.13 | PFL-like enzyme TdcE (Keto-acid formate acetyltransferase) (Keto-acid formate-lyase) (Ketobutyrate formate-lyase) (KFL) (EC 2.3.1.-) (Pyruvate formate-lyase) (PFL) (EC 2.3.1.54) |
|  | *ybjO* | P0AAZ0 | 2.69E-40 | 2.13 | Inner membrane protein YbjO |
|  | *vsr* | P09184 | 3.79E-06 | 2.11 | Very short patch repair protein (EC 3.1.-.-) (DNA mismatch endonuclease) (V.EcoKDcm) (Vsr mismatch endonuclease) |
|  | *bisC* | P20099 | 3.69E-41 | 2.11 | Biotin sulfoxide reductase (BDS reductase) (BSO reductase) (EC 1.-.-.-) (L-methionine-(S)-sulfoxide reductase) (Met-S-SO reductase) (EC 1.8.4.13) |
|  | *mtgA* | P46022 | 4.79E-08 | 2.11 | Biosynthetic peptidoglycan transglycosylase (EC 2.4.1.129) (Glycan polymerase) (Monofunctional biosynthetic peptidoglycan transglycosylase) (Monofunctional glycosyltransferase) (Monofunctional GTase) (Peptidoglycan glycosyltransferase MtgA) (PGT) |
|  | *gatD* | P0A9S3 | 6.94E-27 | 2.09 | Galactitol 1-phosphate 5-dehydrogenase (EC 1.1.1.251) |
|  | *scpB* | P52045 | 1.91E-20 | 2.08 | Methylmalonyl-CoA decarboxylase (MMCD) (EC 4.1.1.-) (Transcarboxylase) |
|  | *potH* | P31135 | 2.47E-19 | 2.08 | Putrescine transport system permease protein PotH |
|  | *hflD* | P25746 | 2.52E-08 | 2.08 | High frequency lysogenization protein HflD |
|  | *gadE* | P63204 | 2.24E-09 | 2.06 | Transcriptional regulator GadE |
|  | *moaD* | P30748 | 2.06E-13 | 2.05 | Molybdopterin synthase sulfur carrier subunit (MPT synthase subunit 1) (Molybdenum cofactor biosynthesis protein D) (Molybdopterin-converting factor small subunit) (Molybdopterin-converting factor subunit 1) (Sulfur carrier protein MoaD) |
|  | *ftsZ* | P0A9A6 | 1.26E-15 | 2.05 | Cell division protein FtsZ |
|  | *dosC* | P0AA89 | 8.88E-06 | 2.05 | Diguanylate cyclase DosC (DGC) (EC 2.7.7.65) (Direct oxygen-sensing cyclase) |
|  | *hcxA* | P45579 | 6.96E-13 | 2.04 | Hydroxycarboxylate dehydrogenase A (EC 1.1.1.-) (2-oxobutanoate reductase) (2-oxoglutarate reductase) |
|  | *ghrB* | P37666 | 6.85E-20 | 2.04 | Glyoxylate/hydroxypyruvate reductase B (EC 1.1.1.79) (EC 1.1.1.81) (2-ketoaldonate reductase) (2-ketogluconate reductase) (2KR) (EC 1.1.1.215) |
|  | *ynjC* | P76224 | 1.76E-10 | 2.04 | Inner membrane ABC transporter permease protein YnjC |
|  | *ydiK* | P0AFS7 | 1.18E-05 | 2.03 | Putative transport protein YdiK |
|  | *allS* | P0ACR0 | 6.66E-06 | 2.03 | HTH-type transcriptional activator AllS |
|  | *moaC* | P0A738 | 2.94E-20 | 2.03 | Cyclic pyranopterin monophosphate synthase (EC 4.6.1.17) (Molybdenum cofactor biosynthesis protein C) |
|  | *gmhB* | P63228 | 1.19E-07 | 2.02 | D-glycero-beta-D-manno-heptose-1,7-bisphosphate 7-phosphatase (EC 3.1.3.82) (D,D-heptose 1,7-bisphosphate phosphatase) (HBP phosphatase) |
|  | *degP* | P0C0V0 | 1.79E-34 | 2.02 | Periplasmic serine endoprotease DegP (EC 3.4.21.107) (Heat shock protein DegP) (Protease Do) |
|  | *ribB* | P0A7J0 | 1.12E-09 | 2.02 | 3,4-dihydroxy-2-butanone 4-phosphate synthase (DHBP synthase) (EC 4.1.99.12) |
|  | *bluF* | P75990 | 1.31E-08 | 2.02 | Blue light- and temperature-regulated antirepressor BluF (Blrp) |
|  | *cbrA* | P31456 | 2.21E-49 | 2.02 | Protein CbrA (CreB-regulated gene A protein) |
|  | *bcsZ* | P37651 | 7.45E-14 | 2.02 | Endoglucanase (EC 3.2.1.4) (Carboxymethylcellulase) (CMCase) (Cellulase) (Endo-1,4-beta-glucanase) |
|  | *soxS* | P0A9E2 | 1.29E-08 | 2.02 | Regulatory protein SoxS |
|  | *dcm* | P0AED9 | 2.58E-19 | 2.02 | DNA-cytosine methyltransferase (EC 2.1.1.37) (M.EcoDcm) |
|  | *dosP* | P76129 | 8.48E-07 | 2.01 | Oxygen sensor protein DosP (EC 3.1.4.52) (Direct oxygen-sensing phosphodiesterase) (Direct oxygen sensor protein) (Ec DOS) (Heme-regulated cyclic di-GMP phosphodiesterase) |
|  | *torI* | Q2EES9 | 2.51E-04 | 2.01 | Response regulator inhibitor for tor operon (Tor inhibitor) |
|  | *btuD* | P06611 | 2.49E-13 | 2.01 | Vitamin B12 import ATP-binding protein BtuD (EC 7.6.2.8) (Vitamin B12-transporting ATPase) |
|  | *deoD* | P0ABP8 | 6.78E-34 | 2.00 | Purine nucleoside phosphorylase DeoD-type (PNP) (EC 2.4.2.1) |
|  | *fdnI* | P0AEK7 | 2.27E-11 | 2.00 | Formate dehydrogenase, nitrate-inducible, cytochrome b556(Fdn) subunit (Anaerobic formate dehydrogenase cytochrome b556 subunit) (Formate dehydrogenase-N subunit gamma) (FDH-N subunit gamma) |
|  | *rffG* | P27830 | 1.74E-09 | 2.00 | dTDP-glucose 4,6-dehydratase 2 (EC 4.2.1.46) |
|  | *elaD* | Q47013 | 1.69E-18 | 1.99 | Protease ElaD (EC 3.4.22.-) (Deubiquitinase) (Deubiquitinating enzyme) (DUB) (Deubiquitinating protease) |
|  | *fadR* | P0A8V6 | 3.41E-06 | 1.98 | Fatty acid metabolism regulator protein |
|  | *rutA* | P75898 | 3.51E-06 | 1.98 | Pyrimidine monooxygenase RutA (EC 1.14.99.46) |
|  | *mraY* | P0A6W3 | 2.65E-04 | 1.98 | Phospho-N-acetylmuramoyl-pentapeptide-transferase (EC 2.7.8.13) (UDP-MurNAc-pentapeptide phosphotransferase) |
|  | *argK* | P27254 | 5.98E-27 | 1.97 | GTPase ArgK (EC 3.6.5.-) (G-protein chaperone) |
|  | *mdtD* | P36554 | 1.41E-20 | 1.97 | Putative multidrug resistance protein MdtD |
|  | *asnC* | P0ACI6 | 9.79E-05 | 1.97 | Regulatory protein AsnC |
|  | *ndh* | P00393 | 8.62E-27 | 1.96 | NADH dehydrogenase (EC 7.1.1.2) |
|  | *purA* | P0A7D4 | 8.35E-21 | 1.95 | Adenylosuccinate synthetase (AMPSase) (AdSS) (EC 6.3.4.4) (IMP--aspartate ligase) |
|  | *ahr* | P27250 | 2.60E-09 | 1.95 | Aldehyde reductase Ahr (EC 1.1.1.2) (Zinc-dependent alcohol dehydrogenase Ahr) |
|  | *mqo* | P33940 | 1.51E-28 | 1.94 | Malate:quinone oxidoreductase (EC 1.1.5.4) (MQO) (Malate dehydrogenase [quinone]) |
|  | *ynbD* | P76093 | 8.92E-09 | 1.93 | Uncharacterized protein YnbD |
|  | *trxA* | P0AA25 | 3.64E-07 | 1.93 | Thioredoxin 1 (Trx-1) |
|  | *lpxC* | P0A725 | 2.81E-18 | 1.93 | UDP-3-O-acyl-N-acetylglucosamine deacetylase (UDP-3-O-acyl-GlcNAc deacetylase) (EC 3.5.1.108) (Protein EnvA) (UDP-3-O-[R-3-hydroxymyristoyl]-N-acetylglucosamine deacetylase) |
|  | *phoR* | P08400 | 1.62E-05 | 1.93 | Phosphate regulon sensor protein PhoR (EC 2.7.13.3) |
|  | *ldcA* | P76008 | 1.12E-11 | 1.93 | Murein tetrapeptide carboxypeptidase (EC 3.4.17.13) (LD-carboxypeptidase A) (Muramoyltetrapeptide carboxypeptidase) |
|  | *mlaE* | P64606 | 1.08E-43 | 1.92 | Intermembrane phospholipid transport system permease protein MlaE |
|  | *casC* | Q46899 | 1.67E-04 | 1.91 | CRISPR system Cascade subunit CasC |
|  | *yedA* | P0AA70 | 2.42E-19 | 1.91 | Uncharacterized inner membrane transporter YedA |
|  | *alkB* | P05050 | 2.13E-10 | 1.91 | Alpha-ketoglutarate-dependent dioxygenase AlkB (EC 1.14.11.33) (Alkylated DNA repair protein AlkB) (DNA oxidative demethylase AlkB) |
|  | *fucI* | P69922 | 8.10E-07 | 1.91 | L-fucose isomerase (FucIase) (EC 5.3.1.25) (6-deoxy-L-galactose isomerase) (D-arabinose isomerase) (EC 5.3.1.3) |
|  | *hisB* | P06987 | 6.73E-16 | 1.91 | Histidine biosynthesis bifunctional protein HisB [Includes: Histidinol-phosphatase (EC 3.1.3.15); Imidazoleglycerol-phosphate dehydratase (IGPD) (EC 4.2.1.19)] |
|  | *ybiA* | P30176 | 4.58E-17 | 1.90 | N-glycosidase YbiA (EC 3.2.2.-) (Riboflavin biosynthesis intermediates N-glycosidase) |
|  | *lolE* | P75958 | 2.10E-40 | 1.89 | Lipoprotein-releasing system transmembrane protein LolE |
|  | *dinF* | P28303 | 9.09E-32 | 1.88 | DNA damage-inducible protein F |
|  | *pth* | P0A7D1 | 7.03E-51 | 1.88 | Peptidyl-tRNA hydrolase (PTH) (EC 3.1.1.29) |
|  | *miaB* | P0AEI1 | 1.41E-05 | 1.88 | tRNA-2-methylthio-N(6)-dimethylallyladenosine synthase (EC 2.8.4.3) ((Dimethylallyl)adenosine tRNA methylthiotransferase MiaB) (tRNA-i(6)A37 methylthiotransferase) |
|  | *entD* | P19925 | 9.06E-04 | 1.87 | Enterobactin synthase component D (4'-phosphopantetheinyl transferase EntD) (EC 2.7.8.-) (Enterochelin synthase D) |
|  | *nhaB* | P0AFA7 | 2.52E-04 | 1.87 | Na(+)/H(+) antiporter NhaB (Sodium/proton antiporter NhaB) |
|  | *ftsW* | P0ABG4 | 1.58E-05 | 1.87 | Probable peptidoglycan glycosyltransferase FtsW (PGT) (EC 2.4.1.129) (Cell division protein FtsW) (Cell wall polymerase) (Lipid II flippase FtsW) (Peptidoglycan polymerase) (PG polymerase) |
|  | *torD* | P36662 | 6.31E-12 | 1.86 | Chaperone protein TorD |
|  | *ynjB* | P76223 | 5.25E-11 | 1.86 | Protein YnjB |
|  | *cheA* | P07363 | 3.05E-06 | 1.86 | Chemotaxis protein CheA (EC 2.7.13.3) |
|  | *ecpB* | P77188 | 2.84E-06 | 1.86 | Probable fimbrial chaperone EcpB |
|  | *abgA* | P77357 | 1.21E-04 | 1.86 | p-aminobenzoyl-glutamate hydrolase subunit A (EC 3.5.1.-) (PABA-GLU hydrolase) (PGH) |
|  | *nudL* | P43337 | 5.06E-09 | 1.86 | Uncharacterized Nudix hydrolase NudL (EC 3.6.1.-) |
|  | *tdcD* | P11868 | 5.40E-13 | 1.86 | Propionate kinase (EC 2.7.2.15) |
|  | *cusS* | P77485 | 5.27E-06 | 1.85 | Sensor histidine kinase CusS (EC 2.7.13.3) |
|  | *cnoX* | P77395 | 1.08E-22 | 1.85 | Chaperedoxin (Heat shock protein CnoX) (Trxsc) |
|  | *ynaE* | P76073 | 7.78E-06 | 1.84 | Uncharacterized protein YnaE |
|  | *yghA* | P0AG84 | 3.63E-09 | 1.83 | Uncharacterized oxidoreductase YghA (EC 1.-.-.-) |
|  | *galU* | P0AEP3 | 4.25E-03 | 1.83 | UTP--glucose-1-phosphate uridylyltransferase (EC 2.7.7.9) (Alpha-D-glucosyl-1-phosphate uridylyltransferase) (UDP-glucose pyrophosphorylase) (UDPGP) (Uridine diphosphoglucose pyrophosphorylase) |
|  | *yibL* | P0ADK8 | 1.17E-14 | 1.83 | Uncharacterized protein YibL |
|  | *ppk* | P0A7B1 | 8.64E-10 | 1.83 | Polyphosphate kinase (EC 2.7.4.1) (ATP-polyphosphate phosphotransferase) (Polyphosphoric acid kinase) |
|  | *rraA* | P0A8R0 | 1.84E-06 | 1.82 | Regulator of ribonuclease activity A |
|  | *cspA* | P0A9X9 | 3.65E-06 | 1.82 | Cold shock protein CspA (CSP-A) (7.4 kDa cold shock protein) (CS7.4) |
|  | *bglG* | P11989 | 3.11E-39 | 1.82 | Cryptic beta-glucoside bgl operon antiterminator |
|  | *glgS* | P26649 | 2.26E-26 | 1.82 | Surface composition regulator |
|  | *ptrA* | P05458 | 3.23E-14 | 1.81 | Protease 3 (EC 3.4.24.55) (Pitrilysin) (Protease III) (Protease pi) |
|  | *ytfE* | P69506 | 1.93E-35 | 1.81 | Iron-sulfur cluster repair protein YtfE (Regulator of cell morphogenesis and NO signaling) (RCMNS) |
|  | *fldB* | P0ABY4 | 8.87E-08 | 1.80 | Flavodoxin 2 |
|  | *iadA* | P39377 | 9.31E-05 | 1.80 | Isoaspartyl dipeptidase (EC 3.4.19.-) |
|  | *ymdA* | P75917 | 2.65E-18 | 1.80 | Uncharacterized protein YmdA |
|  | *acnB* | P36683 | 3.74E-10 | 1.80 | Aconitate hydratase B (ACN) (Aconitase) (EC 4.2.1.3) ((2R,3S)-2-methylisocitrate dehydratase) ((2S,3R)-3-hydroxybutane-1,2,3-tricarboxylate dehydratase) (2-methyl-cis-aconitate hydratase) (EC 4.2.1.99) (Iron-responsive protein-like) (IRP-like) (RNA-binding protein) |
|  | *ycgR* | P76010 | 7.22E-38 | 1.79 | Flagellar brake protein YcgR (Cyclic di-GMP binding protein YcgR) |
|  | *moaE* | P30749 | 6.82E-13 | 1.79 | Molybdopterin synthase catalytic subunit (EC 2.8.1.12) (MPT synthase subunit 2) (Molybdenum cofactor biosynthesis protein E) (Molybdopterin-converting factor large subunit) (Molybdopterin-converting factor subunit 2) |
|  | *iraD* | P39375 | 2.64E-04 | 1.79 | Anti-adapter protein IraD |
|  | *eutS* | P63746 | 3.69E-09 | 1.78 | Ethanolamine utilization protein EutS |
|  | *fepB* | P0AEL6 | 1.05E-02 | 1.78 | Ferrienterobactin-binding periplasmic protein |
|  | *ygiN* | P0ADU2 | 3.81E-07 | 1.78 | Probable quinol monooxygenase YgiN (QuMo) (EC 1.-.-.-) |
|  | *yciI* | P0AB55 | 1.58E-15 | 1.78 | Protein YciI |
|  | *yehB* | P33341 | 1.39E-08 | 1.77 | Outer membrane usher protein YehB |
|  | *ymgD* | P0AB46 | 2.94E-06 | 1.77 | Uncharacterized protein YmgD |
|  | *uup* | P43672 | 3.14E-12 | 1.77 | ATP-binding protein Uup (EC 3.6.1.-) |
|  | *panE* | P0A9J4 | 5.07E-27 | 1.77 | 2-dehydropantoate 2-reductase (EC 1.1.1.169) (Ketopantoate reductase) (KPA reductase) (KPR) |
|  | *zitB* | P75757 | 3.66E-11 | 1.77 | Zinc transporter ZitB |
|  | *parE* | P20083 | 5.14E-08 | 1.76 | DNA topoisomerase 4 subunit B (EC 5.6.2.2) (Topoisomerase IV subunit B) |
|  | *yjcH* | P0AF54 | 1.03E-07 | 1.76 | Inner membrane protein YjcH |
|  | *xylG* | P37388 | 7.77E-08 | 1.76 | Xylose import ATP-binding protein XylG (EC 7.5.2.10) |
|  | *nanR* | P0A8W0 | 1.46E-32 | 1.76 | HTH-type transcriptional repressor NanR |
|  | *priC* | P23862 | 1.51E-21 | 1.75 | Primosomal replication protein N'' |
|  | *tdh* | P07913 | 3.55E-09 | 1.75 | L-threonine 3-dehydrogenase (TDH) (EC 1.1.1.103) (L-threonine dehydrogenase) |
|  | *zapE* | P64612 | 6.28E-05 | 1.75 | Cell division protein ZapE (Z ring-associated protein ZapE) |
|  | *ynjE* | P78067 | 2.18E-08 | 1.75 | Thiosulfate sulfurtransferase YnjE (EC 2.8.1.1) |
|  | *ycfT* | P75955 | 8.17E-45 | 1.75 | Inner membrane protein YcfT |
|  | *ybbJ* | P0AAS3 | 1.13E-36 | 1.75 | Inner membrane protein YbbJ |
|  | *mobA* | P32173 | 3.06E-20 | 1.75 | Molybdenum cofactor guanylyltransferase (MoCo guanylyltransferase) (EC 2.7.7.77) (GTP:molybdopterin guanylyltransferase) (Mo-MPT guanylyltransferase) (Molybdopterin guanylyltransferase) (Molybdopterin-guanine dinucleotide biosynthesis protein A) (Molybdopterin-guanine dinucleotide synthase) (MGD synthase) (Protein FA) |
|  | *yhdT* | P45566 | 6.92E-04 | 1.74 | Uncharacterized membrane protein YhdT |
|  | *yqjA* | P0AA63 | 2.93E-16 | 1.74 | Inner membrane protein YqjA |
|  | *tig* | P0A850 | 1.41E-10 | 1.74 | Trigger factor (TF) (EC 5.2.1.8) (PPIase) |
|  | *rdgB* | P52061 | 1.39E-08 | 1.74 | dITP/XTP pyrophosphatase (EC 3.6.1.66) (Deoxyribonucleoside triphosphate pyrophosphohydrolase) (Inosine triphosphate pyrophosphatase) (ITPase) (Non-canonical purine NTP pyrophosphatase) (Non-standard purine NTP pyrophosphatase) (Nucleoside-triphosphate diphosphatase) (Nucleoside-triphosphate pyrophosphatase) (NTPase) |
|  | *casB* | P76632 | 5.44E-04 | 1.74 | CRISPR system Cascade subunit CasB |
|  | *oxc* | P0AFI0 | 4.81E-04 | 1.73 | Oxalyl-CoA decarboxylase (EC 4.1.1.8) |
|  | *yigL* | P27848 | 5.59E-12 | 1.73 | Pyridoxal phosphate phosphatase YigL (EC 3.1.3.74) (PLP phosphatase) (Sugar phosphatase) (EC 3.1.3.23) |
|  | *pldA* | P0A921 | 2.23E-28 | 1.73 | Phospholipase A1 (EC 3.1.1.32) (EC 3.1.1.4) (Detergent-resistant phospholipase A) (DR-phospholipase A) (Outer membrane phospholipase A) (OM PLA) (OMPLA) (Phosphatidylcholine 1-acylhydrolase) |
|  | *deoC* | P0A6L0 | 8.35E-18 | 1.72 | Deoxyribose-phosphate aldolase (DERA) (EC 4.1.2.4) (2-deoxy-D-ribose 5-phosphate aldolase) (Phosphodeoxyriboaldolase) (Deoxyriboaldolase) |
|  | *clsC* | P75919 | 3.55E-11 | 1.72 | Cardiolipin synthase C (CL synthase) (EC 2.7.8.-) |
|  | *cyoD* | P0ABJ6 | 5.64E-07 | 1.72 | Cytochrome bo(3) ubiquinol oxidase subunit 4 (Cytochrome o ubiquinol oxidase subunit 4) (Cytochrome o subunit 4) (Oxidase bo(3) subunit 4) (Ubiquinol oxidase chain D) (Ubiquinol oxidase polypeptide IV) (Ubiquinol oxidase subunit 4) |
|  | *ybaT* | P77400 | 9.98E-34 | 1.71 | Inner membrane transport protein YbaT |
|  | *nudG* | P77788 | 2.62E-22 | 1.71 | CTP pyrophosphohydrolase (EC 3.6.1.65) |
|  | *cueR* | P0A9G4 | 1.78E-19 | 1.71 | HTH-type transcriptional regulator CueR (Copper efflux regulator) (Copper export regulator) |
|  | *betB* | P17445 | 1.10E-14 | 1.71 | Betaine aldehyde dehydrogenase (BADH) (EC 1.2.1.8) |
|  | *purU* | P37051 | 2.57E-26 | 1.71 | Formyltetrahydrofolate deformylase (EC 3.5.1.10) (Formyl-FH(4) hydrolase) |
|  | *cadC* | P23890 | 1.06E-09 | 1.71 | Transcriptional activator CadC |
|  | *pdeG* | P75995 | 3.03E-30 | 1.70 | Probable cyclic di-GMP phosphodiesterase PdeG (EC 3.1.4.52) |
|  | *pphA* | P55798 | 4.04E-26 | 1.69 | Serine/threonine-protein phosphatase 1 (EC 3.1.3.16) |
|  | *sbmA* | P0AFY6 | 7.67E-12 | 1.69 | Peptide antibiotic transporter SbmA |
|  | *tonB* | P02929 | 1.07E-15 | 1.68 | Protein TonB |
|  | *eptC* | P0CB39 | 2.16E-09 | 1.68 | Phosphoethanolamine transferase EptC (EC 2.7.-.-) |
|  | *pyrB* | P0A786 | 2.63E-10 | 1.67 | Aspartate carbamoyltransferase catalytic subunit (EC 2.1.3.2) (Aspartate transcarbamylase) (ATCase) |
|  | *kduI* | Q46938 | 1.18E-03 | 1.67 | 4-deoxy-L-threo-5-hexosulose-uronate ketol-isomerase (EC 5.3.1.17) (5-keto-4-deoxyuronate isomerase) (DKI isomerase) |
|  | *pnuC* | P0AFK2 | 8.08E-17 | 1.67 | Nicotinamide riboside transporter PnuC |
|  | *yajR* | P77726 | 4.58E-16 | 1.66 | Inner membrane transport protein YajR |
|  | *fliJ* | P52613 | 2.71E-04 | 1.66 | Flagellar FliJ protein |
|  | *chpS* | P08365 | 1.94E-29 | 1.66 | Antitoxin ChpS |
|  | *tdk* | P23331 | 6.33E-03 | 1.66 | Thymidine kinase (EC 2.7.1.21) |
|  | *rffH* | P61887 | 2.87E-21 | 1.66 | Glucose-1-phosphate thymidylyltransferase 2 (G1P-TT 2) (EC 2.7.7.24) (dTDP-glucose pyrophosphorylase 2) (dTDP-glucose synthase 2) |
|  | *entS* | P24077 | 1.46E-02 | 1.66 | Enterobactin exporter EntS (Protein p43) |
|  | *treB* | P36672 | 3.89E-08 | 1.66 | PTS system trehalose-specific EIIBC component (EIIBC-Tre) (EII-Tre) [Includes: Trehalose-specific phosphotransferase enzyme IIB component (EC 2.7.1.201) (PTS system trehalose-specific EIIB component); Trehalose permease IIC component (PTS system trehalose-specific EIIC component)] |
|  | *tktA* | P27302 | 1.76E-08 | 1.66 | Transketolase 1 (TK 1) (EC 2.2.1.1) |
|  | *gntU* | P0AC96 | 5.75E-03 | 1.65 | Low-affinity gluconate transporter (Gluconate permease) (Gnt-I system) |
|  | *asnS* | P0A8M0 | 2.25E-08 | 1.65 | Asparagine--tRNA ligase (EC 6.1.1.22) (Asparaginyl-tRNA synthetase) (AsnRS) |
|  | *ahpC* | P0AE08 | 4.36E-07 | 1.65 | Alkyl hydroperoxide reductase C (EC 1.11.1.26) (Alkyl hydroperoxide reductase protein C22) (Peroxiredoxin) (SCRP-23) (Sulfate starvation-induced protein 8) (SSI8) (Thioredoxin peroxidase) |
|  | *ssuB* | P0AAI1 | 2.84E-08 | 1.64 | Aliphatic sulfonates import ATP-binding protein SsuB (EC 7.6.2.14) |
|  | *eutN* | P0AEJ8 | 6.32E-04 | 1.64 | Ethanolamine catabolic microcompartment shell protein EutN (Ethanolamine utilization protein EutN) |
|  | *ydeO* | P76135 | 4.07E-04 | 1.64 | HTH-type transcriptional regulator YdeO |
|  | *prpD* | P77243 | 8.79E-19 | 1.63 | 2-methylcitrate dehydratase (2-MC dehydratase) (EC 4.2.1.79) ((2S,3S)-2-methylcitrate dehydratase) (Aconitate hydratase) (ACN) (Aconitase) (EC 4.2.1.3) |
|  | *yjgH* | P39332 | 1.12E-08 | 1.63 | RutC family protein YjgH |
|  | *uspA* | P0AED0 | 5.44E-11 | 1.63 | Universal stress protein A |
|  | *mukF* | P60293 | 1.86E-18 | 1.62 | Chromosome partition protein MukF (Protein KicB) |
|  | *yajL* | Q46948 | 6.48E-15 | 1.62 | Protein/nucleic acid deglycase 3 (EC 3.1.2.-) (EC 3.5.1.-) (EC 3.5.1.124) (Chaperone protein YajL) (Maillard deglycase) |
|  | *recT* | P33228 | 5.80E-17 | 1.62 | Protein RecT (P33) |
|  | *hda* | P69931 | 2.76E-05 | 1.62 | DnaA regulatory inactivator Hda (DnaA paralog) (Dp) |
|  | *frdC* | P0A8Q0 | 1.16E-29 | 1.61 | Fumarate reductase subunit C (Fumarate reductase 15 kDa hydrophobic protein) (Quinol-fumarate reductase subunit C) (QFR subunit C) |
|  | *ynjF* | P76226 | 1.38E-21 | 1.61 | Inner membrane protein YnjF |
|  | *clpS* | P0A8Q6 | 7.10E-11 | 1.60 | ATP-dependent Clp protease adapter protein ClpS |
|  | *zapB* | P0AF36 | 1.54E-04 | 1.60 | Cell division protein ZapB |
|  | *mcbA* | P0AAX6 | 4.45E-03 | 1.59 | Uncharacterized protein McbA (MqsR-controlled colanic acid and biofilm protein A) |
|  | *umuC* | P04152 | 1.05E-06 | 1.59 | Protein UmuC (DNA polymerase V) (Pol V) |
|  | *yifK* | P27837 | 1.29E-30 | 1.59 | Probable transport protein YifK |
|  | *frdD* | P0A8Q3 | 2.50E-27 | 1.59 | Fumarate reductase subunit D (Fumarate reductase 13 kDa hydrophobic protein) (Quinol-fumarate reductase subunit D) (QFR subunit D) |
|  | *metB* | P00935 | 5.32E-24 | 1.58 | Cystathionine gamma-synthase (CGS) (EC 2.5.1.48) (O-succinylhomoserine (thiol)-lyase) |
|  | *nanA* | P0A6L4 | 4.69E-11 | 1.58 | N-acetylneuraminate lyase (NAL) (Neu5Ac lyase) (EC 4.1.3.3) (N-acetylneuraminate pyruvate-lyase) (N-acetylneuraminic acid aldolase) (NALase) (Sialate lyase) (Sialic acid aldolase) (Sialic acid lyase) |
|  | *ybaN* | P0AAR5 | 5.94E-14 | 1.58 | Inner membrane protein YbaN |
|  | *pyrI* | P0A7F3 | 1.85E-09 | 1.58 | Aspartate carbamoyltransferase regulatory chain |
|  | *mobB* | P32125 | 2.04E-18 | 1.58 | Molybdopterin-guanine dinucleotide biosynthesis adapter protein (MGD biosynthesis adapter protein) (Molybdenum cofactor biosynthesis adapter protein) (Moco biosynthesis adapter protein) (Molybdopterin-guanine dinucleotide biosynthesis protein B) |
|  | *dgcT* | P75908 | 5.00E-39 | 1.57 | Probable diguanylate cyclase DgcT (DGC) (EC 2.7.7.65) |
|  | *fryC* | P77579 | 9.66E-20 | 1.57 | Fructose-like permease IIC component 1 (PTS system fructose-like EIIC component 1) |
|  | *yihD* | P0ADP9 | 1.06E-15 | 1.56 | Protein YihD |
|  | *ydjI* | P77704 | 1.10E-03 | 1.55 | Uncharacterized protein YdjI |
|  | *bdcR* | P39334 | 2.12E-26 | 1.55 | HTH-type transcriptional repressor BdcR |
|  | *fiu* | P75780 | 7.31E-09 | 1.55 | Catecholate siderophore receptor Fiu (Ferric iron uptake protein) (TonB-dependent receptor Fiu) |
|  | *hemX* | P09127 | 3.46E-32 | 1.55 | Protein HemX (ORF X) |
|  | *hslU* | P0A6H5 | 4.54E-10 | 1.55 | ATP-dependent protease ATPase subunit HslU (Heat shock protein HslU) (Unfoldase HslU) |
|  | *yfaL* | P45508 | 2.68E-14 | 1.55 | Probable autotransporter YfaL [Cleaved into: Probable secreted autotransporter protein YfaL; Probable autotransporter YfaL translocator] |
|  | *actP* | P32705 | 5.26E-07 | 1.55 | Cation/acetate symporter ActP (Acetate permease) (Acetate transporter ActP) |
|  | *pheP* | P24207 | 1.45E-06 | 1.55 | Phenylalanine-specific permease |
|  | *pyrF* | P08244 | 1.79E-08 | 1.54 | Orotidine 5'-phosphate decarboxylase (EC 4.1.1.23) (OMP decarboxylase) (OMPDCase) (OMPdecase) |
|  | *focA* | P0AC23 | 1.88E-05 | 1.54 | Probable formate transporter 1 (Formate channel 1) |
|  | *ghxQ* | Q46817 | 1.20E-07 | 1.54 | Guanine/hypoxanthine permease GhxQ |
|  | *ybjJ* | P75810 | 7.54E-12 | 1.54 | Inner membrane protein YbjJ |
|  | *rutC* | P0AFQ5 | 5.74E-07 | 1.54 | Putative aminoacrylate peracid reductase RutC (EC 1.-.-.-) |
|  | *phoU* | P0A9K7 | 3.26E-22 | 1.54 | Phosphate-specific transport system accessory protein PhoU (Pst system accessory protein PhoU) (Negative regulator of Pho regulon) |
|  | *mcrA* | P24200 | 3.57E-04 | 1.53 | 5-methylcytosine-specific restriction enzyme A (EC 3.1.21.-) (EcoKMcrA) |
|  | *yceF* | P0A729 | 2.56E-25 | 1.53 | 7-methyl-GTP pyrophosphatase (m(7)GTP pyrophosphatase) (EC 3.6.1.-) |
|  | *sohB* | P0AG14 | 1.43E-15 | 1.53 | Probable protease SohB (EC 3.4.21.-) |
|  | *ompG* | P76045 | 4.30E-36 | 1.52 | Outer membrane porin G (Outer membrane protein G) |
|  | *modA* | P37329 | 1.87E-13 | 1.52 | Molybdate-binding protein ModA (Molybdate/tungstate-binding protein ModA) |
|  | *mlaD* | P64604 | 8.24E-51 | 1.52 | Intermembrane phospholipid transport system binding protein MlaD |
|  | *iclR* | P16528 | 5.74E-13 | 1.52 | Transcriptional repressor IclR (Acetate operon repressor) |
|  | *dedA* | P0ABP6 | 6.68E-18 | 1.52 | Protein DedA (Protein DSG-1) |
|  | *clpA* | P0ABH9 | 1.24E-14 | 1.52 | ATP-dependent Clp protease ATP-binding subunit ClpA |
|  | *safA* | P76136 | 1.58E-03 | 1.51 | Two-component-system connector protein SafA (Regulatory protein b1500) (Sensor associating factor A) |
|  | *yciB* | P0A710 | 4.59E-13 | 1.51 | Probable intracellular septation protein A |
|  | *rtcA* | P46849 | 1.45E-07 | 1.51 | RNA 3'-terminal phosphate cyclase (RNA cyclase) (RNA-3'-phosphate cyclase) (EC 6.5.1.4) |
|  | *anmK* | P77570 | 1.86E-11 | 1.51 | Anhydro-N-acetylmuramic acid kinase (EC 2.7.1.170) (AnhMurNAc kinase) |
|  | *ytfP* | P0AE48 | 5.60E-65 | 1.50 | Gamma-glutamylcyclotransferase family protein YtfP |
|  | *btuF* | P37028 | 8.90E-07 | 1.50 | Vitamin B12-binding protein |
|  | *araF* | P02924 | 2.35E-30 | 1.50 | L-arabinose-binding periplasmic protein (ABP) |
|  | *xylH* | P0AGI4 | 1.03E-05 | 1.49 | Xylose transport system permease protein XylH |
|  | *fepD* | P23876 | 2.01E-02 | 1.49 | Ferric enterobactin transport system permease protein FepD |
|  | *ubiI* | P25535 | 1.41E-03 | 1.49 | 2-octaprenylphenol hydroxylase (EC 1.14.13.240) (2-polyprenylphenol 6-hydroxylase) |
|  | *rhmD* | P77215 | 1.11E-28 | 1.49 | L-rhamnonate dehydratase (RhamD) (EC 4.2.1.90) |
|  | *hisH* | P60595 | 1.65E-10 | 1.49 | Imidazole glycerol phosphate synthase subunit HisH (EC 4.3.2.10) (IGP synthase glutaminase subunit) (EC 3.5.1.2) (IGP synthase subunit HisH) (ImGP synthase subunit HisH) (IGPS subunit HisH) |
|  | *ydjJ* | P77280 | 3.40E-03 | 1.49 | Uncharacterized zinc-type alcohol dehydrogenase-like protein YdjJ (EC 1.-.-.-) |
|  | *yihT* | P32141 | 2.00E-04 | 1.49 | Sulfofructosephosphate aldolase (SFP aldolase) (EC 4.1.2.57) |
|  | *tas* | P0A9T4 | 2.80E-03 | 1.49 | Protein tas |
|  | *dinG* | P27296 | 1.15E-10 | 1.49 | ATP-dependent DNA helicase DinG (EC 3.6.4.12) |
|  | *yceI* | P0A8X2 | 7.82E-43 | 1.49 | Protein YceI |
|  | *pstB* | P0AAH0 | 4.38E-27 | 1.48 | Phosphate import ATP-binding protein PstB (EC 7.3.2.1) (ABC phosphate transporter) (Phosphate-transporting ATPase) |
|  | *rluC* | P0AA39 | 9.11E-19 | 1.48 | Ribosomal large subunit pseudouridine synthase C (EC 5.4.99.24) (23S rRNA pseudouridine(955/2504/2580) synthase) (rRNA pseudouridylate synthase C) (rRNA-uridine isomerase C) |
|  | *clpP* | P0A6G7 | 1.48E-16 | 1.48 | ATP-dependent Clp protease proteolytic subunit (EC 3.4.21.92) (Caseinolytic protease) (Endopeptidase Clp) (Heat shock protein F21.5) (Protease Ti) |
|  | *hemY* | P0ACB7 | 9.02E-14 | 1.48 | Protein HemY |
|  | *pabC* | P28305 | 7.77E-13 | 1.48 | Aminodeoxychorismate lyase (EC 4.1.3.38) (4-amino-4-deoxychorismate lyase) (ADC lyase) (ADCL) |
|  | *mcrB* | P15005 | 1.95E-14 | 1.48 | 5-methylcytosine-specific restriction enzyme B (EC 3.1.21.-) (EcoKMcrBC) |
|  | *barA* | P0AEC5 | 2.75E-26 | 1.48 | Signal transduction histidine-protein kinase BarA (EC 2.7.13.3) |
|  | *nagK* | P75959 | 4.12E-10 | 1.47 | N-acetyl-D-glucosamine kinase (EC 2.7.1.59) (GlcNAc kinase) |
|  | *galT* | P09148 | 1.06E-05 | 1.47 | Galactose-1-phosphate uridylyltransferase (Gal-1-P uridylyltransferase) (EC 2.7.7.12) (UDP-glucose--hexose-1-phosphate uridylyltransferase) |
|  | *lapB* | P0AB58 | 3.64E-07 | 1.46 | Lipopolysaccharide assembly protein B (Lipopolysaccharide regulatory protein) |
|  | *yjjG* | P0A8Y1 | 2.10E-09 | 1.46 | Pyrimidine 5'-nucleotidase YjjG (EC 3.1.3.5) (House-cleaning nucleotidase) (Non-canonical pyrimidine nucleotide phosphatase) (Nucleoside 5'-monophosphate phosphohydrolase) (dUMP phosphatase) |
|  | *yjjJ* | P39410 | 3.74E-36 | 1.46 | Toxin YjjJ (Putative DNA-binding transcriptional regulator YjjJ) (Putative serine/threonine kinase toxin YjjJ) (EC 2.-.-.-) |
|  | *cmoM* | P36566 | 4.58E-14 | 1.46 | tRNA 5-carboxymethoxyuridine methyltransferase (EC 2.1.1.-) (cmo5U methyltransferase) |
|  | *yfbR* | P76491 | 6.62E-11 | 1.45 | 5'-deoxynucleotidase YfbR (EC 3.1.3.89) (5'-deoxyribonucleotidase) (Nucleoside 5'-monophosphate phosphohydrolase) |
|  | *dcuS* | P0AEC8 | 3.46E-05 | 1.45 | Sensor histidine kinase DcuS (EC 2.7.13.3) (Fumarate sensor) |
|  | *murG* | P17443 | 3.93E-03 | 1.45 | UDP-N-acetylglucosamine--N-acetylmuramyl-(pentapeptide) pyrophosphoryl-undecaprenol N-acetylglucosamine transferase (EC 2.4.1.227) (Undecaprenyl-PP-MurNAc-pentapeptide-UDPGlcNAc GlcNAc transferase) |
|  | *bglA* | Q46829 | 2.32E-12 | 1.45 | 6-phospho-beta-glucosidase BglA (EC 3.2.1.86) (Phospho-beta-glucosidase A) |
|  | *apt* | P69503 | 2.92E-13 | 1.44 | Adenine phosphoribosyltransferase (APRT) (EC 2.4.2.7) |
|  | *tdcF* | P0AGL2 | 3.89E-03 | 1.44 | Putative reactive intermediate deaminase TdcF (EC 3.5.4.-) |
|  | *fetB* | P77307 | 2.92E-15 | 1.44 | Probable iron export permease protein FetB |
|  | *ydhV* | P76192 | 8.16E-14 | 1.43 | Uncharacterized oxidoreductase YdhV (EC 1.-.-.-) |
|  | *ubiK* | Q46868 | 3.63E-06 | 1.43 | Ubiquinone biosynthesis accessory factor UbiK |
|  | *dgcJ* | P76237 | 5.07E-40 | 1.43 | Probable diguanylate cyclase DgcJ (DGC) (EC 2.7.7.65) |
|  | *glpD* | P13035 | 1.92E-03 | 1.42 | Aerobic glycerol-3-phosphate dehydrogenase (EC 1.1.5.3) |
|  | *eamA* | P31125 | 1.57E-13 | 1.42 | Probable amino-acid metabolite efflux pump |
|  | *cyoE* | P0AEA5 | 6.08E-15 | 1.42 | Protoheme IX farnesyltransferase (EC 2.5.1.141) (Heme B farnesyltransferase) (Heme O synthase) |
|  | *csgC* | P52107 | 3.41E-06 | 1.42 | Curli assembly protein CsgC |
|  | *rlmC* | P75817 | 1.63E-06 | 1.42 | 23S rRNA (uracil(747)-C(5))-methyltransferase RlmC (EC 2.1.1.189) (23S rRNA(m5U747)-methyltransferase) |
|  | *gltI* | P37902 | 6.18E-07 | 1.42 | Glutamate/aspartate import solute-binding protein |
|  | *tabA* | P0AF96 | 1.27E-14 | 1.41 | Toxin-antitoxin biofilm protein TabA |
|  | *ppsR* | P0A8A4 | 1.71E-13 | 1.41 | Phosphoenolpyruvate synthase regulatory protein (PEP synthase regulatory protein) (PSRP) (EC 2.7.11.33) (EC 2.7.4.28) (Pyruvate, water dikinase regulatory protein) |
|  | *tpiA* | P0A858 | 2.13E-04 | 1.41 | Triosephosphate isomerase (TIM) (TPI) (EC 5.3.1.1) (Triose-phosphate isomerase) |
|  | *yfgD* | P76569 | 5.67E-05 | 1.41 | Uncharacterized protein YfgD |
|  | *mscK* | P77338 | 4.76E-29 | 1.41 | Mechanosensitive channel MscK (Potassium efflux system KefA) |
|  | *hslV* | P0A7B8 | 7.69E-07 | 1.41 | ATP-dependent protease subunit HslV (EC 3.4.25.2) (Heat shock protein HslV) |
|  | *nupX* | P33021 | 1.19E-03 | 1.41 | Putative nucleoside permease NupX |
|  | *narH* | P11349 | 1.09E-04 | 1.41 | Respiratory nitrate reductase 1 beta chain (EC 1.7.5.1) (Nitrate reductase A subunit beta) (Quinol-nitrate oxidoreductase subunit beta) |
|  | *ydhF* | P76187 | 1.29E-08 | 1.40 | Oxidoreductase YdhF (EC 1.-.-.-) |
|  | *ompF* | P02931 | 4.93E-43 | 1.40 | Outer membrane porin F (Outer membrane protein 1A) (Outer membrane protein B) (Outer membrane protein F) (Outer membrane protein IA) (Porin OmpF) |
|  | *ilvC* | P05793 | 4.05E-44 | 1.40 | Ketol-acid reductoisomerase (NADP(+)) (KARI) (EC 1.1.1.86) (Acetohydroxy-acid isomeroreductase) (AHIR) (Alpha-keto-beta-hydroxylacyl reductoisomerase) (Ketol-acid reductoisomerase type 2) (Ketol-acid reductoisomerase type II) |
|  | *yggF* | P21437 | 1.07E-05 | 1.40 | Fructose-1,6-bisphosphatase 2 class 2 (FBPase 2 class 2) (EC 3.1.3.11) (D-fructose-1,6-bisphosphate 1-phosphohydrolase 2 class 2) |
|  | *metJ* | P0A8U6 | 2.44E-09 | 1.40 | Met repressor (Met regulon regulatory protein MetJ) |
|  | *dsbB* | P0A6M2 | 2.63E-06 | 1.39 | Disulfide bond formation protein B (Disulfide oxidoreductase) |
|  | *napC* | P0ABL5 | 3.86E-05 | 1.39 | Cytochrome c-type protein NapC |
|  | *moaA* | P30745 | 2.70E-10 | 1.39 | GTP 3',8-cyclase (EC 4.1.99.22) (Molybdenum cofactor biosynthesis protein A) |
|  | *higB* | P64578 | 6.19E-03 | 1.39 | mRNA interferase toxin HigB (EC 3.1.-.-) (Endoribonuclease HigB) (Toxin HigB) |
|  | *menA* | P32166 | 3.19E-07 | 1.38 | 1,4-dihydroxy-2-naphthoate octaprenyltransferase (DHNA-octaprenyltransferase) (EC 2.5.1.74) |
|  | *araH* | P0AE26 | 2.09E-05 | 1.38 | L-arabinose transport system permease protein AraH |
|  | *orn* | P0A784 | 1.06E-11 | 1.38 | Oligoribonuclease (EC 3.1.-.-) |
|  | *pdeC* | P32701 | 1.75E-12 | 1.38 | Probable cyclic di-GMP phosphodiesterase PdeC (EC 3.1.4.52) |
|  | *uspB* | P0A8S5 | 1.31E-16 | 1.38 | Universal stress protein B |
|  | *yphF* | P77269 | 8.42E-05 | 1.38 | ABC transporter periplasmic-binding protein YphF |
|  | *ymdB* | P0A8D6 | 5.39E-11 | 1.37 | O-acetyl-ADP-ribose deacetylase (EC 3.1.1.106) (Regulator of RNase III activity) |
|  | *sgbH* | P37678 | 2.57E-04 | 1.37 | 3-keto-L-gulonate-6-phosphate decarboxylase SgbH (KGPDC) (EC 4.1.1.85) (3-dehydro-L-gulonate-6-phosphate decarboxylase) |
|  | *osmB* | P0ADA7 | 9.96E-07 | 1.37 | Osmotically-inducible lipoprotein B |
|  | *higA* | P67701 | 9.28E-03 | 1.37 | Antitoxin HigA |
|  | *menE* | P37353 | 7.31E-03 | 1.37 | 2-succinylbenzoate--CoA ligase (EC 6.2.1.26) (o-succinylbenzoyl-CoA synthetase) (OSB-CoA synthetase) |
|  | *mglC* | P23200 | 7.24E-35 | 1.36 | Galactoside transport system permease protein MglC |
|  | *tsaB* | P76256 | 1.08E-13 | 1.36 | tRNA threonylcarbamoyladenosine biosynthesis protein TsaB (t(6)A37 threonylcarbamoyladenosine biosynthesis protein TsaB) |
|  | *bcsB* | P37652 | 2.39E-12 | 1.36 | Cyclic di-GMP-binding protein (Cellulose synthase regulatory subunit) |
|  | *arnD* | P76472 | 3.62E-11 | 1.36 | Probable 4-deoxy-4-formamido-L-arabinose-phosphoundecaprenol deformylase ArnD (EC 3.5.1.n3) |
|  | *hypC* | P0AAM3 | 2.54E-07 | 1.35 | Hydrogenase maturation factor HypC (Chaperone-type protein HypC) (Hydrogenase accessory chaperone HypC) |
|  | *yhhW* | P46852 | 4.09E-16 | 1.35 | Quercetin 2,3-dioxygenase (Quercetinase) (EC 1.13.11.24) (Pirin-like protein YhhW) |
|  | *zupT* | P0A8H3 | 1.89E-04 | 1.35 | Zinc transporter ZupT |
|  | *sdaA* | P16095 | 1.67E-07 | 1.35 | L-serine dehydratase 1 (SDH 1) (EC 4.3.1.17) (L-serine deaminase 1) (L-SD1) |
|  | *ytfQ* | P39325 | 4.71E-07 | 1.34 | Galactofuranose-binding protein YtfQ |
|  | *casD* | Q46898 | 1.97E-03 | 1.34 | CRISPR system Cascade subunit CasD |
|  | *helD* | P15038 | 5.32E-05 | 1.34 | DNA helicase IV (EC 3.6.4.12) (75 kDa helicase) |
|  | *ubiF* | P75728 | 2.01E-48 | 1.34 | 3-demethoxyubiquinol 3-hydroxylase (EC 1.14.99.60) (2-octaprenyl-3-methyl-6-methoxy-1,4-benzoquinol hydroxylase) |
|  | *yjiK* | P39382 | 4.11E-12 | 1.34 | Uncharacterized protein YjiK |
|  | *acnA* | P25516 | 4.25E-15 | 1.33 | Aconitate hydratase A (ACN) (Aconitase) (EC 4.2.1.3) (Iron-responsive protein-like) (IRP-like) (RNA-binding protein) (Stationary phase enzyme) |
|  | *ppx* | P0AFL6 | 2.71E-06 | 1.33 | Exopolyphosphatase (ExopolyPase) (EC 3.6.1.11) (Metaphosphatase) |
|  | *yqcG* | C1P612 | 3.25E-07 | 1.33 | Uncharacterized protein YqcG |
|  | *yqiJ* | P76657 | 1.85E-14 | 1.33 | Inner membrane protein YqiJ |
|  | *dinI* | P0ABR1 | 6.75E-08 | 1.33 | DNA damage-inducible protein I |
|  | *yciH* | P08245 | 2.51E-08 | 1.32 | Uncharacterized protein YciH |
|  | *truC* | P0AA41 | 5.13E-23 | 1.32 | tRNA pseudouridine synthase C (EC 5.4.99.26) (tRNA pseudouridine(65) synthase) (tRNA pseudouridylate synthase C) (tRNA-uridine isomerase C) |
|  | *glgC* | P0A6V1 | 1.96E-26 | 1.32 | Glucose-1-phosphate adenylyltransferase (EC 2.7.7.27) (ADP-glucose pyrophosphorylase) (ADPGlc PPase) (ADP-glucose synthase) |
|  | *xseA* | P04994 | 1.58E-09 | 1.32 | Exodeoxyribonuclease 7 large subunit (EC 3.1.11.6) (Exodeoxyribonuclease VII large subunit) (Exonuclease VII large subunit) |
|  | *treR* | P36673 | 2.10E-04 | 1.31 | HTH-type transcriptional regulator TreR (Trehalose operon repressor) |
|  | *cadA* | P0A9H3 | 2.12E-02 | 1.31 | Inducible lysine decarboxylase (LDCI) (EC 4.1.1.18) |
|  | *yhiM* | P37630 | 1.31E-06 | 1.30 | Inner membrane protein YhiM |
|  | *treA* | P13482 | 2.17E-34 | 1.29 | Periplasmic trehalase (EC 3.2.1.28) (Alpha,alpha-trehalase) (Alpha,alpha-trehalose glucohydrolase) (Tre37A) |
|  | *ylaC* | P0AAS0 | 1.70E-11 | 1.29 | Inner membrane protein YlaC |
|  | *hypD* | P24192 | 5.19E-06 | 1.29 | Hydrogenase maturation factor HypD (Hydrogenase isoenzymes formation protein HypD) |
|  | *casA* | Q46901 | 3.42E-03 | 1.29 | CRISPR system Cascade subunit CasA (CRISPR type I-E/Ecoli-associated protein CasA/Cse1) (CRISPR-associated protein CasA/Cse1) |
|  | *fruB* | P69811 | 1.42E-03 | 1.29 | Multiphosphoryl transfer protein (MTP) (Diphosphoryl transfer protein) (DTP) (Phosphotransferase FPr protein) (Pseudo-HPr) [Includes: Phosphocarrier protein HPr (Protein H); PTS system fructose-specific EIIA component (EIIA-Fru) (EIII-Fru) (Fructose-specific phosphotransferase enzyme IIA component)] |
|  | *murC* | P17952 | 2.26E-04 | 1.29 | UDP-N-acetylmuramate--L-alanine ligase (EC 6.3.2.8) (UDP-N-acetylmuramoyl-L-alanine synthetase) |
|  | *cpoB* | P45955 | 3.40E-03 | 1.29 | Cell division coordinator CpoB |
|  | *allA* | P77731 | 1.86E-03 | 1.29 | Ureidoglycolate lyase (EC 4.3.2.3) (Ureidoglycolatase) (Ureidoglycolate hydrolase) |
|  | *htrL* | P25666 | 2.22E-08 | 1.29 | Protein HtrL |
|  | *rhaA* | P32170 | 7.72E-07 | 1.29 | L-rhamnose isomerase (EC 5.3.1.14) |
|  | *nlpE* | P40710 | 9.13E-18 | 1.29 | Lipoprotein NlpE (Copper homeostasis protein CutF) |
|  | *arnA* | P77398 | 1.27E-07 | 1.28 | Bifunctional polymyxin resistance protein ArnA (Polymyxin resistance protein PmrI) [Includes: UDP-4-amino-4-deoxy-L-arabinose formyltransferase (EC 2.1.2.13) (ArnAFT) (UDP-L-Ara4N formyltransferase); UDP-glucuronic acid oxidase, UDP-4-keto-hexauronic acid decarboxylating (EC 1.1.1.305) (ArnADH) (UDP-GlcUA decarboxylase) (UDP-glucuronic acid dehydrogenase)] |
|  | *pepA* | P68767 | 2.84E-07 | 1.28 | Cytosol aminopeptidase (EC 3.4.11.1) (Aminopeptidase A/I) (Leucine aminopeptidase) (LAP) (EC 3.4.11.10) (Leucyl aminopeptidase) |
|  | *elaA* | P0AEH3 | 2.71E-02 | 1.28 | Protein ElaA |
|  | *truB* | P60340 | 1.39E-09 | 1.28 | tRNA pseudouridine synthase B (EC 5.4.99.25) (Protein P35) (tRNA pseudouridine(55) synthase) (Psi55 synthase) (tRNA pseudouridylate synthase) (tRNA-uridine isomerase) |
|  | *caiD* | P31551 | 6.68E-08 | 1.28 | Carnitinyl-CoA dehydratase (EC 4.2.1.149) (Crotonobetainyl-CoA hydratase) |
|  | *ppsA* | P23538 | 2.85E-08 | 1.28 | Phosphoenolpyruvate synthase (PEP synthase) (EC 2.7.9.2) (Pyruvate, water dikinase) |
|  | *hemF* | P36553 | 3.61E-05 | 1.27 | Oxygen-dependent coproporphyrinogen-III oxidase (CPO) (Coprogen oxidase) (Coproporphyrinogenase) (EC 1.3.3.3) |
|  | *yfcR* | P76501 | 4.06E-05 | 1.27 | Uncharacterized fimbrial-like protein YfcR |
|  | *ydgC* | P0ACX0 | 1.45E-02 | 1.27 | Inner membrane protein YdgC |
|  | *queE* | P64554 | 2.60E-08 | 1.27 | 7-carboxy-7-deazaguanine synthase (CDG synthase) (EC 4.3.99.3) (Queuosine biosynthesis protein QueE) |
|  | *bolA* | P0ABE2 | 2.26E-08 | 1.27 | DNA-binding transcriptional regulator BolA |
|  | *thiB* | P31550 | 2.87E-03 | 1.27 | Thiamine-binding periplasmic protein |
|  | *bdcA* | P39333 | 3.64E-09 | 1.26 | Cyclic-di-GMP-binding biofilm dispersal mediator protein |
|  | *hisQ* | P52094 | 6.18E-08 | 1.26 | Histidine transport system permease protein HisQ |
|  | *glpK* | P0A6F3 | 1.96E-35 | 1.26 | Glycerol kinase (EC 2.7.1.30) (ATP:glycerol 3-phosphotransferase) (Glycerokinase) (GK) |
|  | *trmO* | P28634 | 9.14E-06 | 1.25 | tRNA (adenine(37)-N6)-methyltransferase (EC 2.1.1.-) (tRNA (m6t6A37) methyltransferase) (tRNA methyltransferase O) |
|  | *livJ* | P0AD96 | 1.40E-28 | 1.25 | Leu/Ile/Val-binding protein (LIV-BP) |
|  | *amiD* | P75820 | 2.50E-03 | 1.25 | N-acetylmuramoyl-L-alanine amidase AmiD (EC 3.5.1.28) |
|  | *scpC* | P52043 | 1.96E-14 | 1.25 | Propionyl-CoA:succinate CoA transferase (EC 2.8.3.-) |
|  | *rluF* | P32684 | 1.13E-03 | 1.25 | Dual-specificity RNA pseudouridine synthase RluF (EC 5.4.99.-) (EC 5.4.99.21) (23S rRNA pseudouridine(2604) synthase) (Ribosomal large subunit pseudouridine synthase F) (rRNA pseudouridylate synthase F) (rRNA-uridine isomerase F) (tRNA(Tyr) pseudouridine(35) synthase) |
|  | *pdeD* | P76261 | 7.16E-07 | 1.25 | Probable cyclic di-GMP phosphodiesterase PdeD (EC 3.1.4.52) |
|  | *nrdR* | P0A8D0 | 1.47E-13 | 1.25 | Transcriptional repressor NrdR |
|  | *ampC* | P00811 | 1.71E-09 | 1.24 | Beta-lactamase (EC 3.5.2.6) (Cephalosporinase) |
|  | *argF* | P06960 | 2.84E-05 | 1.24 | Ornithine carbamoyltransferase subunit F (OTCase-2) (EC 2.1.3.3) |
|  | *nimR* | P76241 | 1.36E-06 | 1.24 | HTH-type transcriptional regulator NimR (Regulator of nimT) |
|  | *gsiB* | P75797 | 4.78E-03 | 1.24 | Glutathione-binding protein GsiB |
|  | *ulaG* | P39300 | 2.94E-09 | 1.24 | Probable L-ascorbate-6-phosphate lactonase UlaG (EC 3.1.1.-) (L-ascorbate utilization protein G) |
|  | *csgD* | P52106 | 4.81E-05 | 1.24 | CsgBAC operon transcriptional regulatory protein |
|  | *aroE* | P15770 | 1.18E-22 | 1.23 | Shikimate dehydrogenase (NADP(+)) (SD) (SDH) (EC 1.1.1.25) |
|  | *yebV* | P64503 | 1.10E-08 | 1.23 | Uncharacterized protein YebV |
|  | *rseA* | P0AFX7 | 1.58E-04 | 1.23 | Anti-sigma-E factor RseA (Regulator of SigE) (Sigma-E anti-sigma factor RseA) (Sigma-E factor negative regulatory protein) |
|  | *cdaR* | P37047 | 3.82E-20 | 1.22 | Carbohydrate diacid regulator (Sugar diacid regulator) |
|  | *glpE* | P0A6V5 | 9.74E-04 | 1.22 | Thiosulfate sulfurtransferase GlpE (EC 2.8.1.1) |
|  | *rlmF* | P75782 | 4.00E-02 | 1.22 | Ribosomal RNA large subunit methyltransferase F (EC 2.1.1.181) (23S rRNA mA1618 methyltransferase) (rRNA adenine N-6-methyltransferase) |
|  | *secE* | P0AG96 | 2.09E-10 | 1.22 | Protein translocase subunit SecE |
|  | *pflA* | P0A9N4 | 3.66E-04 | 1.22 | Pyruvate formate-lyase 1-activating enzyme (EC 1.97.1.4) (Formate-C-acetyltransferase-activating enzyme 1) (PFL-activating enzyme 1) |
|  | *lolC* | P0ADC3 | 3.35E-14 | 1.22 | Lipoprotein-releasing system transmembrane protein LolC |
|  | *ybiR* | P75788 | 1.68E-18 | 1.22 | Inner membrane protein YbiR |
|  | *yjjI* | P37342 | 4.87E-21 | 1.22 | Uncharacterized protein YjjI |
|  | *yedJ* | P46144 | 3.62E-05 | 1.22 | Uncharacterized protein YedJ |
|  | *gspK* | P45762 | 3.26E-06 | 1.22 | Putative type II secretion system protein K (T2SS protein K) (Putative general secretion pathway protein K) |
|  | *prpC* | P31660 | 8.55E-04 | 1.22 | 2-methylcitrate synthase (2-MCS) (MCS) (EC 2.3.3.5) ((2S,3S)-2-methylcitrate synthase) (Citrate synthase) (EC 2.3.3.16) |
|  | *citX* | P0A6G5 | 3.31E-03 | 1.22 | Apo-citrate lyase phosphoribosyl-dephospho-CoA transferase (EC 2.7.7.61) (Apo-ACP nucleodityltransferase) (Holo-ACP synthase) (Holo-citrate lyase synthase) |
|  | *pdeB* | P77473 | 3.62E-17 | 1.21 | Probable cyclic di-GMP phosphodiesterase PdeB (EC 3.1.4.52) |
|  | *ridA* | P0AF93 | 1.00E-08 | 1.20 | 2-iminobutanoate/2-iminopropanoate deaminase (EC 3.5.99.10) (Enamine/imine deaminase) |
|  | *rsmC* | P39406 | 2.30E-06 | 1.20 | Ribosomal RNA small subunit methyltransferase C (EC 2.1.1.172) (16S rRNA m2G1207 methyltransferase) (rRNA (guanine-N(2)-)-methyltransferase RsmC) |
|  | *miaA* | P16384 | 8.12E-04 | 1.20 | tRNA dimethylallyltransferase (EC 2.5.1.75) (Dimethylallyl diphosphate:tRNA dimethylallyltransferase) (DMAPP:tRNA dimethylallyltransferase) (DMATase) (Isopentenyl-diphosphate:tRNA isopentenyltransferase) (IPP transferase) (IPPT) (IPTase) |
|  | *ycaD* | P21503 | 1.65E-03 | 1.20 | Uncharacterized MFS-type transporter YcaD |
|  | *eno* | P0A6P9 | 1.46E-13 | 1.20 | Enolase (EC 4.2.1.11) (2-phospho-D-glycerate hydro-lyase) (2-phosphoglycerate dehydratase) |
|  | *surA* | P0ABZ6 | 1.26E-14 | 1.20 | Chaperone SurA (Peptidyl-prolyl cis-trans isomerase SurA) (PPIase SurA) (EC 5.2.1.8) (Rotamase SurA) (Survival protein A) |
|  | *fieF* | P69380 | 1.12E-18 | 1.19 | Ferrous-iron efflux pump FieF |
|  | *pdeR* | P77334 | 1.82E-06 | 1.19 | Cyclic di-GMP phosphodiesterase PdeR (EC 3.1.4.52) |
|  | *setB* | P33026 | 1.02E-04 | 1.19 | Sugar efflux transporter B |
|  | *yihG* | P32129 | 2.84E-05 | 1.19 | Probable acyltransferase YihG (EC 2.3.-.-) |
|  | *lrp* | P0ACJ0 | 6.89E-05 | 1.19 | Leucine-responsive regulatory protein |
|  | *tufB* | P0CE48 | 1.56E-13 | 1.19 | Elongation factor Tu 2 (EF-Tu 2) (Bacteriophage Q beta RNA-directed RNA polymerase subunit III) (P-43) |
|  | *yeaD* | P39173 | 5.51E-03 | 1.19 | Putative glucose-6-phosphate 1-epimerase (EC 5.1.3.15) (Putative D-hexose-6-phosphate mutarotase) (Unknown protein from 2D-page spots T26/PR37) |
|  | *ghoT* | P64646 | 4.60E-09 | 1.19 | Toxin GhoT |
|  | *ydcU* | P77156 | 1.82E-05 | 1.19 | Inner membrane ABC transporter permease protein YdcU |
|  | *yfcS* | P77599 | 1.46E-04 | 1.18 | Probable fimbrial chaperone YfcS |
|  | *yihS* | P32140 | 5.06E-15 | 1.18 | Sulfoquinovose isomerase (SQ isomerase) (EC 5.3.1.31) |
|  | *cirA* | P17315 | 6.54E-04 | 1.18 | Colicin I receptor |
|  | *yceD* | P0AB28 | 3.67E-36 | 1.17 | Large ribosomal RNA subunit accumulation protein YceD (23S rRNA accumulation protein YceD) (G30K) |
|  | *srkA* | P0C0K3 | 1.06E-30 | 1.17 | Stress response kinase A (EC 2.7.11.1) (Serine/threonine protein kinase YihE) (Serine/threonine-protein kinase SrkA) |
|  | *accD* | P0A9Q5 | 5.69E-12 | 1.17 | Acetyl-coenzyme A carboxylase carboxyl transferase subunit beta (ACCase subunit beta) (Acetyl-CoA carboxylase carboxyltransferase subunit beta) (EC 2.1.3.15) |
|  | *nagA* | P0AF18 | 2.41E-16 | 1.17 | N-acetylglucosamine-6-phosphate deacetylase (GlcNAc 6-P deacetylase) (EC 3.5.1.25) |
|  | *hdhA* | P0AET8 | 1.03E-10 | 1.17 | 7alpha-hydroxysteroid dehydrogenase (7alpha-HSDH) (EC 1.1.1.159) (NAD-dependent 7alpha-hydroxysteroid dehydrogenase) |
|  | *sodA* | P00448 | 9.90E-20 | 1.17 | Superoxide dismutase [Mn] (EC 1.15.1.1) (MnSOD) |
|  | *truA* | P07649 | 2.07E-11 | 1.16 | tRNA pseudouridine synthase A (EC 5.4.99.12) (tRNA pseudouridine(38-40) synthase) (tRNA pseudouridylate synthase I) (PSU-I) (tRNA-uridine isomerase I) |
|  | *dnaN* | P0A988 | 5.38E-06 | 1.16 | Beta sliding clamp (Beta clamp) (Sliding clamp) (Beta-clamp processivity factor) (DNA polymerase III beta sliding clamp subunit) |
|  | *frdB* | P0AC47 | 9.87E-21 | 1.16 | Fumarate reductase iron-sulfur subunit (EC 1.3.5.4) (Quinol-fumarate reductase iron-sulfur subunit) (QFR iron-sulfur subunit) |
|  | *lptD* | P31554 | 2.65E-09 | 1.16 | LPS-assembly protein LptD (Organic solvent tolerance protein) |
|  | *yoaA* | P76257 | 1.37E-14 | 1.16 | Probable ATP-dependent DNA helicase YoaA (EC 3.6.4.12) |
|  | *rhaB* | P32171 | 8.88E-06 | 1.16 | L-Rhamnulokinase (RhaB) (RhuK) (EC 2.7.1.5) (ATP:L-rhamnulose phosphotransferase) (L-rhamnulose 1-kinase) (Rhamnulose kinase) |
|  | *menB* | P0ABU0 | 3.65E-02 | 1.16 | 1,4-dihydroxy-2-naphthoyl-CoA synthase (DHNA-CoA synthase) (EC 4.1.3.36) |
|  | *ygiS* | Q46863 | 5.93E-09 | 1.16 | Probable deoxycholate-binding periplasmic protein YgiS |
|  | *phoA* | P00634 | 4.65E-03 | 1.15 | Alkaline phosphatase (APase) (EC 3.1.3.1) |
|  | *rsgA* | P39286 | 3.71E-12 | 1.15 | Small ribosomal subunit biogenesis GTPase RsgA (EC 3.6.1.-) |
|  | *gpt* | P0A9M5 | 1.56E-03 | 1.15 | Xanthine-guanine phosphoribosyltransferase (XGPRT) (EC 2.4.2.-) (EC 2.4.2.22) (Xanthine phosphoribosyltransferase) |
|  | *mtlR* | P0AF10 | 9.14E-15 | 1.15 | Mannitol operon repressor (Mannitol repressor protein) |
|  | *hicA* | P76106 | 4.65E-04 | 1.15 | Probable mRNA interferase toxin HicA (EC 3.1.-.-) (Endoribonuclease HicA) (Toxin HicA) |
|  | *rimI* | P0A944 | 5.45E-19 | 1.14 | [Ribosomal protein S18]-alanine N-acetyltransferase (EC 2.3.1.266) (KAT) (Peptidyl-lysine N-acetyltransferase) (EC 2.3.1.-) |
|  | *betA* | P17444 | 3.10E-06 | 1.14 | Oxygen-dependent choline dehydrogenase (CDH) (CHD) (EC 1.1.99.1) (Betaine aldehyde dehydrogenase) (BADH) (EC 1.2.1.8) |
|  | *yedI* | P46125 | 1.47E-06 | 1.14 | Inner membrane protein YedI |
|  | *gcvP* | P33195 | 4.17E-08 | 1.14 | Glycine dehydrogenase (decarboxylating) (EC 1.4.4.2) (Glycine cleavage system P-protein) (Glycine decarboxylase) (Glycine dehydrogenase (aminomethyl-transferring)) |
|  | *rsmG* | P0A6U5 | 6.38E-04 | 1.14 | Ribosomal RNA small subunit methyltransferase G (EC 2.1.1.170) (16S rRNA 7-methylguanosine methyltransferase) (16S rRNA m7G methyltransferase) (Glucose-inhibited division protein B) |
|  | *rhlE* | P25888 | 1.98E-05 | 1.14 | ATP-dependent RNA helicase RhlE (EC 3.6.4.13) |
|  | *preA* | P25889 | 4.83E-14 | 1.14 | NAD-dependent dihydropyrimidine dehydrogenase subunit PreA (DPD) (EC 1.3.1.1) (Dihydrothymine dehydrogenase) (Dihydrouracil dehydrogenase) |
|  | *rstA* | P52108 | 1.08E-02 | 1.14 | Transcriptional regulatory protein RstA |
|  | *malF* | P02916 | 6.24E-09 | 1.14 | Maltose/maltodextrin transport system permease protein MalF |
|  | *hfq* | P0A6X3 | 2.65E-02 | 1.14 | RNA-binding protein Hfq (HF-1) (Host factor-I protein) (HF-I) |
|  | *ydhP* | P77389 | 1.30E-02 | 1.14 | Inner membrane transport protein YdhP |
|  | *envC* | P37690 | 5.50E-08 | 1.14 | Murein hydrolase activator EnvC (Septal ring factor) |
|  | *rfaH* | P0AFW0 | 6.11E-11 | 1.14 | Transcription antitermination protein RfaH |
|  | *yjjP* | P0ADD5 | 1.02E-11 | 1.14 | Inner membrane protein YjjP |
|  | *mazG* | P0AEY3 | 4.77E-03 | 1.13 | Nucleoside triphosphate pyrophosphohydrolase (NTP-PPase) (EC 3.6.1.8) |
|  | *yggP* | P52048 | 1.19E-36 | 1.13 | Uncharacterized protein YggP |
|  | *uraA* | P0AGM7 | 4.50E-03 | 1.13 | Uracil permease (Uracil transporter) |
|  | *metK* | P0A817 | 3.03E-05 | 1.13 | S-adenosylmethionine synthase (AdoMet synthase) (EC 2.5.1.6) (MAT) (Methionine adenosyltransferase) |
|  | *gudP* | Q46916 | 9.93E-04 | 1.13 | Probable glucarate transporter (D-glucarate permease) |
|  | *rlmJ* | P37634 | 6.78E-03 | 1.12 | Ribosomal RNA large subunit methyltransferase J (EC 2.1.1.266) (23S rRNA (adenine(2030)-N6)-methyltransferase) (23S rRNA m6A2030 methyltransferase) |
|  | *metE* | P25665 | 9.32E-05 | 1.12 | 5-methyltetrahydropteroyltriglutamate--homocysteine methyltransferase (EC 2.1.1.14) (Cobalamin-independent methionine synthase) (Methionine synthase, vitamin-B12 independent isozyme) |
|  | *ispH* | P62623 | 2.19E-06 | 1.12 | 4-hydroxy-3-methylbut-2-enyl diphosphate reductase (HMBPP reductase) (EC 1.17.7.4) (1-hydroxy-2-methyl-2-(E)-butenyl 4-diphosphate reductase) |
|  | *gntT* | P39835 | 9.35E-14 | 1.12 | High-affinity gluconate transporter (Gluconate permease) (Gnt-I system) |
|  | *hha* | P0ACE3 | 5.07E-08 | 1.12 | Hemolysin expression-modulating protein Hha |
|  | *fadL* | P10384 | 4.43E-04 | 1.12 | Long-chain fatty acid transport protein (Outer membrane FadL protein) (Outer membrane flp protein) |
|  | *malE* | P0AEX9 | 1.18E-04 | 1.11 | Maltose/maltodextrin-binding periplasmic protein (MMBP) (Maltodextrin-binding protein) (Maltose-binding protein) (MBP) |
|  | *ushA* | P07024 | 7.80E-06 | 1.11 | Protein UshA [Includes: UDP-sugar hydrolase (EC 3.6.1.45) (UDP-sugar diphosphatase) (UDP-sugar pyrophosphatase); 5'-nucleotidase (5'-NT) (EC 3.1.3.5)] |
|  | *secM* | P62395 | 9.65E-15 | 1.11 | Secretion monitor |
|  | *dxs* | P77488 | 4.24E-05 | 1.11 | 1-deoxy-D-xylulose-5-phosphate synthase (EC 2.2.1.7) (1-deoxyxylulose-5-phosphate synthase) (DXP synthase) (DXPS) |
|  | *trpA* | P0A877 | 1.05E-02 | 1.11 | Tryptophan synthase alpha chain (EC 4.2.1.20) |
|  | *ycaO* | P75838 | 3.32E-05 | 1.11 | Ribosomal protein S12 methylthiotransferase accessory factor YcaO |
|  | *csgE* | P0AE95 | 7.11E-06 | 1.10 | Curli production assembly/transport component CsgE |
|  | *ybaY* | P77717 | 1.22E-04 | 1.10 | Uncharacterized lipoprotein YbaY |
|  | *yebE* | P33218 | 7.46E-04 | 1.10 | Inner membrane protein YebE |
|  | *fhuE* | P16869 | 1.81E-10 | 1.10 | FhuE receptor (Outer-membrane receptor for Fe(III)-coprogen, Fe(III)-ferrioxamine B and Fe(III)-rhodotrulic acid) |
|  | *hypB* | P0AAN3 | 3.75E-04 | 1.10 | Hydrogenase maturation factor HypB (Hydrogenase isoenzymes nickel incorporation protein HypB) |
|  | *alaA* | P0A959 | 9.96E-07 | 1.09 | Glutamate-pyruvate aminotransferase AlaA (EC 2.6.1.2) |
|  | *ghoS* | P0AF61 | 9.02E-06 | 1.09 | Endoribonuclease antitoxin GhoS (EC 3.1.-.-) (Antitoxin GhoS) |
|  | *motB* | P0AF06 | 4.27E-03 | 1.09 | Motility protein B (Chemotaxis protein MotB) |
|  | *mdtN* | P32716 | 1.71E-04 | 1.09 | Multidrug resistance protein MdtN |
|  | *wecC* | P27829 | 1.97E-04 | 1.09 | UDP-N-acetyl-D-mannosamine dehydrogenase (EC 1.1.1.336) (UDP-ManNAc 6-dehydrogenase) |
|  | *rplA* | P0A7L0 | 2.64E-09 | 1.09 | 50S ribosomal protein L1 (Large ribosomal subunit protein uL1) |
|  | *trpE* | P00895 | 2.11E-14 | 1.09 | Anthranilate synthase component 1 (AS) (ASI) (EC 4.1.3.27) |
|  | *ubiD* | P0AAB4 | 6.22E-29 | 1.08 | 3-octaprenyl-4-hydroxybenzoate carboxy-lyase (EC 4.1.1.98) (Polyprenyl p-hydroxybenzoate decarboxylase) |
|  | *tsaC* | P45748 | 6.33E-11 | 1.08 | Threonylcarbamoyl-AMP synthase (TC-AMP synthase) (EC 2.7.7.87) (L-threonylcarbamoyladenylate synthase) (Ribosome maturation factor TsaC) (t(6)A37 threonylcarbamoyladenosine biosynthesis protein TsaC) (tRNA threonylcarbamoyladenosine biosynthesis protein TsaC) |
|  | *ydgJ* | P77376 | 1.61E-05 | 1.08 | Uncharacterized oxidoreductase YdgJ (EC 1.-.-.-) |
|  | *pyrC* | P05020 | 6.22E-06 | 1.08 | Dihydroorotase (DHOase) (EC 3.5.2.3) |
|  | *frmR* | P0AAP3 | 5.70E-03 | 1.08 | Transcriptional repressor FrmR |
|  | *maa* | P77791 | 1.75E-05 | 1.08 | Maltose O-acetyltransferase (MAT) (EC 2.3.1.79) (Maltose transacetylase) |
|  | *accC* | P24182 | 5.68E-03 | 1.07 | Biotin carboxylase (EC 6.3.4.14) (Acetyl-CoA carboxylase subunit A) (ACC) (EC 6.4.1.2) |
|  | *lolA* | P61316 | 4.01E-06 | 1.07 | Outer-membrane lipoprotein carrier protein (P20) |
|  | *thiI* | P77718 | 1.17E-05 | 1.07 | tRNA sulfurtransferase (EC 2.8.1.4) (Sulfur carrier protein ThiS sulfurtransferase) (Thiamine biosynthesis protein ThiI) (tRNA 4-thiouridine synthase) |
|  | *glpG* | P09391 | 2.25E-03 | 1.07 | Rhomboid protease GlpG (EC 3.4.21.105) (Intramembrane serine protease) |
|  | *cheW* | P0A964 | 2.14E-02 | 1.07 | Chemotaxis protein CheW |
|  | *rapA* | P60240 | 6.46E-03 | 1.07 | RNA polymerase-associated protein RapA (EC 3.6.4.-) (ATP-dependent helicase HepA) |
|  | *rbbA* | P37624 | 1.83E-05 | 1.06 | Ribosome-associated ATPase (Ribosomal bound ATPase) |
|  | *chiQ* | P75734 | 2.69E-05 | 1.06 | Uncharacterized lipoprotein ChiQ |
|  | *rihC* | P22564 | 8.56E-13 | 1.06 | Non-specific ribonucleoside hydrolase RihC (EC 3.2.-.-) (Purine/pyrimidine ribonucleoside hydrolase) |
|  | *thiL* | P0AGG0 | 5.40E-12 | 1.05 | Thiamine-monophosphate kinase (TMP kinase) (Thiamine-phosphate kinase) (EC 2.7.4.16) |
|  | *ppiA* | P0AFL3 | 4.30E-04 | 1.05 | Peptidyl-prolyl cis-trans isomerase A (PPIase A) (EC 5.2.1.8) (Cyclophilin A) (Rotamase A) |
|  | *endA* | P25736 | 3.22E-09 | 1.05 | Endonuclease-1 (EC 3.1.21.1) (Endonuclease I) (Endo I) |
|  | *frwD* | P32676 | 2.74E-08 | 1.05 | PTS system fructose-like EIIB component 3 (EC 2.7.1.202) (Fructose-like phosphotransferase enzyme IIB component 3) |
|  | *yehR* | P33354 | 1.61E-03 | 1.05 | Uncharacterized lipoprotein YehR |
|  | *fkpB* | P0AEM0 | 7.26E-07 | 1.05 | FKBP-type 16 kDa peptidyl-prolyl cis-trans isomerase (PPIase) (EC 5.2.1.8) (Rotamase) |
|  | *bioH* | P13001 | 1.37E-03 | 1.04 | Pimeloyl-[acyl-carrier protein] methyl ester esterase (EC 3.1.1.85) (Biotin synthesis protein BioH) (Carboxylesterase BioH) |
|  | *dapB* | P04036 | 3.52E-05 | 1.04 | 4-hydroxy-tetrahydrodipicolinate reductase (HTPA reductase) (EC 1.17.1.8) |
|  | *citG* | P77231 | 1.63E-04 | 1.04 | 2-(5''-triphosphoribosyl)-3'-dephosphocoenzyme-A synthase (2-(5''-triphosphoribosyl)-3'-dephospho-CoA synthase) (EC 2.4.2.52) |
|  | *cyoC* | P0ABJ3 | 1.24E-03 | 1.03 | Cytochrome bo(3) ubiquinol oxidase subunit 3 (Cytochrome o ubiquinol oxidase subunit 3) (Cytochrome o subunit 3) (Oxidase bo(3) subunit 3) (Ubiquinol oxidase chain C) (Ubiquinol oxidase polypeptide III) (Ubiquinol oxidase subunit 3) |
|  | *napH* | P33934 | 4.37E-02 | 1.03 | Ferredoxin-type protein NapH (Ubiquinol--[NapC cytochrome c] reductase NapH subunit) |
|  | *betI* | P17446 | 6.44E-05 | 1.03 | HTH-type transcriptional regulator BetI |
|  | *dcp* | P24171 | 6.41E-07 | 1.02 | Dipeptidyl carboxypeptidase (EC 3.4.15.5) (Peptidyl-dipeptidase Dcp) |
|  | *deoA* | P07650 | 2.53E-03 | 1.02 | Thymidine phosphorylase (EC 2.4.2.4) (TdRPase) |
|  | *ydgK* | P76180 | 1.12E-04 | 1.02 | Inner membrane protein YdgK |
|  | *cvpA* | P08550 | 3.31E-05 | 1.02 | Colicin V production protein (Protein DedE) (Pur regulon 18 kDa protein) |
|  | *phnL* | P16679 | 4.45E-03 | 1.02 | Alpha-D-ribose 1-methylphosphonate 5-triphosphate synthase subunit PhnL (RPnTP synthase subunit PhnL) (EC 2.7.8.37) |
|  | *ilvA* | P04968 | 1.42E-06 | 1.02 | L-threonine dehydratase biosynthetic IlvA (EC 4.3.1.19) (Threonine deaminase) |
|  | *queG* | P39288 | 1.59E-10 | 1.02 | Epoxyqueuosine reductase (EC 1.17.99.6) (Queuosine biosynthesis protein QueG) |
|  | *mltG* | P28306 | 2.04E-06 | 1.01 | Endolytic murein transglycosylase (EC 4.2.2.-) (Peptidoglycan polymerization terminase) |
|  | *rbfA* | P0A7G2 | 4.26E-08 | 1.01 | 30S ribosome-binding factor (Protein P15B) (Ribosome-binding factor A) (RbfA) |
|  | *cmtB* | P69824 | 3.56E-08 | 1.01 | Mannitol-specific cryptic phosphotransferase enzyme IIA component (EIIA-Mtl) (EIII-Mtl) (PTS system mannitol-specific EIIA component) |
|  | *gspL* | P45763 | 6.62E-10 | 1.01 | Putative type II secretion system protein L (T2SS protein L) (Putative general secretion pathway protein L) |
|  | *yehA* | P33340 | 1.48E-06 | 1.00 | Uncharacterized fimbrial-like protein YehA |
|  | *yebF* | P33219 | 1.62E-03 | 1.00 | Protein YebF |
|  | *rluD* | P33643 | 3.43E-05 | 1.00 | Ribosomal large subunit pseudouridine synthase D (EC 5.4.99.23) (23S rRNA pseudouridine(1911/1915/1917) synthase) (rRNA pseudouridylate synthase D) (rRNA-uridine isomerase D) |
|  | *secB* | P0AG86 | 4.39E-05 | 1.00 | Protein-export protein SecB (Chaperone SecB) |
|  | *ada* | P06134 | 5.36E-14 | 1.00 | Bifunctional transcriptional activator/DNA repair enzyme Ada (Regulatory protein of adaptive response) [Includes: Methylphosphotriester-DNA--protein-cysteine S-methyltransferase (EC 2.1.1.n11) (Methylphosphotriester-DNA methyltransferase); Methylated-DNA--protein-cysteine methyltransferase (EC 2.1.1.63) (O6-methylguanine-DNA alkyltransferase)] |
| **Gene Groups** | **Gene Name** | **UniProt ID** | **P-value** | **Log2(Fold_Change)** | **Functions** |
| Down-regulated Genes | *glrK* | P52101 | 6.0E-134 | -4.50 | Sensor histidine kinase GlrK (EC 2.7.13.3) |
|  | *tolB* | P0A855 | 3.4E-33 | -4.29 | Tol-Pal system protein TolB |
|  | *pmbA* | P0AFK0 | 8.9E-194 | -4.29 | Metalloprotease PmbA (EC 3.4.-.-) (Protein TldE) |
|  | *nikA* | P33590 | 1.5E-68 | -3.90 | Nickel-binding periplasmic protein |
|  | *mhpE* | P51020 | 4.5E-27 | -3.79 | 4-hydroxy-2-oxovalerate aldolase (HOA) (EC 4.1.3.39) (4-hydroxy-2-keto-pentanoic acid aldolase) (4-hydroxy-2-oxopentanoate aldolase) |
|  | *yniD* | Q2EES1 | 5.1E-43 | -3.77 | Uncharacterized protein YniD |
|  | *yliI* | P75804 | 1.3E-63 | -3.61 | Aldose sugar dehydrogenase YliI (Asd) (EC 1.1.5.-) (Soluble aldose sugar dehydrogenase YliI) |
|  | *ybeZ* | P0A9K3 | 1.4E-17 | -3.42 | PhoH-like protein |
|  | *kdpB* | P03960 | 3.5E-31 | -3.40 | Potassium-transporting ATPase ATP-binding subunit (EC 7.2.2.6) (ATP phosphohydrolase [potassium-transporting] B chain) (Potassium-binding and translocating subunit B) (Potassium-translocating ATPase B chain) |
|  | *sapC* | P0AGH5 | 4.7E-17 | -3.39 | Putrescine export system permease protein SapC |
|  | *ubiC* | P26602 | 1.6E-17 | -3.36 | Chorismate pyruvate-lyase (CL) (CPL) (EC 4.1.3.40) |
|  | *slyA* | P0A8W2 | 1.2E-58 | -3.34 | Transcriptional regulator SlyA |
|  | *tauA* | Q47537 | 7.6E-06 | -3.34 | Taurine-binding periplasmic protein (Sulfate starvation-induced protein 1) (SSI1) |
|  | *nrdG* | P0A9N8 | 1.1E-48 | -3.33 | Anaerobic ribonucleoside-triphosphate reductase-activating protein (EC 1.97.1.-) (Class III anaerobic ribonucleotide reductase small component) |
|  | *thiE* | P30137 | 2.6E-56 | -3.28 | Thiamine-phosphate synthase (TP synthase) (TPS) (EC 2.5.1.3) (Thiamine-phosphate pyrophosphorylase) (TMP pyrophosphorylase) (TMP-PPase) |
|  | *tolA* | P19934 | 9.3E-15 | -3.28 | Tol-Pal system protein TolA |
|  | *modB* | P0AF01 | 2.3E-23 | -3.28 | Molybdenum transport system permease protein ModB |
|  | *ydfR* | P76160 | 1.4E-37 | -3.27 | Uncharacterized protein YdfR |
|  | *ybeY* | P0A898 | 3.3E-15 | -3.21 | Endoribonuclease YbeY (EC 3.1.-.-) |
|  | *putA* | P09546 | 1.3E-26 | -3.19 | Bifunctional protein PutA [Includes: Proline dehydrogenase (EC 1.5.5.2) (Proline oxidase); Delta-1-pyrroline-5-carboxylate dehydrogenase (P5C dehydrogenase) (EC 1.2.1.88) (L-glutamate gamma-semialdehyde dehydrogenase)] |
|  | *rnc* | P0A7Y0 | 7.7E-24 | -3.17 | Ribonuclease 3 (EC 3.1.26.3) (Ribonuclease III) (RNase III) |
|  | *kdpA* | P03959 | 9.3E-33 | -3.15 | Potassium-transporting ATPase potassium-binding subunit (ATP phosphohydrolase [potassium-transporting] A chain) (Potassium-binding and translocating subunit A) (Potassium-translocating ATPase A chain) |
|  | *rplU* | P0AG48 | 6.3E-101 | -3.14 | 50S ribosomal protein L21 (Large ribosomal subunit protein bL21) |
|  | *rlmB* | P63177 | 1.9E-08 | -3.11 | 23S rRNA (guanosine-2'-O-)-methyltransferase RlmB (EC 2.1.1.185) (23S rRNA (guanosine2251 2'-O)-methyltransferase) (23S rRNA Gm2251 2'-O-methyltransferase) |
|  | *ppiD* | P0ADY1 | 4.3E-18 | -3.08 | Periplasmic chaperone PpiD (Periplasmic folding chaperone) |
|  | *pdxY* | P77150 | 1.7E-24 | -3.02 | Pyridoxal kinase PdxY (PL kinase) (EC 2.7.1.35) (Pyridoxal kinase 2) (PL kinase 2) |
|  | *pntA* | P07001 | 7.8E-25 | -2.97 | NAD(P) transhydrogenase subunit alpha (EC 7.1.1.1) (Nicotinamide nucleotide transhydrogenase subunit alpha) (Pyridine nucleotide transhydrogenase subunit alpha) |
|  | *yddG* | P46136 | 5.9E-47 | -2.94 | Aromatic amino acid exporter YddG |
|  | *folA* | P0ABQ4 | 1.0E-38 | -2.94 | Dihydrofolate reductase (EC 1.5.1.3) |
|  | *dapF* | P0A6K1 | 5.3E-39 | -2.93 | Diaminopimelate epimerase (DAP epimerase) (EC 5.1.1.7) (PLP-independent amino acid racemase) |
|  | *thiF* | P30138 | 3.9E-74 | -2.92 | Sulfur carrier protein ThiS adenylyltransferase (EC 2.7.7.73) |
|  | *puuP* | P76037 | 1.2E-08 | -2.83 | Putrescine importer PuuP |
|  | *yhbE* | P0AA73 | 2.1E-46 | -2.82 | Uncharacterized inner membrane transporter YhbE |
|  | *rpmA* | P0A7L8 | 1.1E-59 | -2.82 | 50S ribosomal protein L27 (Large ribosomal subunit protein bL27) |
|  | *tyrS* | P0AGJ9 | 7.9E-28 | -2.82 | Tyrosine--tRNA ligase (EC 6.1.1.1) (Tyrosyl-tRNA synthetase) (TyrRS) |
|  | *sapA* | Q47622 | 9.6E-12 | -2.81 | Probable ABC transporter periplasmic-binding protein SapA |
|  | *ypdB* | P0AE39 | 3.6E-41 | -2.79 | Transcriptional regulatory protein YpdB |
|  | *qseG* | P0AD44 | 5.0E-45 | -2.78 | Uncharacterized protein YfhG |
|  | *sapD* | P0AAH4 | 5.3E-11 | -2.76 | Putrescine export system ATP-binding protein SapD |
|  | *mhpF* | P77580 | 1.1E-12 | -2.76 | Acetaldehyde dehydrogenase (EC 1.2.1.10) (Acetaldehyde dehydrogenase [acetylating]) |
|  | *potB* | P0AFK4 | 6.7E-54 | -2.74 | Spermidine/putrescine transport system permease protein PotB |
|  | *lepB* | P00803 | 1.4E-18 | -2.72 | Signal peptidase I (SPase I) (EC 3.4.21.89) (Leader peptidase I) |
|  | *uhpC* | P09836 | 1.9E-20 | -2.63 | Membrane sensor protein UhpC |
|  | *rpoZ* | P0A800 | 5.0E-17 | -2.60 | DNA-directed RNA polymerase subunit omega (RNAP omega subunit) (EC 2.7.7.6) (RNA polymerase omega subunit) (Transcriptase subunit omega) |
|  | *aroC* | P12008 | 1.1E-24 | -2.59 | Chorismate synthase (CS) (EC 4.2.3.5) (5-enolpyruvylshikimate-3-phosphate phospholyase) (EPSP phospholyase) |
|  | *puuB* | P37906 | 2.3E-07 | -2.58 | Gamma-glutamylputrescine oxidoreductase (Gamma-Glu-Put oxidase) (Gamma-glutamylputrescine oxidase) (EC 1.4.3.-) |
|  | *tauB* | Q47538 | 1.4E-17 | -2.58 | Taurine import ATP-binding protein TauB (EC 7.6.2.7) |
|  | *rplV* | P61175 | 7.6E-18 | -2.55 | 50S ribosomal protein L22 (Large ribosomal subunit protein uL22) |
|  | *infC* | P0A707 | 4.5E-16 | -2.55 | Translation initiation factor IF-3 [Cleaved into: Translation initiation factor IF-3, N-terminally processed; Translation initiation factor IF-3S] |
|  | *phoE* | P02932 | 3.8E-18 | -2.55 | Outer membrane porin PhoE (Outer membrane pore protein E) |
|  | *hemB* | P0ACB2 | 2.3E-32 | -2.54 | Delta-aminolevulinic acid dehydratase (ALAD) (ALADH) (EC 4.2.1.24) (Porphobilinogen synthase) |
|  | *pfkB* | P06999 | 2.3E-35 | -2.51 | ATP-dependent 6-phosphofructokinase isozyme 2 (ATP-PFK 2) (Phosphofructokinase 2) (EC 2.7.1.11) (6-phosphofructokinase isozyme II) (Phosphohexokinase 2) |
|  | *proV* | P14175 | 4.4E-49 | -2.50 | Glycine betaine/proline betaine transport system ATP-binding protein ProV |
|  | *ddpX* | P77790 | 4.9E-39 | -2.46 | D-alanyl-D-alanine dipeptidase (D-Ala-D-Ala dipeptidase) (EC 3.4.13.22) |
|  | *atoA* | P76459 | 2.9E-27 | -2.44 | Acetate CoA-transferase subunit beta (EC 2.8.3.8) (Acetyl-CoA:acetoacetate CoA-transferase subunit beta) |
|  | *dsbC* | P0AEG6 | 3.7E-31 | -2.40 | Thiol:disulfide interchange protein DsbC |
|  | *gstA* | P0A9D2 | 2.4E-28 | -2.38 | Glutathione S-transferase GstA (EC 2.5.1.18) (GST B1-1) |
|  | *nikB* | P33591 | 1.3E-21 | -2.33 | Nickel transport system permease protein NikB |
|  | *yejK* | P33920 | 2.6E-10 | -2.32 | Nucleoid-associated protein YejK |
|  | *argH* | P11447 | 1.4E-06 | -2.30 | Argininosuccinate lyase (ASAL) (EC 4.3.2.1) (Arginosuccinase) |
|  | *proB* | P0A7B5 | 2.3E-24 | -2.29 | Glutamate 5-kinase (EC 2.7.2.11) (Gamma-glutamyl kinase) (GK) |
|  | *atpC* | P0A6E6 | 7.9E-11 | -2.28 | ATP synthase epsilon chain (ATP synthase F1 sector epsilon subunit) (F-ATPase epsilon subunit) |
|  | *cbtA* | P64524 | 3.8E-24 | -2.27 | Cytoskeleton-binding toxin CbtA (Toxin CbtA) (Toxin YeeV) |
|  | *rppH* | P0A776 | 2.1E-10 | -2.26 | RNA pyrophosphohydrolase (EC 3.6.1.-) ((Di)nucleoside polyphosphate hydrolase) (Ap5A pyrophosphatase) |
|  | *atpH* | P0ABA4 | 1.1E-29 | -2.25 | ATP synthase subunit delta (ATP synthase F(1) sector subunit delta) (F-type ATPase subunit delta) (F-ATPase subunit delta) |
|  | *plsY* | P60782 | 5.8E-12 | -2.23 | Probable glycerol-3-phosphate acyltransferase (G3P acyltransferase) (GPAT) (EC 2.3.1.15) (EC 2.3.1.n5) (Lysophosphatidic acid synthase) (LPA synthase) |
|  | *pssA* | P23830 | 1.4E-07 | -2.22 | CDP-diacylglycerol--serine O-phosphatidyltransferase (EC 2.7.8.8) (Phosphatidylserine synthase) |
|  | *pdxH* | P0AFI7 | 1.5E-30 | -2.20 | Pyridoxine/pyridoxamine 5'-phosphate oxidase (EC 1.4.3.5) (PNP/PMP oxidase) (PNPOx) (Pyridoxal 5'-phosphate synthase) |
|  | *yjiA* | P24203 | 3.9E-34 | -2.19 | P-loop guanosine triphosphatase YjiA (EC 3.6.-.-) (GTP-binding protein YjiA) |
|  | *purL* | P15254 | 7.3E-24 | -2.13 | Phosphoribosylformylglycinamidine synthase (FGAM synthase) (FGAMS) (EC 6.3.5.3) (Formylglycinamide ribonucleotide amidotransferase) (FGAR amidotransferase) (FGAR-AT) |
|  | *rbsB* | P02925 | 2.4E-05 | -2.12 | Ribose import binding protein RbsB |
|  | *pxpA* | P75746 | 4.7E-32 | -2.11 | 5-oxoprolinase subunit A (5-OPase subunit A) (EC 3.5.2.9) (5-oxoprolinase (ATP-hydrolyzing) subunit A) |
|  | *fliE* | P0A8T5 | 1.2E-23 | -2.11 | Flagellar hook-basal body complex protein FliE |
|  | *atoC* | Q06065 | 1.8E-33 | -2.11 | Regulatory protein AtoC (Acetoacetate metabolism regulatory protein) (DNA-binding transcriptional regulator AtoC) (Ornithine decarboxylase antizyme) |
|  | *puuA* | P78061 | 7.5E-08 | -2.10 | Gamma-glutamylputrescine synthetase PuuA (Gamma-Glu-Put synthetase) (EC 6.3.1.11) (Glutamate--putrescine ligase) |
|  | *rpsO* | P0ADZ4 | 2.9E-07 | -2.10 | 30S ribosomal protein S15 (Small ribosomal subunit protein uS15) |
|  | *purT* | P33221 | 7.5E-21 | -2.10 | Formate-dependent phosphoribosylglycinamide formyltransferase (5'-phosphoribosylglycinamide transformylase 2) (Formate-dependent GAR transformylase) (EC 2.1.2.-) (GAR transformylase 2) (GART 2) (Non-folate glycinamide ribonucleotide transformylase) (Phosphoribosylglycinamide formyltransferase 2) |
|  | *ulaF* | P39306 | 3.8E-19 | -2.10 | L-ribulose-5-phosphate 4-epimerase UlaF (EC 5.1.3.4) (L-ascorbate utilization protein F) (Phosphoribulose isomerase) |
|  | *ypdA* | P0AA93 | 1.8E-17 | -2.10 | Sensor histidine kinase YpdA (EC 2.7.13.3) |
|  | *atoS* | Q06067 | 1.2E-42 | -2.09 | Signal transduction histidine-protein kinase AtoS (EC 2.7.13.3) |
|  | *nei* | P50465 | 4.9E-10 | -2.08 | Endonuclease 8 (DNA glycosylase/AP lyase Nei) (EC 3.2.2.-) (EC 4.2.99.18) (DNA-(apurinic or apyrimidinic site) lyase Nei) (Endonuclease VIII) |
|  | *yidK* | P31448 | 5.5E-19 | -2.07 | Uncharacterized symporter YidK |
|  | *cynS* | P00816 | 2.6E-10 | -2.06 | Cyanate hydratase (Cyanase) (EC 4.2.1.104) (Cyanate hydrolase) (Cyanate lyase) |
|  | *yfjS* | O52982 | 1.6E-14 | -2.06 | Lipoprotein YfjS |
|  | *rpsC* | P0A7V3 | 8.7E-14 | -2.06 | 30S ribosomal protein S3 (Small ribosomal subunit protein uS3) |
|  | *yphB* | P76584 | 1.6E-15 | -2.05 | Uncharacterized protein YphB |
|  | *hybC* | P0ACE0 | 5.5E-27 | -2.03 | Hydrogenase-2 large chain (HYD2) (EC 1.12.99.6) (Membrane-bound hydrogenase 2 large subunit) (NiFe hydrogenase) |
|  | *yhdE* | P25536 | 5.0E-13 | -2.02 | dTTP/UTP pyrophosphatase (dTTPase/UTPase) (EC 3.6.1.9) (Nucleoside triphosphate pyrophosphatase) (Nucleotide pyrophosphatase) (Nucleotide PPase) |
|  | *yiiM* | P32157 | 3.4E-10 | -2.02 | Protein YiiM |
|  | *fhuB* | P06972 | 2.4E-19 | -2.02 | Iron(3+)-hydroxamate import system permease protein FhuB (Ferric hydroxamate uptake protein B) (Ferrichrome transport system permease protein FhuB) (Ferrichrome uptake protein FhuB) (Iron(III)-hydroxamate import system permease protein FhuB) |
|  | *proW* | P14176 | 1.5E-21 | -2.02 | Glycine betaine/proline betaine transport system permease protein ProW |
|  | *nagZ* | P75949 | 1.9E-18 | -2.01 | Beta-hexosaminidase (EC 3.2.1.52) (Beta-N-acetylhexosaminidase) (N-acetyl-beta-glucosaminidase) |
|  | *lgoD* | P39400 | 2.3E-11 | -1.97 | L-galactonate-5-dehydrogenase (EC 1.1.1.414) |
|  | *rpmI* | P0A7Q1 | 1.9E-11 | -1.97 | 50S ribosomal protein L35 (Large ribosomal subunit protein bL35) (Ribosomal protein A) |
|  | *cdd* | P0ABF6 | 2.5E-21 | -1.95 | Cytidine deaminase (EC 3.5.4.5) (Cytidine aminohydrolase) (CDA) |
|  | *yccJ* | P0AB14 | 4.3E-08 | -1.95 | Uncharacterized protein YccJ |
|  | *folE* | P0A6T5 | 1.4E-29 | -1.95 | GTP cyclohydrolase 1 (EC 3.5.4.16) (GTP cyclohydrolase I) (GTP-CH-I) |
|  | *hchA* | P31658 | 1.1E-11 | -1.94 | Protein/nucleic acid deglycase 1 (EC 3.1.2.-) (EC 3.5.1.-) (EC 3.5.1.124) (Glyoxalase III) (EC 4.2.1.130) (Holding molecular chaperone) (Hsp31) (Maillard deglycase) |
|  | *pdxI* | P25906 | 6.8E-22 | -1.94 | Pyridoxine 4-dehydrogenase (EC 1.1.1.65) |
|  | *truD* | Q57261 | 3.8E-27 | -1.94 | tRNA pseudouridine synthase D (EC 5.4.99.27) (tRNA pseudouridine(13) synthase) (tRNA pseudouridylate synthase D) (tRNA-uridine isomerase D) |
|  | *aspC* | P00509 | 2.9E-36 | -1.94 | Aspartate aminotransferase (AspAT) (EC 2.6.1.1) (Transaminase A) |
|  | *btuE* | P06610 | 2.2E-41 | -1.93 | Thioredoxin/glutathione peroxidase BtuE (EC 1.11.1.24) (EC 1.11.1.9) |
|  | *hisP* | P07109 | 4.5E-10 | -1.92 | Histidine transport ATP-binding protein HisP |
|  | *gcvA* | P0A9F6 | 2.3E-21 | -1.92 | Glycine cleavage system transcriptional activator (Gcv operon activator) |
|  | *yeeR* | P76361 | 1.0E-20 | -1.92 | Inner membrane protein YeeR |
|  | *proA* | P07004 | 4.7E-20 | -1.91 | Gamma-glutamyl phosphate reductase (GPR) (EC 1.2.1.41) (Glutamate-5-semialdehyde dehydrogenase) (Glutamyl-gamma-semialdehyde dehydrogenase) (GSA dehydrogenase) |
|  | *dnaB* | P0ACB0 | 3.0E-13 | -1.90 | Replicative DNA helicase (EC 3.6.4.12) |
|  | *puuE* | P50457 | 2.3E-05 | -1.90 | 4-aminobutyrate aminotransferase PuuE (EC 2.6.1.19) (GABA aminotransferase) (GABA-AT) (Gamma-amino-N-butyrate transaminase) (GABA transaminase) (Glutamate:succinic semialdehyde transaminase) |
|  | *ppc* | P00864 | 2.9E-10 | -1.89 | Phosphoenolpyruvate carboxylase (PEPC) (PEPCase) (EC 4.1.1.31) |
|  | *cysC* | P0A6J1 | 3.3E-30 | -1.89 | Adenylyl-sulfate kinase (EC 2.7.1.25) (APS kinase) (ATP adenosine-5'-phosphosulfate 3'-phosphotransferase) (Adenosine-5'-phosphosulfate kinase) |
|  | *tsaD* | P05852 | 3.7E-10 | -1.89 | tRNA N6-adenosine threonylcarbamoyltransferase (EC 2.3.1.234) (N6-L-threonylcarbamoyladenine synthase) (t(6)A synthase) (t(6)A37 threonylcarbamoyladenosine biosynthesis protein TsaD) (tRNA threonylcarbamoyladenosine biosynthesis protein TsaD) |
|  | *uvrY* | P0AED5 | 6.8E-17 | -1.88 | Response regulator UvrY |
|  | *caiB* | P31572 | 1.1E-06 | -1.86 | L-carnitine CoA-transferase (EC 2.8.3.21) (Crotonobetainyl-CoA:carnitine CoA-transferase) |
|  | *ybhK* | P75767 | 1.1E-06 | -1.86 | Putative gluconeogenesis factor |
|  | *pheA* | P0A9J8 | 1.8E-50 | -1.86 | Bifunctional chorismate mutase/prephenate dehydratase (Chorismate mutase-prephenate dehydratase) (P-protein) [Includes: Chorismate mutase (CM) (EC 5.4.99.5); Prephenate dehydratase (PDT) (EC 4.2.1.51)] |
|  | *ybhC* | P46130 | 5.4E-29 | -1.85 | Putative acyl-CoA thioester hydrolase YbhC (EC 3.1.2.-) |
|  | *ykfI* | P77692 | 2.1E-12 | -1.85 | Toxin YkfI |
|  | *yfcP* | P76499 | 1.9E-14 | -1.84 | Uncharacterized fimbrial-like protein YfcP |
|  | *hyfE* | P0AEW1 | 7.8E-18 | -1.84 | Hydrogenase-4 component E (EC 1.-.-.-) |
|  | *mrr* | P24202 | 2.8E-24 | -1.84 | Mrr restriction system protein (EcoKMrr) |
|  | *ydcO* | P76103 | 4.1E-21 | -1.83 | Inner membrane protein YdcO |
|  | *garK* | P23524 | 3.7E-20 | -1.83 | Glycerate 2-kinase (EC 2.7.1.165) (Glycerate kinase 1) (GK1) |
|  | *rpsF* | P02358 | 1.5E-07 | -1.82 | 30S ribosomal protein S6 (Small ribosomal subunit protein bS6) [Cleaved into: 30S ribosomal protein S6, fully modified isoform; 30S ribosomal protein S6, non-modified isoform] |
|  | *oppB* | P0AFH2 | 1.7E-18 | -1.81 | Oligopeptide transport system permease protein OppB |
|  | *narU* | P37758 | 1.8E-28 | -1.80 | Nitrate/nitrite transporter NarU (Nitrite extrusion protein 2) (Nitrite facilitator 2) |
|  | *proP* | P0C0L7 | 1.3E-28 | -1.78 | Proline/betaine transporter (Proline porter II) (PPII) |
|  | *mutT* | P08337 | 6.7E-25 | -1.78 | 8-oxo-dGTP diphosphatase (8-oxo-dGTPase) (EC 3.6.1.55) (7,8-dihydro-8-oxoguanine-triphosphatase) (Mutator protein MutT) (dGTP pyrophosphohydrolase) |
|  | *cynT* | P0ABE9 | 3.7E-14 | -1.77 | Carbonic anhydrase 1 (EC 4.2.1.1) (Carbonate dehydratase 1) |
|  | *folP* | P0AC13 | 3.7E-21 | -1.76 | Dihydropteroate synthase (DHPS) (EC 2.5.1.15) (Dihydropteroate pyrophosphorylase) |
|  | *obgE* | P42641 | 2.1E-26 | -1.74 | GTPase ObgE/CgtA (EC 3.6.5.-) (GTP-binding protein Obg) |
|  | *araD* | P08203 | 5.2E-13 | -1.74 | L-ribulose-5-phosphate 4-epimerase AraD (EC 5.1.3.4) (Phosphoribulose isomerase) |
|  | *pxpC* | P75745 | 2.1E-08 | -1.73 | 5-oxoprolinase subunit C (5-OPase subunit C) (EC 3.5.2.9) (5-oxoprolinase (ATP-hydrolyzing) subunit C) |
|  | *nirD* | P0A9I8 | 1.6E-18 | -1.73 | Nitrite reductase (NADH) small subunit (EC 1.7.1.15) |
|  | *tyrA* | P07023 | 2.9E-47 | -1.73 | T-protein [Includes: Chorismate mutase (CM) (EC 5.4.99.5); Prephenate dehydrogenase (PDH) (EC 1.3.1.12)] |
|  | *srlB* | P05706 | 5.2E-11 | -1.72 | PTS system glucitol/sorbitol-specific EIIA component (EIIA-Gut) (EIII-Gut) (Glucitol/sorbitol-specific phosphotransferase enzyme IIA component) |
|  | *atoD* | P76458 | 2.4E-19 | -1.72 | Acetate CoA-transferase subunit alpha (EC 2.8.3.8) (Acetyl-CoA:acetoacetate-CoA transferase subunit alpha) |
|  | *hslJ* | P52644 | 4.2E-11 | -1.72 | Heat shock protein HslJ |
|  | *acrE* | P24180 | 7.4E-07 | -1.71 | Multidrug export protein AcrE (Acriflavine resistance protein E) (Protein EnvC) |
|  | *cusS* | P77485 | 5.0E-12 | -1.70 | Sensor histidine kinase CusS (EC 2.7.13.3) |
|  | *pgm* | P36938 | 1.1E-14 | -1.69 | Phosphoglucomutase (PGM) (EC 5.4.2.2) (Glucose phosphomutase) |
|  | *cmk* | P0A6I0 | 1.0E-18 | -1.69 | Cytidylate kinase (CK) (EC 2.7.4.25) (Cytidine monophosphate kinase) (CMP kinase) (Protein MssA) (p25) |
|  | *crfC* | P0DM85 | 2.4E-09 | -1.68 | Clamp-binding protein CrfC (Clamp-binding sister replication fork colocalization protein) |
|  | *gatA* | P69828 | 1.6E-12 | -1.68 | PTS system galactitol-specific EIIA component (EIIB-Gat) (Galactitol-specific phosphotransferase enzyme IIA component) |
|  | *pgl* | P52697 | 7.3E-14 | -1.68 | 6-phosphogluconolactonase (6-P-gluconolactonase) (Pgl) (EC 3.1.1.31) |
|  | *metG* | P00959 | 2.0E-29 | -1.68 | Methionine--tRNA ligase (EC 6.1.1.10) (Methionyl-tRNA synthetase) (MetRS) |
|  | *aer* | P50466 | 1.5E-13 | -1.68 | Aerotaxis receptor |
|  | *rnk* | P0AFW4 | 4.1E-12 | -1.67 | Regulator of nucleoside diphosphate kinase |
|  | *ybfF* | P75736 | 3.0E-04 | -1.67 | Esterase YbfF (EC 3.1.-.-) |
|  | *ddpA* | P76128 | 6.6E-18 | -1.65 | Probable D,D-dipeptide-binding periplasmic protein DdpA |
|  | *rplW* | P0ADZ0 | 2.2E-09 | -1.65 | 50S ribosomal protein L23 (Large ribosomal subunit protein uL23) |
|  | *holE* | P0ABS8 | 3.1E-05 | -1.65 | DNA polymerase III subunit theta (EC 2.7.7.7) |
|  | *tauD* | P37610 | 9.7E-13 | -1.64 | Alpha-ketoglutarate-dependent taurine dioxygenase (EC 1.14.11.17) (2-aminoethanesulfonate dioxygenase) (Sulfate starvation-induced protein 3) (SSI3) |
|  | *recO* | P0A7H3 | 2.9E-14 | -1.64 | DNA repair protein RecO (Recombination protein O) |
|  | *gspC* | P45757 | 1.2E-21 | -1.63 | Putative type II secretion system protein C (T2SS protein C) (Putative general secretion pathway protein C) |
|  | *metN* | P30750 | 9.2E-04 | -1.62 | Methionine import ATP-binding protein MetN (EC 7.4.2.11) |
|  | *lysA* | P00861 | 4.6E-08 | -1.62 | Diaminopimelate decarboxylase (DAP decarboxylase) (DAPDC) (EC 4.1.1.20) |
|  | *agp* | P19926 | 2.9E-08 | -1.62 | Glucose-1-phosphatase (G1Pase) (EC 3.1.3.10) |
|  | *rhtA* | P0AA67 | 3.5E-09 | -1.61 | Threonine/homoserine exporter RhtA |
|  | *mhpD* | P77608 | 1.1E-06 | -1.61 | 2-keto-4-pentenoate hydratase (EC 4.2.1.80) (2-hydroxypentadienoic acid hydratase) |
|  | *ispF* | P62617 | 1.3E-16 | -1.61 | 2-C-methyl-D-erythritol 2,4-cyclodiphosphate synthase (MECDP-synthase) (MECPP-synthase) (MECPS) (EC 4.6.1.12) |
|  | *ygbE* | P46141 | 2.9E-15 | -1.60 | Inner membrane protein YgbE |
|  | *yfcO* | P76498 | 6.4E-14 | -1.60 | Uncharacterized protein YfcO |
|  | *gcl* | P0AEP7 | 7.5E-12 | -1.58 | Glyoxylate carboligase (EC 4.1.1.47) (Tartronate-semialdehyde synthase) |
|  | *zapD* | P36680 | 1.1E-23 | -1.58 | Cell division protein ZapD (Z ring-associated protein D) |
|  | *dnaG* | P0ABS5 | 2.0E-08 | -1.58 | DNA primase (EC 2.7.7.101) |
|  | *sad* | P76149 | 4.4E-26 | -1.58 | Succinate semialdehyde dehydrogenase [NAD(P)+] Sad (SSADH) (SSDH) (EC 1.2.1.16) |
|  | *cydA* | P0ABJ9 | 1.7E-38 | -1.58 | Cytochrome bd-I ubiquinol oxidase subunit 1 (EC 7.1.1.7) (Cytochrome bd-I oxidase subunit I) (Cytochrome d ubiquinol oxidase subunit I) |
|  | *pabA* | P00903 | 8.7E-08 | -1.57 | Aminodeoxychorismate synthase component 2 (ADC synthase) (ADCS) (EC 2.6.1.85) (4-amino-4-deoxychorismate synthase component 2) (Aminodeoxychorismate synthase, glutamine amidotransferase component) |
|  | *bamC* | P0A903 | 1.9E-11 | -1.57 | Outer membrane protein assembly factor BamC |
|  | *can* | P61517 | 3.3E-22 | -1.56 | Carbonic anhydrase 2 (EC 4.2.1.1) (Carbonate dehydratase 2) |
|  | *alaC* | P77434 | 1.7E-09 | -1.56 | Glutamate-pyruvate aminotransferase AlaC (EC 2.6.1.2) |
|  | *rplD* | P60723 | 9.4E-11 | -1.55 | 50S ribosomal protein L4 (Large ribosomal subunit protein uL4) |
|  | *yjhH* | P39359 | 1.4E-04 | -1.54 | Probable 2-dehydro-3-deoxy-D-pentonate aldolase YjhH (EC 4.1.2.28) |
|  | *sfmD* | P77468 | 3.4E-34 | -1.54 | Outer membrane usher protein SfmD |
|  | *srlD* | P05707 | 4.0E-18 | -1.54 | Sorbitol-6-phosphate 2-dehydrogenase (EC 1.1.1.140) (Glucitol-6-phosphate dehydrogenase) (Ketosephosphate reductase) |
|  | *tolR* | P0ABV6 | 1.9E-04 | -1.54 | Tol-Pal system protein TolR |
|  | *cbrB* | P31468 | 5.4E-05 | -1.53 | Inner membrane protein CbrB (CreB-regulated gene B protein) |
|  | *fmt* | P23882 | 8.5E-25 | -1.53 | Methionyl-tRNA formyltransferase (EC 2.1.2.9) (Met-tRNA(fMet) formyltransferase) |
|  | *ptsP* | P37177 | 1.2E-07 | -1.52 | Phosphoenolpyruvate-dependent phosphotransferase system (EC 2.7.3.9) (Enzyme I-Ntr) (EINtr) (Phosphotransferase system, enzyme I) |
|  | *yacG* | P0A8H8 | 1.1E-18 | -1.52 | DNA gyrase inhibitor YacG |
|  | *acrF* | P24181 | 1.3E-04 | -1.52 | Multidrug export protein AcrF (Acriflavine resistance protein F) (Protein EnvD) |
|  | *ompT* | P09169 | 6.3E-09 | -1.52 | Protease 7 (EC 3.4.23.49) (Omptin) (Outer membrane protein 3B) (Protease A) (Protease VII) |
|  | *gatZ* | P0C8J8 | 2.4E-08 | -1.51 | D-tagatose-1,6-bisphosphate aldolase subunit GatZ |
|  | *fdx* | P0A9R4 | 1.3E-03 | -1.51 | 2Fe-2S ferredoxin |
|  | *sgbE* | P37680 | 2.1E-12 | -1.51 | L-ribulose-5-phosphate 4-epimerase SgbE (EC 5.1.3.4) (Phosphoribulose isomerase) |
|  | *fumB* | P14407 | 1.9E-22 | -1.51 | Fumarate hydratase class I, anaerobic (EC 4.2.1.2) (D-tartrate dehydratase) (EC 4.2.1.81) (Fumarase B) |
|  | *folK* | P26281 | 2.5E-05 | -1.51 | 2-amino-4-hydroxy-6-hydroxymethyldihydropteridine pyrophosphokinase (EC 2.7.6.3) (6-hydroxymethyl-7,8-dihydropterin pyrophosphokinase) (PPPK) (7,8-dihydro-6-hydroxymethylpterin-pyrophosphokinase) (HPPK) |
|  | *nanC* | P69856 | 3.9E-11 | -1.51 | Probable N-acetylneuraminic acid outer membrane channel protein NanC (NanR-regulated channel) (Porin NanC) |
|  | *dusC* | P33371 | 1.1E-12 | -1.51 | tRNA-dihydrouridine(16) synthase (EC 1.3.1.-) (U16-specific dihydrouridine synthase) (U16-specific Dus) (tRNA-dihydrouridine synthase C) |
|  | *ampD* | P13016 | 4.2E-05 | -1.50 | 1,6-anhydro-N-acetylmuramyl-L-alanine amidase AmpD (EC 3.5.1.28) (N-acetylmuramoyl-L-alanine amidase) |
|  | *aas* | P31119 | 5.2E-19 | -1.50 | Bifunctional protein Aas [Includes: 2-acylglycerophosphoethanolamine acyltransferase (EC 2.3.1.40) (2-acyl-GPE acyltransferase) (Acyl-[acyl-carrier-protein]--phospholipid O-acyltransferase); Acyl-[acyl-carrier-protein] synthetase (EC 6.2.1.20) (Acyl-ACP synthetase) (Long-chain-fatty-acid--[acyl-carrier-protein] ligase)] |
|  | *rpsU* | P68679 | 7.9E-08 | -1.50 | 30S ribosomal protein S21 (Small ribosomal subunit protein bS21) |
|  | *kdpD* | P21865 | 1.1E-04 | -1.49 | Sensor protein KdpD (EC 2.7.13.3) |
|  | *def* | P0A6K3 | 1.1E-22 | -1.49 | Peptide deformylase (PDF) (EC 3.5.1.88) (Polypeptide deformylase) |
|  | *msrQ* | P76343 | 8.5E-13 | -1.49 | Protein-methionine-sulfoxide reductase heme-binding subunit MsrQ (Flavocytochrome MsrQ) |
|  | *ymgC* | P75994 | 2.3E-09 | -1.48 | Uncharacterized protein YmgC |
|  | *dtpA* | P77304 | 4.0E-26 | -1.48 | Dipeptide and tripeptide permease A |
|  | *rplX* | P60624 | 4.6E-05 | -1.48 | 50S ribosomal protein L24 (Large ribosomal subunit protein uL24) |
|  | *nirC* | P0AC26 | 1.6E-10 | -1.48 | Nitrite transporter NirC |
|  | *rnlA* | P52129 | 1.4E-18 | -1.48 | mRNA endoribonuclease toxin LS (EC 3.1.-.-) (RNase LS) (Toxin LS) |
|  | *ldhA* | P52643 | 1.6E-13 | -1.48 | D-lactate dehydrogenase (D-LDH) (EC 1.1.1.28) (Fermentative lactate dehydrogenase) |
|  | *nanS* | P39370 | 4.1E-15 | -1.48 | Probable 9-O-acetyl-N-acetylneuraminic acid deacetylase (Neu5,9Ac2 deacetylase) (EC 3.1.1.-) (Probable 9-O-acetyl-N-acetylneuraminate esterase) (Probable sialyl esterase NanS) |
|  | *xanQ* | P67444 | 3.8E-08 | -1.47 | Xanthine permease XanQ |
|  | *rna* | P21338 | 2.0E-09 | -1.47 | Ribonuclease I (RNase I) (EC 4.6.1.21) (Enterobacter ribonuclease) |
|  | *psiE* | P0A7C8 | 4.8E-11 | -1.46 | Protein PsiE |
|  | *paaI* | P76084 | 1.3E-06 | -1.46 | Acyl-coenzyme A thioesterase PaaI (EC 3.1.2.-) (Phenylacetic acid degradation protein PaaI) |
|  | *apaH* | P05637 | 1.4E-06 | -1.46 | Bis(5'-nucleosyl)-tetraphosphatase [symmetrical] (EC 3.6.1.41) (Ap4A hydrolase) (Diadenosine 5',5'''-P1,P4-tetraphosphate pyrophosphohydrolase) (Diadenosine tetraphosphatase) |
|  | *appY* | P05052 | 2.0E-09 | -1.45 | HTH-type transcriptional regulator AppY (M5 polypeptide) |
|  | *thyA* | P0A884 | 2.4E-20 | -1.45 | Thymidylate synthase (TS) (TSase) (EC 2.1.1.45) |
|  | *ybaL* | P39830 | 1.1E-05 | -1.45 | Putative cation/proton antiporter YbaL |
|  | *yidG* | P0ADL6 | 3.0E-06 | -1.45 | Inner membrane protein YidG |
|  | *yadV* | P33128 | 1.2E-11 | -1.45 | Probable fimbrial chaperone YadV |
|  | *rimK* | P0C0U4 | 2.2E-06 | -1.45 | Ribosomal protein S6--L-glutamate ligase (EC 6.3.2.-) (Polyglutamate synthase) (Ribosomal protein S6 modification protein) |
|  | *yecM* | P52007 | 4.3E-09 | -1.45 | Protein YecM |
|  | *hyaB* | P0ACD8 | 2.1E-03 | -1.44 | Hydrogenase-1 large chain (HYD1) (EC 1.12.99.6) (Membrane-bound hydrogenase 1 large subunit) (NiFe hydrogenase) |
|  | *glcB* | P37330 | 1.5E-03 | -1.44 | Malate synthase G (MSG) (EC 2.3.3.9) |
|  | *pxpB* | P0AAV4 | 3.8E-06 | -1.44 | 5-oxoprolinase subunit B (5-OPase subunit B) (EC 3.5.2.9) (5-oxoprolinase (ATP-hydrolyzing) subunit B) |
|  | *dapA* | P0A6L2 | 1.0E-14 | -1.44 | 4-hydroxy-tetrahydrodipicolinate synthase (HTPA synthase) (EC 4.3.3.7) |
|  | *sfmH* | P75715 | 3.3E-04 | -1.44 | Uncharacterized fimbrial-like protein SfmH |
|  | *yejM* | P0AD27 | 2.9E-12 | -1.44 | Inner membrane protein YejM |
|  | *glnD* | P27249 | 2.5E-26 | -1.43 | Bifunctional uridylyltransferase/uridylyl-removing enzyme (UTase/UR) (Bifunctional [protein-PII] modification enzyme) (Bifunctional nitrogen sensor protein) [Includes: [Protein-PII] uridylyltransferase (PII uridylyltransferase) (UTase) (EC 2.7.7.59); [Protein-PII]-UMP uridylyl-removing enzyme (UR) (EC 3.1.4.-)] |
|  | *yehC* | P33342 | 1.6E-11 | -1.43 | Probable fimbrial chaperone YehC |
|  | *treC* | P28904 | 6.6E-13 | -1.43 | Trehalose-6-phosphate hydrolase (EC 3.2.1.93) (Alpha,alpha-phosphotrehalase) |
|  | *cbeA* | P76364 | 1.4E-21 | -1.43 | Cytoskeleton bundling-enhancing antitoxin CbeA (Antitoxin CbeA) (Antitoxin YeeU) |
|  | *ybcO* | P68661 | 1.5E-03 | -1.42 | Putative nuclease YbcO (EC 3.1.-.-) |
|  | *yedK* | P76318 | 1.2E-11 | -1.42 | Abasic site processing protein YedK (EC 3.4.-.-) |
|  | *sdiA* | P07026 | 9.9E-25 | -1.41 | Regulatory protein SdiA |
|  | *ybhA* | P21829 | 1.4E-05 | -1.41 | Pyridoxal phosphate phosphatase YbhA (PLP phosphatase) (EC 3.1.3.74) |
|  | *ykgR* | C1P5Z8 | 9.2E-04 | -1.41 | Uncharacterized membrane protein YkgR |
|  | *rcsC* | P0DMC5 | 4.7E-16 | -1.40 | Sensor histidine kinase RcsC (EC 2.7.13.3) (Capsular synthesis regulator component C) |
|  | *kbaY* | P0AB74 | 2.3E-07 | -1.40 | D-tagatose-1,6-bisphosphate aldolase subunit KbaY (TBPA) (TagBP aldolase) (EC 4.1.2.40) (D-tagatose-bisphosphate aldolase class II) (Ketose 1,6-bisphosphate aldolase class II) (Tagatose-bisphosphate aldolase) |
|  | *fbp* | P0A993 | 3.9E-14 | -1.39 | Fructose-1,6-bisphosphatase class 1 (FBPase class 1) (EC 3.1.3.11) (D-fructose-1,6-bisphosphate 1-phosphohydrolase class 1) |
|  | *yfhM* | P76578 | 3.2E-05 | -1.39 | Alpha-2-macroglobulin (ECAM) |
|  | *malK* | P68187 | 7.4E-17 | -1.38 | Maltose/maltodextrin import ATP-binding protein MalK (EC 7.5.2.1) |
|  | *nupG* | P0AFF4 | 2.8E-13 | -1.38 | Nucleoside permease NupG (Nucleoside-transport system protein NupG) |
|  | *yshB* | C1P620 | 1.6E-02 | -1.38 | Uncharacterized protein YshB |
|  | *prpR* | P77743 | 5.6E-08 | -1.37 | Propionate catabolism operon regulatory protein |
|  | *speC* | P21169 | 5.4E-11 | -1.37 | Constitutive ornithine decarboxylase (EC 4.1.1.17) |
|  | *queD* | P65870 | 2.4E-04 | -1.37 | 6-carboxy-5,6,7,8-tetrahydropterin synthase (CPH4 synthase) (EC 4.1.2.50) (Queuosine biosynthesis protein QueD) |
|  | *btuC* | P06609 | 6.0E-31 | -1.37 | Vitamin B12 import system permease protein BtuC |
|  | *hxpA* | P77625 | 8.6E-13 | -1.37 | Hexitol phosphatase A (Mannitol-1-phosphatase) (EC 3.1.3.22) (Sorbitol-6-phosphatase) (EC 3.1.3.50) (Sugar-phosphatase) (EC 3.1.3.23) |
|  | *rhaT* | P27125 | 7.3E-05 | -1.36 | L-rhamnose-proton symporter (L-rhamnose-H(+) transport protein) |
|  | *lysO* | P75826 | 9.1E-06 | -1.36 | Lysine exporter LysO (Lys outward permease) |
|  | *cysJ* | P38038 | 3.7E-21 | -1.36 | Sulfite reductase [NADPH] flavoprotein alpha-component (SiR-FP) (EC 1.8.1.2) |
|  | *seqA* | P0AFY8 | 2.0E-08 | -1.36 | Negative modulator of initiation of replication |
|  | *xseB* | P0A8G9 | 4.1E-20 | -1.36 | Exodeoxyribonuclease 7 small subunit (EC 3.1.11.6) (Exodeoxyribonuclease VII small subunit) (Exonuclease VII small subunit) |
|  | *srlE* | P56580 | 3.6E-11 | -1.36 | PTS system glucitol/sorbitol-specific EIIB component (EC 2.7.1.198) (EII-Gut) (Enzyme II-Gut) (Glucitol/sorbitol-specific phosphotransferase enzyme IIB component) |
|  | *wrbA* | P0A8G6 | 5.0E-10 | -1.36 | NAD(P)H dehydrogenase (quinone) (EC 1.6.5.2) (Flavoprotein WrbA) (NAD(P)H:quinone oxidoreductase) (NQO) |
|  | *ygfK* | Q46811 | 2.8E-28 | -1.35 | Putative oxidoreductase YgfK (Putative oxidoreductase Fe-S subunit) |
|  | *mhpB* | P0ABR9 | 1.3E-04 | -1.35 | 2,3-dihydroxyphenylpropionate/2,3-dihydroxicinnamic acid 1,2-dioxygenase (EC 1.13.11.16) (3-carboxyethylcatechol 2,3-dioxygenase) |
|  | *nfsB* | P38489 | 9.9E-04 | -1.35 | Oxygen-insensitive NAD(P)H nitroreductase (EC 1.-.-.-) (Dihydropteridine reductase) (EC 1.5.1.34) (FMN-dependent nitroreductase) |
|  | *lgt* | P60955 | 7.1E-09 | -1.35 | Phosphatidylglycerol--prolipoprotein diacylglyceryl transferase (EC 2.5.1.145) (Prolipoprotein diacylglyceryl transferase) |
|  | *alx* | P42601 | 4.5E-09 | -1.35 | Putative membrane-bound redox modulator Alx |
|  | *dmsA* | P18775 | 2.9E-08 | -1.35 | Dimethyl sulfoxide reductase DmsA (DMSO reductase) (DMSOR) (Me2SO reductase) (EC 1.8.5.3) |
|  | *flhB* | P76299 | 1.8E-05 | -1.35 | Flagellar biosynthetic protein FlhB |
|  | *ompL* | P76773 | 8.3E-16 | -1.35 | Porin OmpL |
|  | *torT* | P38683 | 3.0E-10 | -1.35 | Periplasmic protein TorT |
|  | *nrfB* | P0ABL1 | 4.3E-06 | -1.34 | Cytochrome c-type protein NrfB |
|  | *ftsB* | P0A6S5 | 1.7E-09 | -1.34 | Cell division protein FtsB |
|  | *narX* | P0AFA2 | 1.0E-09 | -1.34 | Nitrate/nitrite sensor protein NarX (EC 2.7.13.3) |
|  | *sapF* | P0AAH8 | 3.2E-20 | -1.34 | Putrescine export system ATP-binding protein SapF |
|  | *gspD* | P45758 | 1.4E-07 | -1.34 | Putative secretin GspD (Putative general secretion pathway protein D) (Putative type II secretion system protein D) (T2SS protein D) |
|  | *ispD* | Q46893 | 7.3E-11 | -1.34 | 2-C-methyl-D-erythritol 4-phosphate cytidylyltransferase (EC 2.7.7.60) (4-diphosphocytidyl-2C-methyl-D-erythritol synthase) (CDP-ME synthase) (MEP cytidylyltransferase) (MCT) |
|  | *ycjP* | P77716 | 1.9E-04 | -1.34 | Inner membrane ABC transporter permease protein YcjP |
|  | *lpxP* | P0ACV2 | 8.8E-09 | -1.33 | Lipid A biosynthesis palmitoleoyltransferase (EC 2.3.1.242) (Kdo(2)-lipid IV(A) palmitoleoyltransferase) |
|  | *nrdD* | P28903 | 7.8E-04 | -1.33 | Anaerobic ribonucleoside-triphosphate reductase (EC 1.1.98.6) (Class III ribonucleoside-triphosphate reductase) |
|  | *ttcA* | P76055 | 1.6E-09 | -1.32 | tRNA-cytidine(32) 2-sulfurtransferase (EC 2.8.1.-) (Two-thiocytidine biosynthesis protein A) (tRNA 2-thiocytidine biosynthesis protein TtcA) |
|  | *manA* | P00946 | 1.4E-06 | -1.31 | Mannose-6-phosphate isomerase (EC 5.3.1.8) (Phosphohexomutase) (Phosphomannose isomerase) (PMI) |
|  | *caiC* | P31552 | 6.3E-06 | -1.31 | Crotonobetaine/carnitine--CoA ligase (EC 6.2.1.48) (Betaine:CoA ligase) |
|  | *suhB* | P0ADG4 | 9.3E-05 | -1.30 | Nus factor SuhB (Inositol-1-monophosphatase) (I-1-Pase) (IMPase) (Inositol-1-phosphatase) (EC 3.1.3.25) |
|  | *ghxP* | P0AF52 | 7.4E-09 | -1.30 | Guanine/hypoxanthine permease GhxP |
|  | *adiC* | P60061 | 3.8E-08 | -1.30 | Arginine/agmatine antiporter |
|  | *rplE* | P62399 | 3.2E-10 | -1.30 | 50S ribosomal protein L5 (Large ribosomal subunit protein uL5) |
|  | *spy* | P77754 | 1.1E-03 | -1.29 | Periplasmic chaperone Spy (Spheroplast protein Y) |
|  | *alsE* | P32719 | 9.2E-08 | -1.29 | D-allulose-6-phosphate 3-epimerase (EC 5.1.3.-) |
|  | *epmB* | P39280 | 9.7E-03 | -1.29 | L-lysine 2,3-aminomutase (LAM) (EC 5.4.3.-) (EF-P post-translational modification enzyme B) |
|  | *rplT* | P0A7L3 | 2.7E-14 | -1.29 | 50S ribosomal protein L20 (Large ribosomal subunit protein bL20) |
|  | *yegT* | P76417 | 1.3E-06 | -1.28 | Putative nucleoside transporter YegT |
|  | *puuC* | P23883 | 3.0E-04 | -1.28 | NADP/NAD-dependent aldehyde dehydrogenase PuuC (ALDH) (EC 1.2.1.5) (3-hydroxypropionaldehyde dehydrogenase) (Gamma-glutamyl-gamma-aminobutyraldehyde dehydrogenase) (Gamma-Glu-gamma-aminobutyraldehyde dehydrogenase) |
|  | *mltC* | P0C066 | 1.1E-14 | -1.28 | Membrane-bound lytic murein transglycosylase C (EC 4.2.2.n1) (Murein lyase C) |
|  | *yhcB* | P0ADW3 | 3.3E-13 | -1.27 | Inner membrane protein YhcB |
|  | *basS* | P30844 | 7.8E-16 | -1.27 | Sensor protein BasS (EC 2.7.13.3) |
|  | *dcyD* | P76316 | 2.6E-03 | -1.27 | D-cysteine desulfhydrase (EC 4.4.1.15) |
|  | *yhdJ* | P28638 | 1.7E-02 | -1.27 | DNA adenine methyltransferase YhdJ (EC 2.1.1.72) |
|  | *emrD* | P31442 | 3.1E-14 | -1.26 | Multidrug resistance protein D |
|  | *spoT* | P0AG24 | 1.5E-10 | -1.26 | Bifunctional (p)ppGpp synthase/hydrolase SpoT [Includes: GTP pyrophosphokinase (EC 2.7.6.5) ((p)ppGpp synthase) (ATP:GTP 3'-pyrophosphotransferase) (Stringent response-like protein) (ppGpp synthase II); Guanosine-3',5'-bis(diphosphate) 3'-pyrophosphohydrolase (EC 3.1.7.2) (Penta-phosphate guanosine-3'-pyrophosphohydrolase) ((ppGpp)ase)] |
|  | *baeR* | P69228 | 5.5E-04 | -1.26 | Transcriptional regulatory protein BaeR |
|  | *prfA* | P0A7I0 | 8.8E-04 | -1.26 | Peptide chain release factor RF1 (RF-1) |
|  | *hsdM* | P08957 | 4.9E-20 | -1.25 | Type I restriction enzyme EcoKI M protein (M.EcoKI) (EC 2.1.1.72) |
|  | *lsrC* | P77672 | 4.8E-24 | -1.25 | Autoinducer 2 import system permease protein LsrC (AI-2 import system permease protein LsrC) |
|  | *yqjG* | P42620 | 1.6E-08 | -1.25 | Glutathionyl-hydroquinone reductase YqjG (GS-HQR) (EC 1.8.5.7) |
|  | *trkA* | P0AGI8 | 1.6E-31 | -1.25 | Trk system potassium uptake protein TrkA (K(+)-uptake protein TrkA) |
|  | *ybjN* | P0AAY6 | 5.1E-10 | -1.25 | Uncharacterized protein YbjN |
|  | *hscA* | P0A6Z1 | 8.6E-11 | -1.24 | Chaperone protein HscA (Hsc66) |
|  | *murQ* | P76535 | 1.0E-04 | -1.24 | N-acetylmuramic acid 6-phosphate etherase (MurNAc-6-P etherase) (EC 4.2.1.126) (N-acetylmuramic acid 6-phosphate hydrolase) (N-acetylmuramic acid 6-phosphate lyase) |
|  | *cusF* | P77214 | 5.1E-09 | -1.24 | Cation efflux system protein CusF |
|  | *yedF* | P0AA31 | 2.0E-04 | -1.24 | Putative sulfur carrier protein YedF |
|  | *rpsH* | P0A7W7 | 7.8E-15 | -1.24 | 30S ribosomal protein S8 (Small ribosomal subunit protein uS8) |
|  | *gadC* | P63235 | 2.4E-09 | -1.24 | Glutamate/gamma-aminobutyrate antiporter (Glu/GABA antiporter) (Extreme acid sensitivity protein) |
|  | *rpoA* | P0A7Z4 | 4.5E-43 | -1.24 | DNA-directed RNA polymerase subunit alpha (RNAP subunit alpha) (EC 2.7.7.6) (RNA polymerase subunit alpha) (Transcriptase subunit alpha) |
|  | *chaB* | P0AE63 | 1.4E-16 | -1.23 | Putative cation transport regulator ChaB |
|  | *nrfA* | P0ABK9 | 2.1E-13 | -1.23 | Cytochrome c-552 (EC 1.7.2.2) (Ammonia-forming cytochrome c nitrite reductase) (Cytochrome c nitrite reductase) |
|  | *ltaE* | P75823 | 4.7E-08 | -1.23 | Low specificity L-threonine aldolase (Low specificity L-TA) (EC 4.1.2.48) |
|  | *rplQ* | P0AG44 | 4.5E-29 | -1.23 | 50S ribosomal protein L17 (Large ribosomal subunit protein bL17) |
|  | *fliF* | P25798 | 4.7E-19 | -1.23 | Flagellar M-ring protein |
|  | *ybiT* | P0A9U3 | 1.6E-21 | -1.23 | Probable ATP-binding protein YbiT |
|  | *dppD* | P0AAG0 | 3.6E-04 | -1.23 | Dipeptide transport ATP-binding protein DppD (EC 7.4.2.9) |
|  | *ychN* | P0AB52 | 9.8E-06 | -1.23 | Protein YchN |
|  | *nikC* | P0AFA9 | 6.1E-05 | -1.23 | Nickel transport system permease protein NikC |
|  | *grxA* | P68688 | 8.3E-03 | -1.22 | Glutaredoxin 1 (Grx1) |
|  | *yhgF* | P46837 | 8.7E-08 | -1.22 | Protein YhgF |
|  | *yfdC* | P37327 | 1.5E-05 | -1.22 | Inner membrane protein YfdC |
|  | *entF* | P11454 | 1.2E-04 | -1.21 | Enterobactin synthase component F (EC 2.7.7.-) (Enterochelin synthase F) (Serine-activating enzyme) (Seryl-AMP ligase) |
|  | *lsrD* | P0AFS1 | 2.9E-09 | -1.21 | Autoinducer 2 import system permease protein LsrD (AI-2 import system permease protein LsrD) |
|  | *nudI* | P52006 | 1.0E-10 | -1.21 | Nucleoside triphosphatase NudI (EC 3.6.1.9) (Nucleotide diphosphatase NudI) (Pyrimidine deoxynucleoside triphosphate diphosphatase) (dCTP diphosphatase) (EC 3.6.1.12) (dTTP diphosphatase) (EC 3.6.1.-) (dUTP diphosphatase) (EC 3.6.1.23) |
|  | *hemL* | P23893 | 4.8E-07 | -1.21 | Glutamate-1-semialdehyde 2,1-aminomutase (GSA) (EC 5.4.3.8) (Glutamate-1-semialdehyde aminotransferase) (GSA-AT) |
|  | *ynfE* | P77374 | 2.9E-36 | -1.21 | Putative dimethyl sulfoxide reductase chain YnfE (DMSO reductase) (EC 1.8.99.-) |
|  | *yoaG* | P64496 | 3.8E-03 | -1.21 | Protein YoaG |
|  | *oppD* | P76027 | 6.6E-03 | -1.21 | Oligopeptide transport ATP-binding protein OppD |
|  | *allE* | P75713 | 8.1E-06 | -1.20 | (S)-ureidoglycine aminohydrolase (UGHY) (UGlyAH) (EC 3.5.3.26) |
|  | *ssuA* | P75853 | 3.8E-09 | -1.20 | Putative aliphatic sulfonates-binding protein |
|  | *hycI* | P0AEV9 | 3.7E-06 | -1.20 | Hydrogenase 3 maturation protease (EC 3.4.23.51) (HycI protease) |
|  | *trmJ* | P0AE01 | 2.7E-15 | -1.20 | tRNA (cytidine/uridine-2'-O-)-methyltransferase TrmJ (EC 2.1.1.200) (TrMet(Xm32)) (tRNA (cytidine(32)/uridine(32)-2'-O)-methyltransferase) (tRNA Cm32/Um32 methyltransferase) |
|  | *aldA* | P25553 | 2.0E-03 | -1.20 | Lactaldehyde dehydrogenase (EC 1.2.1.22) (Aldehyde dehydrogenase A) (Glycolaldehyde dehydrogenase) (EC 1.2.1.21) |
|  | *sseA* | P31142 | 5.6E-03 | -1.20 | 3-mercaptopyruvate sulfurtransferase (MST) (EC 2.8.1.2) (Rhodanese-like protein) |
|  | *rpsK* | P0A7R9 | 1.3E-11 | -1.19 | 30S ribosomal protein S11 (Small ribosomal subunit protein uS11) |
|  | *rutG* | P75892 | 3.2E-07 | -1.19 | Putative pyrimidine permease RutG |
|  | *rplF* | P0AG55 | 3.0E-12 | -1.19 | 50S ribosomal protein L6 (Large ribosomal subunit protein uL6) |
|  | *appC* | P26459 | 2.5E-10 | -1.19 | Cytochrome bd-II ubiquinol oxidase subunit 1 (EC 7.1.1.3) (Cytochrome bd-II oxidase subunit I) |
|  | *ycaL* | P43674 | 5.6E-04 | -1.19 | Metalloprotease YcaL (EC 3.4.-.-) |
|  | *garR* | P0ABQ2 | 3.0E-11 | -1.19 | 2-hydroxy-3-oxopropionate reductase (EC 1.1.1.60) (Tartronate semialdehyde reductase) (TSAR) |
|  | *pspF* | P37344 | 8.5E-03 | -1.19 | Psp operon transcriptional activator (Phage shock protein F) |
|  | *yagF* | P77596 | 1.4E-21 | -1.18 | D-xylonate dehydratase YagF (EC 4.2.1.82) |
|  | *fumA* | P0AC33 | 1.2E-08 | -1.18 | Fumarate hydratase class I, aerobic (EC 4.2.1.2) (Fumarase A) (Oxaloacetate keto--enol-isomerase) (OAAKE isomerase) (Oxaloacetate tautomerase) (EC 5.3.2.2) |
|  | *chaC* | P39163 | 2.6E-14 | -1.17 | Glutathione-specific gamma-glutamylcyclotransferase (Gamma-GCG) (EC 4.3.2.7) (Cation transport regulatory protein ChaC) |
|  | *mlaA* | P76506 | 7.6E-07 | -1.17 | Intermembrane phospholipid transport system lipoprotein MlaA |
|  | *rsmB* | P36929 | 9.5E-41 | -1.17 | Ribosomal RNA small subunit methyltransferase B (EC 2.1.1.176) (16S rRNA m5C967 methyltransferase) (rRNA (cytosine-C(5)-)-methyltransferase RsmB) |
|  | *nanM* | P39371 | 1.0E-06 | -1.17 | N-acetylneuraminate epimerase (EC 5.1.3.24) (N-acetylneuraminate mutarotase) (Neu5Ac mutarotase) (Sialic acid epimerase) |
|  | *eptA* | P30845 | 4.0E-12 | -1.17 | Phosphoethanolamine transferase EptA (EC 2.7.-.-) (Polymyxin resistance protein PmrC) |
|  | *ygcP* | Q46906 | 1.3E-04 | -1.17 | Uncharacterized protein YgcP |
|  | *pppA* | Q46836 | 3.3E-05 | -1.16 | Prepilin peptidase PppA |
|  | *yadK* | P37016 | 2.5E-04 | -1.16 | Uncharacterized fimbrial-like protein YadK |
|  | *yfeZ* | P76538 | 5.7E-12 | -1.16 | Inner membrane protein YfeZ |
|  | *mscL* | P0A742 | 3.1E-17 | -1.16 | Large-conductance mechanosensitive channel |
|  | *msrP* | P76342 | 1.2E-09 | -1.16 | Protein-methionine-sulfoxide reductase catalytic subunit MsrP (EC 1.8.5.-) |
|  | *hslR* | P0ACG8 | 2.1E-09 | -1.16 | Heat shock protein 15 (HSP15) |
|  | *ilvB* | P08142 | 1.4E-07 | -1.16 | Acetolactate synthase isozyme 1 large subunit (AHAS-I) (EC 2.2.1.6) (Acetohydroxy-acid synthase I large subunit) (ALS-I) |
|  | *yigB* | P0ADP0 | 3.3E-02 | -1.16 | 5-amino-6-(5-phospho-D-ribitylamino)uracil phosphatase YigB (EC 3.1.3.104) |
|  | *zntR* | P0ACS5 | 2.0E-25 | -1.15 | HTH-type transcriptional regulator ZntR (Zn(II)-responsive regulator of zntA) |
|  | *rimL* | P13857 | 3.6E-09 | -1.15 | Ribosomal-protein-serine acetyltransferase (EC 2.3.1.-) (Acetylating enzyme for N-terminal of ribosomal protein L7/L12) |
|  | *yehD* | P33343 | 9.7E-07 | -1.15 | Uncharacterized fimbrial-like protein YehD |
|  | *fklB* | P0A9L3 | 3.6E-03 | -1.15 | FKBP-type 22 kDa peptidyl-prolyl cis-trans isomerase (FKBP22) (PPIase) (EC 5.2.1.8) (Rotamase) |
|  | *yecD* | P0ADI7 | 2.6E-04 | -1.15 | Isochorismatase family protein YecD (EC 3.-.-.-) |
|  | *ydcF* | P34209 | 3.2E-03 | -1.15 | Protein YdcF |
|  | *rpmD* | P0AG51 | 1.7E-13 | -1.15 | 50S ribosomal protein L30 (Large ribosomal subunit protein uL30) |
|  | *folB* | P0AC16 | 5.7E-03 | -1.15 | Dihydroneopterin aldolase (DHNA) (EC 4.1.2.25) (7,8-dihydroneopterin 2'-epimerase) (7,8-dihydroneopterin aldolase) (7,8-dihydroneopterin epimerase) (EC 5.1.99.8) (Dihydroneopterin epimerase) |
|  | *potD* | P0AFK9 | 4.7E-05 | -1.14 | Spermidine/putrescine-binding periplasmic protein (SPBP) |
|  | *rpsM* | P0A7S9 | 6.3E-13 | -1.14 | 30S ribosomal protein S13 (Small ribosomal subunit protein uS13) |
|  | *cheZ* | P0A9H9 | 7.0E-05 | -1.14 | Protein phosphatase CheZ (EC 3.1.3.-) (Chemotaxis protein CheZ) |
|  | *pykA* | P21599 | 1.6E-04 | -1.14 | Pyruvate kinase II (EC 2.7.1.40) (PK-2) |
|  | *pgk* | P0A799 | 4.7E-03 | -1.14 | Phosphoglycerate kinase (EC 2.7.2.3) |
|  | *yafO* | Q47157 | 7.5E-08 | -1.14 | mRNA interferase toxin YafO (EC 3.1.-.-) (Endoribonuclease YafO) (Toxin YafO) |
|  | *nadC* | P30011 | 1.6E-06 | -1.14 | Nicotinate-nucleotide pyrophosphorylase [carboxylating] (EC 2.4.2.19) (Quinolinate phosphoribosyltransferase [decarboxylating]) (QAPRTase) |
|  | *recR* | P0A7H6 | 1.2E-06 | -1.13 | Recombination protein RecR |
|  | *cusR* | P0ACZ8 | 9.7E-07 | -1.13 | Transcriptional regulatory protein CusR |
|  | *yggX* | P0A8P3 | 3.7E-07 | -1.13 | Probable Fe(2+)-trafficking protein |
|  | *tehA* | P25396 | 1.2E-12 | -1.13 | Tellurite resistance protein TehA |
|  | *gspG* | P41442 | 2.5E-11 | -1.12 | Type II secretion system core protein G (T2SS core protein G) (Protein transport protein HofG) (Putative general secretion pathway protein G) |
|  | *agaS* | P42907 | 3.6E-09 | -1.12 | Putative D-galactosamine-6-phosphate deaminase AgaS (EC 3.5.99.-) (Gam-6-P deaminase/isomerase) |
|  | *ybjM* | P64439 | 1.0E-02 | -1.12 | Inner membrane protein YbjM |
|  | *rnr* | P21499 | 2.9E-03 | -1.12 | Ribonuclease R (RNase R) (EC 3.1.13.1) (Protein VacB) |
|  | *fliG* | P0ABZ1 | 4.8E-09 | -1.12 | Flagellar motor switch protein FliG |
|  | *sdaB* | P30744 | 3.7E-19 | -1.12 | L-serine dehydratase 2, SDH 2, EC 4.3.1.17 |
|  | *prpB* | P77541 | 5.0E-07 | -1.12 | 2-methylisocitrate lyase (2-MIC) (MICL) (EC 4.1.3.30) ((2R,3S)-2-methylisocitrate lyase) |
|  | *dhaM* | P37349 | 1.1E-15 | -1.12 | PEP-dependent dihydroxyacetone kinase, phosphoryl donor subunit DhaM (EC 2.7.1.121) (Dihydroxyacetone kinase subunit M) |
|  | *torY* | P52005 | 1.0E-10 | -1.12 | Cytochrome c-type protein TorY |
|  | *trkG* | P23849 | 7.4E-06 | -1.12 | Trk system potassium uptake protein TrkG |
|  | *yhjE* | P37643 | 2.7E-04 | -1.11 | Inner membrane metabolite transport protein YhjE |
|  | *yabI* | P30149 | 1.3E-12 | -1.11 | Inner membrane protein YabI |
|  | *hprR* | P76340 | 1.1E-03 | -1.11 | Transcriptional regulatory protein HprR (Hydrogen peroxide response regulator) |
|  | *galR* | P03024 | 1.4E-05 | -1.11 | HTH-type transcriptional regulator GalR (Galactose operon repressor) |
|  | *ves* | P76214 | 3.2E-03 | -1.11 | Protein Ves (Various environmental stresses-induced protein) |
|  | *ftsP* | P26648 | 2.0E-03 | -1.11 | Cell division protein FtsP |
|  | *adeP* | P31466 | 1.7E-04 | -1.11 | Adenine permease AdeP |
|  | *rpsN* | P0AG59 | 4.7E-09 | -1.11 | 30S ribosomal protein S14 (Small ribosomal subunit protein uS14) |
|  | *eutC* | P19636 | 4.0E-06 | -1.11 | Ethanolamine ammonia-lyase light chain (EC 4.3.1.7) (Ethanolamine ammonia-lyase small subunit) |
|  | *cspB* | P36995 | 1.5E-06 | -1.11 | Cold shock-like protein CspB (CSP-B) |
|  | *purC* | P0A7D7 | 1.0E-07 | -1.11 | Phosphoribosylaminoimidazole-succinocarboxamide synthase (EC 6.3.2.6) (SAICAR synthetase) |
|  | *ycjX* | P76046 | 6.8E-04 | -1.10 | Uncharacterized protein YcjX |
|  | *mtfA* | P76346 | 1.3E-08 | -1.10 | Protein MtfA (Mlc titration factor A) |
|  | *hcp* | P75825 | 1.8E-05 | -1.10 | Hydroxylamine reductase (EC 1.7.99.1) (Hybrid-cluster protein) (HCP) (Prismane protein) |
|  | *glnH* | P0AEQ3 | 3.6E-03 | -1.10 | Glutamine-binding periplasmic protein (GlnBP) |
|  | *torS* | P39453 | 1.3E-04 | -1.10 | Sensor protein TorS (EC 2.7.13.3) |
|  | *hycA* | P0AEV4 | 5.6E-05 | -1.10 | Formate hydrogenlyase regulatory protein HycA |
|  | *nuoB* | P0AFC7 | 1.2E-04 | -1.10 | NADH-quinone oxidoreductase subunit B (EC 7.1.1.-) (NADH dehydrogenase I subunit B) (NDH-1 subunit B) (NUO2) |
|  | *pspA* | P0AFM6 | 1.2E-02 | -1.09 | Phage shock protein A |
|  | *yidZ* | P31463 | 4.6E-09 | -1.09 | HTH-type transcriptional regulator YidZ |
|  | *yphA* | P0AD47 | 1.4E-04 | -1.09 | Inner membrane protein YphA |
|  | *priB* | P07013 | 2.8E-03 | -1.09 | Primosomal replication protein N |
|  | *nfsA* | P17117 | 1.0E-02 | -1.09 | Oxygen-insensitive NADPH nitroreductase (EC 1.-.-.-) (Modulator of drug activity A) |
|  | *rpsE* | P0A7W1 | 6.7E-14 | -1.09 | 30S ribosomal protein S5 (Small ribosomal subunit protein uS5) |
|  | *prlF* | P15373 | 7.0E-03 | -1.09 | Antitoxin PrlF (HtrA suppressor protein SohA) |
|  | *gspH* | P41443 | 3.1E-09 | -1.09 | Type II secretion system protein H (T2SS minor pseudopilin H) (General secretion pathway protein H) (Protein transport protein HofH) (Putative general secretion pathway protein H) |
|  | *rpmE* | P0A7M9 | 7.0E-08 | -1.09 | 50S ribosomal protein L31 (Large ribosomal subunit protein bL31-A) |
|  | *ybdG* | P0AAT4 | 1.0E-04 | -1.08 | Miniconductance mechanosensitive channel YbdG |
|  | *mnmC* | P77182 | 5.4E-17 | -1.08 | tRNA 5-methylaminomethyl-2-thiouridine biosynthesis bifunctional protein MnmC (tRNA mnm(5)s(2)U biosynthesis bifunctional protein) [Includes: tRNA (mnm(5)s(2)U34)-methyltransferase (EC 2.1.1.61); FAD-dependent cmnm(5)s(2)U34 oxidoreductase (EC 1.5.-.-)] |
|  | *rplB* | P60422 | 1.2E-06 | -1.08 | 50S ribosomal protein L2 (Large ribosomal subunit protein uL2) |
|  | *hyaC* | P0AAM1 | 1.1E-02 | -1.08 | Probable Ni/Fe-hydrogenase 1 B-type cytochrome subunit |
|  | *glcC* | P0ACL5 | 4.4E-07 | -1.08 | Glc operon transcriptional activator (Glc regulatory protein) (HTH-type transcriptional regulator GlcC) |
|  | *damX* | P11557 | 2.9E-08 | -1.07 | Cell division protein DamX |
|  | *yidC* | P25714 | 2.7E-08 | -1.07 | Membrane protein insertase YidC (Foldase YidC) (Inner membrane protein YidC) (Membrane integrase YidC) (Oxa1Ec) |
|  | *fdhD* | P32177 | 1.9E-03 | -1.07 | Sulfur carrier protein FdhD (Sulfurtransferase FdhD) |
|  | *yjeO* | P39284 | 9.2E-11 | -1.07 | Inner membrane protein YjeO |
|  | *puuR* | P0A9U6 | 2.1E-03 | -1.07 | HTH-type transcriptional regulator PuuR |
|  | *paaJ* | P0C7L2 | 7.9E-11 | -1.07 | 3-oxoadipyl-CoA/3-oxo-5,6-dehydrosuberyl-CoA thiolase (EC 2.3.1.174) (EC 2.3.1.223) |
|  | *osmE* | P0ADB1 | 9.9E-06 | -1.07 | Osmotically-inducible putative lipoprotein OsmE (Activator of ntr-like gene protein) |
|  | *atoB* | P76461 | 7.1E-05 | -1.07 | Acetyl-CoA acetyltransferase (EC 2.3.1.9) (Acetoacetyl-CoA thiolase) |
|  | *sfmF* | P38052 | 2.2E-03 | -1.07 | Uncharacterized fimbrial-like protein SfmF |
|  | *glnB* | P0A9Z1 | 2.2E-02 | -1.07 | Nitrogen regulatory protein P-II 1 |
|  | *psuG* | P33025 | 4.2E-07 | -1.06 | Pseudouridine-5'-phosphate glycosidase (PsiMP glycosidase) (EC 4.2.1.70) |
|  | *yebT* | P76272 | 1.8E-04 | -1.06 | Intermembrane transport protein YebT |
|  | *cysI* | P17846 | 2.3E-11 | -1.06 | Sulfite reductase [NADPH] hemoprotein beta-component (SiR-HP) (SiRHP) (EC 1.8.1.2) |
|  | *arfA* | P36675 | 1.5E-19 | -1.06 | Alternative ribosome-rescue factor A |
|  | *greB* | P30128 | 2.7E-11 | -1.05 | Transcription elongation factor GreB (Transcript cleavage factor GreB) |
|  | *rpsD* | P0A7V8 | 8.9E-21 | -1.05 | 30S ribosomal protein S4 (Small ribosomal subunit protein uS4) |
|  | *secY* | P0AGA2 | 1.3E-16 | -1.05 | Protein translocase subunit SecY |
|  | *hyaE* | P19931 | 3.2E-03 | -1.05 | Hydrogenase-1 operon protein HyaE |
|  | *mmuM* | Q47690 | 5.9E-05 | -1.05 | Homocysteine S-methyltransferase (EC 2.1.1.10) (S-methylmethionine:homocysteine methyltransferase) |
|  | *ssuD* | P80645 | 9.7E-07 | -1.05 | Alkanesulfonate monooxygenase (EC 1.14.14.5) (FMNH2-dependent aliphatic sulfonate monooxygenase) (Sulfate starvation-induced protein 6) (SSI6) |
|  | *rplR* | P0C018 | 3.6E-11 | -1.05 | 50S ribosomal protein L18 (Large ribosomal subunit protein uL18) |
|  | *leuE* | P76249 | 4.9E-04 | -1.05 | Leucine efflux protein |
|  | *uspG* | P39177 | 1.5E-06 | -1.05 | Universal stress protein UP12 (Universal stress protein G) |
|  | *yehL* | P33348 | 2.0E-09 | -1.04 | Uncharacterized protein YehL |
|  | *serC* | P23721 | 3.2E-08 | -1.04 | Phosphoserine aminotransferase (EC 2.6.1.52) (Phosphohydroxythreonine aminotransferase) (PSAT) |
|  | *ptsN* | P69829 | 4.8E-03 | -1.04 | Nitrogen regulatory protein (Enzyme IIA-NTR) (PTS system EIIA component) (Phosphotransferase enzyme IIA component) |
|  | *ybcI* | P45570 | 3.9E-02 | -1.04 | Inner membrane protein YbcI |
|  | *sppA* | P08395 | 1.8E-10 | -1.04 | Protease 4 (EC 3.4.21.-) (Endopeptidase IV) (Protease IV) (Signal peptide peptidase) |
|  | *rplO* | P02413 | 2.5E-14 | -1.03 | 50S ribosomal protein L15 (Large ribosomal subunit protein uL15) |
|  | *arnB* | P77690 | 1.8E-12 | -1.03 | UDP-4-amino-4-deoxy-L-arabinose--oxoglutarate aminotransferase (EC 2.6.1.87) (Polymyxin resistance protein PmrH) (UDP-(beta-L-threo-pentapyranosyl-4''-ulose diphosphate) aminotransferase) (UDP-Ara4O aminotransferase) (UDP-4-amino-4-deoxy-L-arabinose aminotransferase) |
|  | *gabD* | P25526 | 2.5E-06 | -1.03 | Succinate-semialdehyde dehydrogenase [NADP(+)] GabD (SSDH) (EC 1.2.1.79) (Glutarate-semialdehyde dehydrogenase) (EC 1.2.1.-) |
|  | *htpX* | P23894 | 1.1E-09 | -1.03 | Protease HtpX (EC 3.4.24.-) (Heat shock protein HtpX) |
|  | *hyuA* | Q46806 | 1.4E-09 | -1.03 | D-phenylhydantoinase (EC 3.5.2.-) (Hydantoin-utilizing enzyme HyuA) |
|  | *rlmG* | P42596 | 1.7E-10 | -1.03 | Ribosomal RNA large subunit methyltransferase G (EC 2.1.1.174) (23S rRNA m2G1835 methyltransferase) (rRNA (guanine-N(2)-)-methyltransferase RlmG) |
|  | *ygdG* | P38506 | 1.9E-05 | -1.03 | Flap endonuclease Xni (FEN) (EC 3.1.-.-) (Exonuclease IX) (ExoIX) |
|  | *yejE* | P33915 | 9.5E-13 | -1.03 | Inner membrane ABC transporter permease protein YejE |
|  | *ravA* | P31473 | 1.1E-03 | -1.03 | ATPase RavA (EC 3.6.3.-) (Regulatory ATPase variant A) |
|  | *ydgH* | P76177 | 9.6E-06 | -1.03 | Protein YdgH |
|  | *rplC* | P60438 | 5.8E-05 | -1.03 | 50S ribosomal protein L3 (Large ribosomal subunit protein uL3) |
|  | *araB* | P08204 | 4.1E-06 | -1.03 | Ribulokinase (EC 2.7.1.16) |
|  | *nanT* | P41036 | 1.1E-07 | -1.02 | Sialic acid transporter NanT (Sialic acid permease) (Sialic acid/H(+) symporter) |
|  | *yiaC* | P37664 | 5.9E-06 | -1.02 | Peptidyl-lysine N-acetyltransferase YiaC (EC 2.3.1.-) (KAT) |
|  | *pdxK* | P40191 | 2.3E-07 | -1.02 | Pyridoxine/pyridoxal/pyridoxamine kinase (PN/PL/PM kinase) (EC 2.7.1.35) (B6-vitamer kinase) (Pyridoxal kinase 1) (PL kinase 1) |
|  | *prmA* | P0A8T1 | 1.0E-13 | -1.02 | Ribosomal protein L11 methyltransferase (L11 Mtase) (EC 2.1.1.-) |
|  | *yagA* | P37007 | 5.4E-10 | -1.02 | Uncharacterized protein YagA |
|  | *yidA* | P0A8Y5 | 9.9E-09 | -1.01 | Sugar phosphatase YidA (EC 3.1.3.23) |
|  | *relB* | P0C079 | 4.0E-04 | -1.01 | Antitoxin RelB |
|  | *ygjP* | P42597 | 4.3E-09 | -1.01 | UTP pyrophosphatase (EC 3.6.1.-) |
|  | *paaD* | P76080 | 1.0E-03 | -1.01 | Putative 1,2-phenylacetyl-CoA epoxidase, subunit D (1,2-phenylacetyl-CoA monooxygenase, subunit D) |
|  | *ftsH* | P0AAI3 | 2.1E-05 | -1.01 | ATP-dependent zinc metalloprotease FtsH (EC 3.4.24.-) (Cell division protease FtsH) |
|  | *feaR* | Q47129 | 3.3E-03 | -1.01 | Transcriptional activator FeaR |
|  | *uhpA* | P0AGA6 | 4.1E-06 | -1.01 | Transcriptional regulatory protein UhpA |
|  | *gspI* | P45760 | 1.3E-07 | -1.01 | Putative type II secretion system protein I (T2SS minor pseudopilin I) (Putative general secretion pathway protein I) |
|  | *ftsX* | P0AC30 | 6.9E-03 | -1.00 | Cell division protein FtsX |
|  | *phnI* | P16687 | 8.7E-04 | -1.00 | Alpha-D-ribose 1-methylphosphonate 5-triphosphate synthase subunit PhnI (RPnTP synthase subunit PhnI) (EC 2.7.8.37) (Ribose 1-methylphosphonate 5-triphosphate synthase nucleosidase subunit) |
