# Supplementary material for: Effects of NaCl Concentrations on Growth Patterns, Phenotypes Associated With Virulence, and Energy Metabolism in Escherichia coli BW25113
Source: Front Microbiol. 2021 Aug 16;12:705326. doi: 10.3389/fmicb.2021.705326 (PMC8415458; doi:10.3389/fmicb.2021.705326)
Supplement: Supplementary file 5 [file Table_2.docx]

**Supplementary Table 2** Top 20 up- and down-regulated genes in transcriptomic analysis when compared high salinity groups (3.5% and 5% NaCl) with low salinity group (0% and 1% NaCl).

|  | **Gene Name** | **UniProt ID** | ***P*-value** | **log2(Fold_Change)** | **UniProt Annotation** | **Length** | **Status** |
| --- | --- | --- | --- | --- | --- | --- | --- |
| **Up-regulated Genes** | *stpA* | P0ACG1 | 2.23E-57 | 8.09 | DNA-binding protein StpA (H-NS homolog StpA) | 134 | reviewed |
|  | *kch* | P31069 | 1.89E-31 | 7.76 | Voltage-gated potassium channel Kch | 417 | reviewed |
|  | *ygaM* | P0ADQ7 | 6.19E-34 | 7.19 | Uncharacterized protein YgaM | 109 | reviewed |
|  | *alaE* | P64550 | 2.0E-32 | 6.90 | L-alanine exporter AlaE | 149 | reviewed |
|  | *wcaJ* | P71241 | 2.12E-08 | 6.37 | UDP-glucose:undecaprenyl-phosphate glucose-1-phosphate transferase (UDP-Glc:Und-P Glc-1-P transferase) (EC 2.7.8.31) (Colanic acid biosynthesis UDP-glucose lipid carrier transferase) (Glucosyl-P-P-undecaprenol synthase) | 464 | reviewed |
|  | *nrdH* | P0AC65 | 2.24E-20 | 6.36 | Glutaredoxin-like protein NrdH | 81 | reviewed |
|  | *leuC* | P0A6A6 | 4.03E-09 | 6.10 | 3-isopropylmalate dehydratase large subunit (EC 4.2.1.33) (Alpha-IPM isomerase) (IPMI) (Isopropylmalate isomerase) | 466 | reviewed |
|  | *chbA* | P69791 | 1.78E-23 | 6.00 | PTS system N,N'-diacetylchitobiose-specific EIIA component (EIIA-Chb) (EIII-Chb) (IIIcel) (N,N'-diacetylchitobiose-specific phosphotransferase enzyme IIA component) | 116 | reviewed |
|  | *evgS* | P30855 | 8.86E-120 | 5.91 | Sensor protein EvgS (EC 2.7.13.3) | 1197 | reviewed |
|  | *recQ* | P15043 | 4.86E-10 | 5.82 | ATP-dependent DNA helicase RecQ (EC 3.6.4.12) | 609 | reviewed |
|  | *yegS* | P76407 | 4.48E-25 | 5.71 | Lipid kinase YegS (EC 2.7.1.-) | 299 | reviewed |
|  | *leuD* | P30126 | 7.81E-09 | 5.71 | 3-isopropylmalate dehydratase small subunit (EC 4.2.1.33) (Alpha-IPM isomerase) (IPMI) (Isopropylmalate isomerase) | 201 | reviewed |
|  | *fcl* | P32055 | 9.63E-07 | 5.42 | GDP-L-fucose synthase (EC 1.1.1.271) (GDP-4-keto-6-deoxy-D-mannose-3,5-epimerase-4-reductase) | 321 | reviewed |
|  | *nrdI* | P0A772 | 4.91E-16 | 5.35 | Protein NrdI | 136 | reviewed |
|  | *yceO* | P64442 | 3.31E-67 | 5.33 | Uncharacterized protein YceO | 46 | reviewed |
|  | *gmm* | P32056 | 4.49E-06 | 5.23 | GDP-mannose mannosyl hydrolase (GDPMH) (EC 3.6.1.-) (Colanic acid biosynthesis protein WcaH) | 159 | reviewed |
|  | *ygaV* | P77295 | 1.05E-23 | 5.09 | Probable HTH-type transcriptional regulator YgaV | 99 | reviewed |
|  | *thrA* | P00561 | 1.58E-07 | 4.95 | Bifunctional aspartokinase/homoserine dehydrogenase 1 (Aspartokinase I/homoserine dehydrogenase I) (AKI-HDI) [Includes: Aspartokinase (EC 2.7.2.4); Homoserine dehydrogenase (EC 1.1.1.3)] | 820 | reviewed |
|  | *ygaP* | P55734 | 6.68E-11 | 4.95 | Inner membrane protein YgaP | 174 | reviewed |
|  | *kbp* | P0ADE6 | 7.28E-24 | 4.92 | Potassium binding protein Kbp (K(+) binding protein Kbp) | 149 | reviewed |
|  | **Gene Name** | **UniProt ID** | **P-value** | **log2(Fold_Change)** | **UniProt Annotation** | **Length** | **Status** |
| **Down-regulated Genes** | *glrK* | P52101 | 5.98E-134 | -4.50 | Sensor histidine kinase GlrK (EC 2.7.13.3) | 475 | reviewed |
|  | *tolB* | P0A855 | 3.44E-33 | -4.29 | Tol-Pal system protein TolB | 430 | reviewed |
|  | *pmbA* | P0AFK0 | 8.92E-194 | -4.29 | Metalloprotease PmbA (EC 3.4.-.-) (Protein TldE) | 450 | reviewed |
|  | *nikA* | P33590 | 1.46E-68 | -3.90 | Nickel-binding periplasmic protein | 524 | reviewed |
|  | *mhpE* | P51020 | 4.47E-27 | -3.79 | 4-hydroxy-2-oxovalerate aldolase (HOA) (EC 4.1.3.39) (4-hydroxy-2-keto-pentanoic acid aldolase) (4-hydroxy-2-oxopentanoate aldolase) | 337 | reviewed |
|  | *yniD* | Q2EES1 | 5.11E-43 | -3.77 | Uncharacterized protein YniD | 35 | reviewed |
|  | *yliI* | P75804 | 1.302E-63 | -3.61 | Aldose sugar dehydrogenase YliI (Asd) (EC 1.1.5.-) (Soluble aldose sugar dehydrogenase YliI) | 371 | reviewed |
|  | *ybeZ* | P0A9K3 | 1.36E-17 | -3.42 | PhoH-like protein | 346 | reviewed |
|  | *kdpB* | P03960 | 3.47E-31 | -3.40 | Potassium-transporting ATPase ATP-binding subunit (EC 7.2.2.6) (ATP phosphohydrolase [potassium-transporting] B chain) (Potassium-binding and translocating subunit B) (Potassium-translocating ATPase B chain) | 682 | reviewed |
|  | *sapC* | P0AGH5 | 4.69E-17 | -3.39 | Putrescine export system permease protein SapC | 296 | reviewed |
|  | *ubiC* | P26602 | 1.62E-17 | -3.36 | Chorismate pyruvate-lyase (CL) (CPL) (EC 4.1.3.40) | 165 | reviewed |
|  | *slyA* | P0A8W2 | 1.24E-58 | -3.34 | Transcriptional regulator SlyA | 144 | reviewed |
|  | *tauA* | Q47537 | 7.58E-06 | -3.34 | Taurine-binding periplasmic protein (Sulfate starvation-induced protein 1) (SSI1) | 320 | reviewed |
|  | *nrdG* | P0A9N8 | 1.08E-48 | -3.33 | Anaerobic ribonucleoside-triphosphate reductase-activating protein (EC 1.97.1.-) (Class III anaerobic ribonucleotide reductase small component) | 154 | reviewed |
|  | *thiE* | P30137 | 2.61E-56 | -3.28 | Thiamine-phosphate synthase (TP synthase) (TPS) (EC 2.5.1.3) (Thiamine-phosphate pyrophosphorylase) (TMP pyrophosphorylase) (TMP-PPase) | 211 | reviewed |
|  | *tolA* | P19934 | 9.34E-15 | -3.28 | Tol-Pal system protein TolA | 421 | reviewed |
|  | *modB* | P0AF01 | 2.30E-23 | -3.28 | Molybdenum transport system permease protein ModB | 229 | reviewed |
|  | *ydfR* | P76160 | 1.42E-37 | -3.27 | Uncharacterized protein YdfR | 103 | reviewed |
|  | *ybeY* | P0A898 | 3.28E-15 | -3.21 | Endoribonuclease YbeY (EC 3.1.-.-) | 155 | reviewed |
|  | *putA* | P09546 | 1.33E-26 | -3.19 | Bifunctional protein PutA [Includes: Proline dehydrogenase (EC 1.5.5.2) (Proline oxidase); Delta-1-pyrroline-5-carboxylate dehydrogenase (P5C dehydrogenase) (EC 1.2.1.88) (L-glutamate gamma-semialdehyde dehydrogenase)] | 1320 | reviewed |
