# Supplementary material for: Effects of NaCl Concentrations on Growth Patterns, Phenotypes Associated With Virulence, and Energy Metabolism in Escherichia coli BW25113
Source: Front Microbiol. 2021 Aug 16;12:705326. doi: 10.3389/fmicb.2021.705326 (PMC8415458; doi:10.3389/fmicb.2021.705326)
Supplement: Supplementary file 6 [file Table_3.docx]

**Supplementary Table 3** Top 20 hub nodes (proteins) with highest degrees in protein-protein interactive networks for up-regulated genes and down-regulated genes, respectively

|  | **Gene Name** | **UniProt ID** | ***P*-value** | **log2(Fold_Change)** | **Degree** | **UniProt Annotation** | **Length** | **Status** |
| --- | --- | --- | --- | --- | --- | --- | --- | --- |
| **Up-regulated Genes** | *ppsA* | P23538 | 2.85E-08 | 1.28 | 58 | Phosphoenolpyruvate synthase (PEP synthase) (EC 2.7.9.2) (Pyruvate, water dikinase) | 792 | reviewed |
|  | *evgS* | P30855 | 8.86E-120 | 5.91 | 57 | Sensor protein EvgS (EC 2.7.13.3) | 1197 | reviewed |
|  | *metH* | P13009 | 1.61E-07 | 4.08 | 50 | Methionine synthase (EC 2.1.1.13) (5-methyltetrahydrofolate--homocysteine methyltransferase) (Methionine synthase, vitamin-B12-dependent) (MS) | 1227 | reviewed |
|  | *thrA* | P00561 | 1.58E-07 | 4.95 | 43 | Bifunctional aspartokinase/homoserine dehydrogenase 1 (Aspartokinase I/homoserine dehydrogenase I) (AKI-HDI) [Includes: Aspartokinase (EC 2.7.2.4); Homoserine dehydrogenase (EC 1.1.1.3)] | 820 | reviewed |
|  | *ftsZ* | P0A9A6 | 1.26E-15 | 2.05 | 40 | Cell division protein FtsZ | 383 | reviewed |
|  | *tpiA* | P0A858 | 0.0002 | 1.41 | 39 | Triosephosphate isomerase (TIM) (TPI) (EC 5.3.1.1) (Triose-phosphate isomerase) | 255 | reviewed |
|  | *ompF* | P02931 | 4.93E-43 | 1.40 | 38 | Outer membrane porin F (Outer membrane protein 1A) (Outer membrane protein B) (Outer membrane protein F) (Outer membrane protein IA) (Porin OmpF) | 362 | reviewed |
|  | *arcB* | P0AEC3 | 2.88E-09 | 2.14 | 37 | Aerobic respiration control sensor protein ArcB (EC 2.7.13.3) | 778 | reviewed |
|  | *gltA* | P0ABH7 | 8.93E-28 | 2.48 | 33 | Citrate synthase (EC 2.3.3.16) | 427 | reviewed |
|  | *pykF* | P0AD61 | 1.80E-06 | 2.80 | 29 | Pyruvate kinase I (EC 2.7.1.40) (PK-1) | 470 | reviewed |
|  | *purB* | P0AB89 | 1.90E-08 | 2.16 | 29 | Adenylosuccinate lyase (ASL) (EC 4.3.2.2) (Adenylosuccinase) (ASase) | 456 | reviewed |
|  | *hisB* | P06987 | 6.73E-16 | 1.91 | 28 | Histidine biosynthesis bifunctional protein HisB [Includes: Histidinol-phosphatase (EC 3.1.3.15); Imidazoleglycerol-phosphate dehydratase (IGPD) (EC 4.2.1.19)] | 355 | reviewed |
|  | *ftsK* | P46889 | 9.21E-14 | 2.21 | 27 | DNA translocase FtsK | 1329 | reviewed |
|  | *rsmH* | P60390 | 2.15E-12 | 2.61 | 27 | Ribosomal RNA small subunit methyltransferase H (EC 2.1.1.199) (16S rRNA m(4)C1402 methyltransferase) (rRNA (cytosine-N(4)-)-methyltransferase RsmH) | 313 | reviewed |
|  | *tdcE* | P42632 | 6.23E-10 | 2.13 | 26 | PFL-like enzyme TdcE (Keto-acid formate acetyltransferase) (Keto-acid formate-lyase) (Ketobutyrate formate-lyase) (KFL) (EC 2.3.1.-) (Pyruvate formate-lyase) (PFL) (EC 2.3.1.54) | 764 | reviewed |
|  | *gyrA* | P0AES4 | 5.20E-132 | 3.23 | 26 | DNA gyrase subunit A (EC 5.6.2.2) | 875 | reviewed |
|  | *purA* | P0A7D4 | 8.35E-21 | 1.95 | 26 | Adenylosuccinate synthetase (AMPSase) (AdSS) (EC 6.3.4.4) (IMP--aspartate ligase) | 432 | reviewed |
|  | *ftsI* | P0AD68 | 1.79E-10 | 2.68 | 26 | Peptidoglycan D,D-transpeptidase FtsI (EC 3.4.16.4) (Essential cell division protein FtsI) (Murein transpeptidase) (Penicillin-binding protein 3) (PBP-3) (Peptidoglycan synthase FtsI) | 588 | reviewed |
|  | *purH* | P15639 | 4.68E-26 | 3.64 | 25 | Bifunctional purine biosynthesis protein PurH [Includes: Phosphoribosylaminoimidazolecarboxamide formyltransferase (EC 2.1.2.3) (AICAR transformylase); IMP cyclohydrolase (EC 3.5.4.10) (ATIC) (IMP synthase) (Inosinicase)] | 529 | reviewed |
|  | *pyrF* | P08244 | 1.79E-08 | 1.54 | 25 | Orotidine 5'-phosphate decarboxylase (EC 4.1.1.23) (OMP decarboxylase) (OMPDCase) (OMPdecase) | 245 | reviewed |
|  | **Gene Name** | **UniProt ID** | **P-value** | **log2(Fold_Change)** | **Degree** | **UniProt Annotation** | **Length** | **Status** |
| **Down-regulated Genes** | *metG* | P00959 | 1.98E-29 | -1.68 | 56 | Methionine--tRNA ligase (EC 6.1.1.10) (Methionyl-tRNA synthetase) (MetRS) | 677 | reviewed |
|  | *rpoA* | P0A7Z4 | 4.54E-43 | -1.24 | 56 | DNA-directed RNA polymerase subunit alpha (RNAP subunit alpha) (EC 2.7.7.6) (RNA polymerase subunit alpha) (Transcriptase subunit alpha) | 329 | reviewed |
|  | *rpmA* | P0A7L8 | 1.10E-59 | -2.82 | 49 | 50S ribosomal protein L27 (Large ribosomal subunit protein bL27) | 85 | reviewed |
|  | *rplE* | P62399 | 3.19E-10 | -1.30 | 49 | 50S ribosomal protein L5 (Large ribosomal subunit protein uL5) | 179 | reviewed |
|  | *rpsC* | P0A7V3 | 8.66E-14 | -2.06 | 49 | 30S ribosomal protein S3 (Small ribosomal subunit protein uS3) | 233 | reviewed |
|  | *rpsO* | P0ADZ4 | 2.934E-07 | -2.10 | 47 | 30S ribosomal protein S15 (Small ribosomal subunit protein uS15) | 89 | reviewed |
|  | *rpsD* | P0A7V8 | 8.93E-21 | -1.05 | 47 | 30S ribosomal protein S4 (Small ribosomal subunit protein uS4) | 206 | reviewed |
|  | *rpsK* | P0A7R9 | 1.34E-11 | -1.19 | 47 | 30S ribosomal protein S11 (Small ribosomal subunit protein uS11) | 129 | reviewed |
|  | *rpsE* | P0A7W1 | 6.70E-14 | -1.09 | 47 | 30S ribosomal protein S5 (Small ribosomal subunit protein uS5) | 167 | reviewed |
|  | *rplB* | P60422 | 1.18E-06 | -1.08 | 47 | 50S ribosomal protein L2 (Large ribosomal subunit protein uL2) | 273 | reviewed |
|  | *rplU* | P0AG48 | 6.30E-101 | -3.14 | 46 | 50S ribosomal protein L21 (Large ribosomal subunit protein bL21) | 103 | reviewed |
|  | *rplD* | P60723 | 9.39E-11 | -1.55 | 46 | 50S ribosomal protein L4 (Large ribosomal subunit protein uL4) | 201 | reviewed |
|  | *rplQ* | P0AG44 | 4.54E-29 | -1.23 | 45 | 50S ribosomal protein L17 (Large ribosomal subunit protein bL17) | 127 | reviewed |
|  | *rplC* | P60438 | 5.83E-05 | -1.03 | 45 | 50S ribosomal protein L3 (Large ribosomal subunit protein uL3) | 209 | reviewed |
|  | *rpsH* | P0A7W7 | 7.83E-15 | -1.24 | 44 | 30S ribosomal protein S8 (Small ribosomal subunit protein uS8) | 130 | reviewed |
|  | *rpsF* | P02358 | 1.53E-07 | -1.82 | 44 | 30S ribosomal protein S6 (Small ribosomal subunit protein bS6) [Cleaved into: 30S ribosomal protein S6, fully modified isoform; 30S ribosomal protein S6, non-modified isoform] | 135 | reviewed |
|  | *prfA* | P0A7I0 | 0.0009 | -1.26 | 44 | Peptide chain release factor RF1 (RF-1) | 360 | reviewed |
|  | *rplT* | P0A7L3 | 2.66E-14 | -1.29 | 43 | 50S ribosomal protein L20 (Large ribosomal subunit protein bL20) | 118 | reviewed |
|  | *rpsM* | P0A7S9 | 6.32E-13 | -1.14 | 43 | 30S ribosomal protein S13 (Small ribosomal subunit protein uS13) | 118 | reviewed |
|  | *rplO* | P02413 | 2.51E-14 | -1.03 | 43 | 50S ribosomal protein L15 (Large ribosomal subunit protein uL15) | 144 | reviewed |
