# Supplementary material for: Effects of NaCl Concentrations on Growth Patterns, Phenotypes Associated With Virulence, and Energy Metabolism in Escherichia coli BW25113
Source: Front Microbiol. 2021 Aug 16;12:705326. doi: 10.3389/fmicb.2021.705326 (PMC8415458; doi:10.3389/fmicb.2021.705326)
Supplement: Supplementary file 7 [file Table_4.docx]

**Supplementary Table 4** Top 20 hub nodes (proteins) via cytoHubba analysis in protein-protein interactive networks for high salinity groups (3.5% and 5% NaCl) and low salinity group (0% and 1% NaCl), respectively

|  | **Gene Name** | **UniProt ID** | ***P*-value** | **log2(Fold_Change)** | **Score** | **UniProt Annotation** | **Length** | **Status** |
| --- | --- | --- | --- | --- | --- | --- | --- | --- |
| **Up-regulated Genes** | *ftsZ* | P0A9A6 | 1.26E-15 | 2.05 | 4.79E+08 | Cell division protein FtsZ | 383 | reviewed |
|  | *ftsI* | P0AD68 | 1.79E-10 | 2.68 | 4.79E+08 | Peptidoglycan D,D-transpeptidase FtsI (EC 3.4.16.4) (Essential cell division protein FtsI) (Murein transpeptidase) (Penicillin-binding protein 3) (PBP-3) (Peptidoglycan synthase FtsI) | 588 | reviewed |
|  | *ftsW* | P0ABG4 | 1.58E-05 | 1.87 | 4.79E+08 | Probable peptidoglycan glycosyltransferase FtsW (PGT) (EC 2.4.1.129) (Cell division protein FtsW) (Cell wall polymerase) (Lipid II flippase FtsW) (Peptidoglycan polymerase) (PG polymerase) | 414 | reviewed |
|  | *ftsL* | P0AEN4 | 6.05E-13 | 2.62 | 4.79E+08 | Cell division protein FtsL | 121 | reviewed |
|  | *murF* | P11880 | 4.57E-05 | 2.33 | 4.79E+08 | UDP-N-acetylmuramoyl-tripeptide--D-alanyl-D-alanine ligase (EC 6.3.2.10) (D-alanyl-D-alanine-adding enzyme) (UDP-MurNAc-pentapeptide synthetase) | 452 | reviewed |
|  | *murG* | P17443 | 0.004 | 1.45 | 4.79E+08 | UDP-N-acetylglucosamine--N-acetylmuramyl-(pentapeptide) pyrophosphoryl-undecaprenol N-acetylglucosamine transferase (EC 2.4.1.227) (Undecaprenyl-PP-MurNAc-pentapeptide-UDPGlcNAc GlcNAc transferase) | 355 | reviewed |
|  | *murE* | P22188 | 5.48E-07 | 2.72 | 4.79E+08 | UDP-N-acetylmuramoyl-L-alanyl-D-glutamate--2,6-diaminopimelate ligase (EC 6.3.2.13) (Meso-A2pm-adding enzyme) (Meso-diaminopimelate-adding enzyme) (UDP-MurNAc-L-Ala-D-Glu:meso-diaminopimelate ligase) (UDP-MurNAc-tripeptide synthetase) (UDP-N-acetylmuramyl-tripeptide synthetase) | 495 | reviewed |
|  | *mraZ* | P22186 | 1.956E-10 | 2.29 | 4.79E+08 | Transcriptional regulator MraZ | 152 | reviewed |
|  | *rsmH* | P60390 | 2.15E-12 | 2.61 | 4.79E+08 | Ribosomal RNA small subunit methyltransferase H (EC 2.1.1.199) (16S rRNA m(4)C1402 methyltransferase) (rRNA (cytosine-N(4)-)-methyltransferase RsmH) | 313 | reviewed |
|  | *mraY* | P0A6W3 | 0.0002 | 1.98 | 4.79E+08 | Phospho-N-acetylmuramoyl-pentapeptide-transferase (EC 2.7.8.13) (UDP-MurNAc-pentapeptide phosphotransferase) | 360 | reviewed |
|  | *murC* | P17952 | 0.0002 | 1.29 | 4.79E+08 | UDP-N-acetylmuramate--L-alanine ligase (EC 6.3.2.8) (UDP-N-acetylmuramoyl-L-alanine synthetase) | 491 | reviewed |
|  | *murD* | P14900 | 1.89E-07 | 2.30 | 4.79E+08 | UDP-N-acetylmuramoylalanine--D-glutamate ligase (EC 6.3.2.9) (D-glutamic acid-adding enzyme) (UDP-N-acetylmuramoyl-L-alanyl-D-glutamate synthetase) | 438 | reviewed |
|  | *lpxC* | P0A725 | 2.81E-18 | 1.93 | 4.79E+08 | UDP-3-O-acyl-N-acetylglucosamine deacetylase (UDP-3-O-acyl-GlcNAc deacetylase) (EC 3.5.1.108) (Protein EnvA) (UDP-3-O-[R-3-hydroxymyristoyl]-N-acetylglucosamine deacetylase) | 305 | reviewed |
|  | *ftsK* | P46889 | 9.21E-14 | 2.21 | 41165 | DNA translocase FtsK | 1329 | reviewed |
|  | *moaA* | P30745 | 2.70E-10 | 1.39 | 40359 | GTP 3',8-cyclase (EC 4.1.99.22) (Molybdenum cofactor biosynthesis protein A) | 329 | reviewed |
|  | *moaE* | P30749 | 6.82E-13 | 1.79 | 40350 | Molybdopterin synthase catalytic subunit (EC 2.8.1.12) (MPT synthase subunit 2) (Molybdenum cofactor biosynthesis protein E) (Molybdopterin-converting factor large subunit) (Molybdopterin-converting factor subunit 2) | 150 | reviewed |
|  | *moaD* | P30748 | 2.06E-13 | 2.05 | 40350 | Molybdopterin synthase sulfur carrier subunit (MPT synthase subunit 1) (Molybdenum cofactor biosynthesis protein D) (Molybdopterin-converting factor small subunit) (Molybdopterin-converting factor subunit 1) (Sulfur carrier protein MoaD) | 81 | reviewed |
|  | *moaC* | P0A738 | 2.94E-20 | 2.03 | 40350 | Cyclic pyranopterin monophosphate synthase (EC 4.6.1.17) (Molybdenum cofactor biosynthesis protein C) | 161 | reviewed |
|  | *modA* | P37329 | 1.87E-13 | 1.52 | 40337 | Molybdate-binding protein ModA (Molybdate/tungstate-binding protein ModA) | 257 | reviewed |
|  | *mobA* | P32173 | 3.07E-20 | 1.75 | 40333 | Molybdenum cofactor guanylyltransferase (MoCo guanylyltransferase) (EC 2.7.7.77) (GTP:molybdopterin guanylyltransferase) (Mo-MPT guanylyltransferase) (Molybdopterin guanylyltransferase) (Molybdopterin-guanine dinucleotide biosynthesis protein A) (Molybdopterin-guanine dinucleotide synthase) (MGD synthase) (Protein FA) | 194 | reviewed |
|  | **Gene Name** | **UniProt ID** | **P-value** | **log2(Fold_Change)** | **Score** | **UniProt Annotation** | **Length** | **Status** |
| **Down-regulated Genes** | *rpsD* | P0A7V8 | 8.93E-21 | -1.05 | 1.55E+30 | 30S ribosomal protein S4 (Small ribosomal subunit protein uS4) | 206 | reviewed |
|  | *rpsE* | P0A7W1 | 6.70E-14 | -1.09 | 1.55E+30 | 30S ribosomal protein S5 (Small ribosomal subunit protein uS5) | 167 | reviewed |
|  | *rplE* | P62399 | 3.19E-10 | -1.30 | 1.55E+30 | 50S ribosomal protein L5 (Large ribosomal subunit protein uL5) | 179 | reviewed |
|  | *rplB* | P60422 | 1.18E-06 | -1.08 | 1.55E+30 | 50S ribosomal protein L2 (Large ribosomal subunit protein uL2) | 273 | reviewed |
|  | *rplD* | P60723 | 9.39E-11 | -1.55 | 1.55E+30 | 50S ribosomal protein L4 (Large ribosomal subunit protein uL4) | 201 | reviewed |
|  | *rplC* | P60438 | 5.83E-05 | -1.026331111 | 1.55E+30 | 50S ribosomal protein L3 (Large ribosomal subunit protein uL3) | 209 | reviewed |
|  | *rplQ* | P0AG44 | 4.54E-29 | -1.23 | 1.55E+30 | 50S ribosomal protein L17 (Large ribosomal subunit protein bL17) | 127 | reviewed |
|  | *rplO* | P02413 | 2.51E-14 | -1.03 | 1.55E+30 | 50S ribosomal protein L15 (Large ribosomal subunit protein uL15) | 144 | reviewed |
|  | *rpsK* | P0A7R9 | 1.34E-11 | -1.19 | 1.55E+30 | 30S ribosomal protein S11 (Small ribosomal subunit protein uS11) | 129 | reviewed |
|  | *rplF* | P0AG55 | 3.00E-12 | -1.19 | 1.55E+30 | 50S ribosomal protein L6 (Large ribosomal subunit protein uL6) | 177 | reviewed |
|  | *rpsC* | P0A7V3 | 8.66E-14 | -2.06 | 1.55E+30 | 30S ribosomal protein S3 (Small ribosomal subunit protein uS3) | 233 | reviewed |
|  | *rplW* | P0ADZ0 | 2.16E-09 | -1.65 | 1.55E+30 | 50S ribosomal protein L23 (Large ribosomal subunit protein uL23) | 100 | reviewed |
|  | *rpsM* | P0A7S9 | 6.32E-13 | -1.14 | 1.55E+30 | 30S ribosomal protein S13 (Small ribosomal subunit protein uS13) | 118 | reviewed |
|  | *rpsH* | P0A7W7 | 7.83E-15 | -1.24 | 1.55E+30 | 30S ribosomal protein S8 (Small ribosomal subunit protein uS8) | 130 | reviewed |
|  | *rpmD* | P0AG51 | 1.65E-13 | -1.15 | 1.55E+30 | 50S ribosomal protein L30 (Large ribosomal subunit protein uL30) | 59 | reviewed |
|  | *rplX* | P60624 | 4.58E-05 | -1.48 | 1.55E+30 | 50S ribosomal protein L24 (Large ribosomal subunit protein uL24) | 104 | reviewed |
|  | *rplV* | P61175 | 7.65E-18 | -2.55 | 1.55E+30 | 50S ribosomal protein L22 (Large ribosomal subunit protein uL22) | 110 | reviewed |
|  | *rpmA* | P0A7L8 | 1.10E-59 | -2.82 | 1.55E+30 | 50S ribosomal protein L27 (Large ribosomal subunit protein bL27) | 85 | reviewed |
|  | *rplU* | P0AG48 | 6.30E-101 | -3.14 | 1.55E+30 | 50S ribosomal protein L21 (Large ribosomal subunit protein bL21) | 103 | reviewed |
|  | *rplR* | P0C018 | 3.60E-11 | -1.05 | 1.55E+30 | 50S ribosomal protein L18 (Large ribosomal subunit protein uL18) | 117 | reviewed |
