# Supplementary material for: Effects of NaCl Concentrations on Growth Patterns, Phenotypes Associated With Virulence, and Energy Metabolism in Escherichia coli BW25113
Source: Front Microbiol. 2021 Aug 16;12:705326. doi: 10.3389/fmicb.2021.705326 (PMC8415458; doi:10.3389/fmicb.2021.705326)
Supplement: Supplementary file 8 [file Table_5.docx]

**Supplementary Table 5** Detailed information about GO term enrichment analysis of 1117 differentially expressed gene (DEGs)

| **Category** | **GO Term ID** | **GO Term** | **Gene Count** | **Gene Percentage (%)** | ***P*-value** |
| --- | --- | --- | --- | --- | --- |
| BP | GO:0046777 | protein autophosphorylation | 15 | 1.35 | 0.004 |
| BP | GO:0023014 | signal transduction by protein phosphorylation | 15 | 1.35 | 0.006 |
| BP | GO:0015949 | nucleobase-containing small molecule interconversion | 12 | 1.08 | 0.011 |
| BP | GO:0018106 | peptidyl-histidine phosphorylation | 14 | 1.26 | 0.012 |
| BP | GO:0006412 | translation | 29 | 2.61 | 0.014 |
| BP | GO:0009243 | O antigen biosynthetic process | 6 | 0.54 | 0.030 |
| BP | GO:0006099 | tricarboxylic acid cycle | 14 | 1.26 | 0.037 |
| BP | GO:0006189 | 'de novo' IMP biosynthetic process | 8 | 0.72 | 0.040 |
| BP | GO:0009447 | putrescine catabolic process | 7 | 0.63 | 0.049 |
| BP | GO:0006508 | proteolysis | 17 | 1.53 | 0.049 |
| BP | GO:0000160 | phosphorelay signal transduction system | 29 | 2.61 | 0.050 |
| CC | GO:0005829 | cytosol | 348 | 31.29 | 0.005 |
| CC | GO:0032153 | cell division site | 16 | 1.44 | 0.006 |
| CC | GO:0022625 | cytosolic large ribosomal subunit | 18 | 1.62 | 0.006 |
| MF | GO:0042802 | identical protein binding | 109 | 9.80 | 0.001 |
| MF | GO:0019843 | rRNA binding | 22 | 1.98 | 0.003 |
| MF | GO:0000155 | phosphorelay sensor kinase activity | 19 | 1.71 | 0.004 |
| MF | GO:0005515 | protein binding | 249 | 22.39 | 0.005 |
| MF | GO:0003723 | RNA binding | 35 | 3.15 | 0.005 |
| MF | GO:0003735 | structural constituent of ribosome | 28 | 2.52 | 0.006 |
| MF | GO:0008270 | zinc ion binding | 63 | 5.67 | 0.011 |
| MF | GO:0009982 | pseudouridine synthase activity | 9 | 0.81 | 0.011 |
| MF | GO:0000287 | magnesium ion binding | 69 | 6.21 | 0.018 |
| MF | GO:0030145 | manganese ion binding | 25 | 2.25 | 0.045 |
