# Supplementary material for: Effects of NaCl Concentrations on Growth Patterns, Phenotypes Associated With Virulence, and Energy Metabolism in Escherichia coli BW25113
Source: Front Microbiol. 2021 Aug 16;12:705326. doi: 10.3389/fmicb.2021.705326 (PMC8415458; doi:10.3389/fmicb.2021.705326)
Supplement: Supplementary file 9 [file Table_6.docx]

**Supplementary Table 6** Detailed information about KEGG pathway enrichment analyses of 653 up-regulated genes and 464 down-regulated

genes with statistical significance.

| **Group** | **Pathway ID** | **Pathway Term** | **Gene Count** | **Percentage %** | ***P*-value** |
| --- | --- | --- | --- | --- | --- |
| Up-regulated Genes | eco01100 | Metabolic pathways | 144 | 22.05 | 0.00056 |
|  | eco01110 | Biosynthesis of secondary metabolites | 76 | 11.64 | 0.00003 |
|  | eco01130 | Biosynthesis of antibiotics | 46 | 7.04 | 0.01471 |
|  | eco01230 | Biosynthesis of amino acids | 33 | 5.05 | 0.00357 |
|  | eco00240 | Pyrimidine metabolism | 21 | 3.22 | 0.00243 |
|  | eco00190 | Oxidative phosphorylation | 13 | 1.99 | 0.03166 |
|  | eco00260 | Glycine, serine and threonine metabolism | 12 | 1.84 | 0.03423 |
|  | eco00130 | Ubiquinone and other terpenoid-quinone biosynthesis | 8 | 1.23 | 0.04296 |
|  | eco00290 | Valine, leucine and isoleucine biosynthesis | 7 | 1.07 | 0.04321 |
|  | eco00523 | Polyketide sugar unit biosynthesis | 6 | 0.92 | 0.00077 |
|  | eco00521 | Streptomycin biosynthesis | 6 | 0.92 | 0.01021 |
| **Group** | **Pathway ID** | **Pathway Term** | **Gene Count** | **Percentage %** | ***P*-value** |
| Down-regulated Genes | eco03010 | Ribosome | 27 | 5.82 | 0.00005 |
|  | eco00920 | Sulfur metabolism | 10 | 2.16 | 0.04836 |
|  | eco00790 | Folate biosynthesis | 7 | 1.51 | 0.04769 |
