# Supplementary material for: Effects of NaCl Concentrations on Growth Patterns, Phenotypes Associated With Virulence, and Energy Metabolism in Escherichia coli BW25113
Source: Front Microbiol. 2021 Aug 16;12:705326. doi: 10.3389/fmicb.2021.705326 (PMC8415458; doi:10.3389/fmicb.2021.705326)
Supplement: Supplementary file 10 [file Table_7.docx]

**Supplementary Table 7** DEGs identified in this study whose mutations lead to strong repression of motility (swarming and swimming).

| **Gene Name** | **Function** | **Fold Change (Down-Regulated)** |
| --- | --- | --- |
| *tolB* | Periplasmic protein that interacts with the TolA-TolQ-TolR complex; required for uptake of group A colicins | -4.29 |
| *atpC* | Membrane-bound ATP synthase; F1 sector; epsilon-subunit | -2.28 |
| *atpH* | Membrane-bound ATP synthase; F1 sector; delta-subunit | -2.25 |
| *folP* | Dihydropteroate synthase | -1.76 |
| *cmk* | CMP kinase; multicopy suppressor of UMP kinase mutations | -1.69 |
| *pgm* | Phosphoglucomutase | -1.69 |
| *tolR* | Membrane spanning protein in TolA-TolQ-TolR complex | -1.54 |
| *rcsC* | Negative regulatory gene for capsule (colanic acid) synthesis; controls sliminess; contains TerF; probable histidine kinase | -1.4 |
| *flhB* | Flagellar export pore protein; member of type III (virulence-related) secretory pathway family (IIISP) | -1.35 |
| *fliF* | Flagellar basal body M-ring protein | -1.23 |
| *folB* | Dihydroneopterin aldolase | -1.15 |
| *cheZ* | Chemotactic signal transduction; flagellar regulon member | -1.14 |
| *fliG* | Motor switching and energizing | -1.12 |
| **Gene name** | **Function** | **Fold Change (Up-Regulated)** |
| *fliI* | Cytoplasmic membrane ATPase involved in flagellar assembly; involved in export of flagellar axial protein subunits | 3.41 |
| *hflD* | Downregulates lambda lysogenization; interacts with cII protein; non-essential first gene in purB operon; peripheral membrane protein | 2.08 |
| *cheA* | Autophosphorylating histidine protein kinase (sensor) of chemotactic response | 1.86 |
| *galU* | Glucose-1-P uridylyltransferase (UDP-glucose pyrophosphorylase) | 1.83 |
| *fliJ* | Flagellar biosynthesis | 1.66 |
| *rfaH* | Transcription antitermination factor that stimulates expression of LPS biosynthesis genes; F-factor sex pilus genes and hemolysin genes; | 1.14 |
| *motB* | Flagellar-regulon member; flagellar rotation | 1.09 |
| *rplA* | 50S ribosomal subunit protein L1 | 1.09 |
| *cheW* | Signal transduction; couples CheA to chemoreceptor control by promoting CheW/CheA/Tsr; 925 flagellar regulon | 1.07 |
| *rluD* | 23S rRNA pseudouridine synthase specific for the pseudouridines at positions 1911; 1915; and 1917; mutation supresses ftsH(ts) mutants | 1 |
